# Mapping and functional characterization of structural variation in 1060 pig genomes

**DOI:** 10.1186/s13059-024-03253-3

**Published:** 2024-05-07

**Authors:** Liu Yang, Hongwei Yin, Lijing Bai, Wenye Yao, Tan Tao, Qianyi Zhao, Yahui Gao, Jinyan Teng, Zhiting Xu, Qing Lin, Shuqi Diao, Zhangyuan Pan, Dailu Guan, Bingjie Li, Huaijun Zhou, Zhongyin Zhou, Fuping Zhao, Qishan Wang, Yuchun Pan, Zhe Zhang, Kui Li, Lingzhao Fang, George E. Liu

**Affiliations:** 1 Key Laboratory of Livestock and Poultry Multi-Omics of MARA, Genome Analysis Laboratory of the Ministry of Agriculture, Agricultural Genomics Institute at Shenzhen, Chinese Academy of Agricultural Sciences, Shenzhen, Guangdong China; 2 Animal Genomics and Improvement Laboratory, Beltsville Agricultural Research Center, Agricultural Research Service, USDA, Beltsville, MD 20705 USA; 3 State Key Laboratory of Swine and Poultry Breeding Industry, National Engineering Research Center for Breeding Swine Industry, Guangdong Provincial Key Lab of Agro-Animal Genomics and Molecular Breeding, College of Animal Science, South China Agricultural University, Guangzhou, China; 4 Department of Animal Science, University of California-Davis, Davis, CA USA; 5 Animal and Veterinary Sciences, Scotland’s Rural College (SRUC), Roslin Institute Building, Easter Bush, Midlothian, EH25 9RG United Kingdom; 6 State Key Laboratory of Genetic Resources and Evolution, Kunming Institute of Zoology, Chinese Academy of Sciences, Kunming, 650223 China; 7 Key Laboratory of Animal Genetics, Breeding and Reproduction (Poultry) of Ministry of Agriculture, Institute of Animal Sciences, Chinese Academy of Agricultural Sciences, Beijing, 100193 China; 8 Department of Animal Science, College of Animal Sciences, Zhejiang University, Hangzhou, 310058 China; 9 Center for Quantitative Genetics and Genomics, Aarhus University, Aarhus, Denmark

**Keywords:** Pig, Structure variation, Population diversity, Gene expression, Functional genome

## Abstract

**Background:**

Structural variations (SVs) have significant impacts on complex phenotypes by rearranging large amounts of DNA sequence.

**Results:**

We present a comprehensive SV catalog based on the whole-genome sequence of 1060 pigs (*Sus scrofa*) representing 101 breeds, covering 9.6% of the pig genome. This catalog includes 42,487 deletions, 37,913 mobile element insertions, 3308 duplications, 1664 inversions, and 45,184 break ends. Estimates of breed ancestry and hybridization using genotyped SVs align well with those from single nucleotide polymorphisms. Geographically stratified deletions are observed, along with known duplications of the *KIT* gene, responsible for white coat color in European pigs. Additionally, we identify a recent SINE element insertion in *MYO5A* transcripts of European pigs, potentially influencing alternative splicing patterns and coat color alterations. Furthermore, a Yorkshire-specific copy number gain within *ABCG2* is found, impacting chromatin interactions and gene expression across multiple tissues over a stretch of genomic region of ~200 kb. Preliminary investigations into SV’s impact on gene expression and traits using the Pig Genotype-Tissue Expression (PigGTEx) data reveal SV associations with regulatory variants and gene-trait pairs. For instance, a 51-bp deletion is linked to the lead eQTL of the lipid metabolism regulating gene *FADS3*, whose expression in embryo may affect loin muscle area, as revealed by our transcriptome-wide association studies.

**Conclusions:**

This SV catalog serves as a valuable resource for studying diversity, evolutionary history, and functional shaping of the pig genome by processes like domestication, trait-based breeding, and adaptive evolution.

**Supplementary Information:**

The online version contains supplementary material available at 10.1186/s13059-024-03253-3.

## Background

Pigs not only serve as a major human nutrition source but also as biomedical models for studying human diseases and potential xenotransplant organ donors [1, 2]. The genetic improvement of economically important complex traits in pigs such as growth, feed efficiency, and health led to the efficient and sustainable production of animal protein, contributing to a secure food supply for a growing world population. Because pigs are one of the most important livestock animals and have more anatomical and physiological similarities to humans, their overall importance calls for a thorough exploration of their genomics and functional genomics.

The Eurasian wild boar originated from Southeast Asia, diverging from other species in the genus *Sus* ~3–6 Mya [3]. They then diverged into Eastern and Western clades ~1.2 Mya over the Eurasian mainland, with the European wild boar being much less variable [4]. Modern pigs (*Sus scrofa*) were domesticated from these independent wild boar populations in Asia and Europe about 10,000 years ago with geographical isolation [5, 6]. Subsequent intensification of pig breeding has led to distinct pig breeds with various phenotypic characteristics [6, 7]. Western breeds have been subjected to intense selection for major economic traits, including higher growth rates, muscle mass, and feed efficiency. In contrast, Asian breeds like Meishan pigs possess a stronger tolerance to roughage and high intramuscular fat [8]. During the Industrial Revolution, Asian pigs were imported to Europe to improve local breeds, such as the Large White and Duroc breeds, for key traits like fertility, growth, and fatness [7, 9]. Because of these events, pigs have become an excellent model for studying divergence and subsequent hybridization between populations during the last 1.2 million years. Despite hybridization and introgression, substantial differences remain between modern Asian and European pigs. Population analyses based on SNP, short insertion and deletion, and microsatellite markers suggest that pigs can be divided into several major distinct genetic groups, including Asian and European breeds [10].

The discovery of structural variation (SV) revolutionized the understanding of the genomic landscape in many species [11]. This form of variation involves a larger proportion of the genome than SNP, short insertion/deletion (InDel), and microsatellites. SV can take the form of deletions, insertions, and duplications (commonly grouped under the term copy number variation or CNV), as well as inversions and translocations, which have been defined as ranging from 50 base pairs (bp) up to 5 megabase pairs (Mb) [12]. With the increasing size of SVs, the likelihood of impacting genes and their expression increases, such as altering the sequence, splicing, or copy number of a gene or changing the position or composition of *cis*-regulatory sequences [13–16].

Previous studies have demonstrated that SV is present in the pig genomes [4, 17–19] and found associations between SV and phenotype [20–23]. One example of an SV affecting pig coat color is that four duplications at the *KIT* gene are exclusively present in white or white-spotted pigs, carrying the *Dominant white*, *Patch*, or *Belt* alleles [24]. We also reported many SVs between the Meishan and Duroc pigs by comparing the de novo assemblies of MSCAAS v1 to Sscrofa11.1 in our previous work [25]. Swine SV has been identified often by mapping reads from various breeds to the Duroc reference genome, although detection can be more complicated than SNP. For instance, complex SVs can be located in or near repetitive sequences like mobile element insertions (MEI), which could interfere with accurate read mapping and introduce ambiguity in breakpoint definition [26–28]. Multiple solutions to this problem have been applied, including read pair (RP) or paired end mapping (PEM) and read depth (RD) or split read (SR) analysis [29]. The success of each algorithm depends on the SV type and size, and all are sensitive to the quality of the reference genome and other factors. For example, the RD approach is the most used and has successfully identified SV using short reads in pigs previously [30, 31], but it has lower accuracy in defining SV boundaries. In addition, the use of a single strategy could introduce a high proportion of false positives, while combining different strategies can significantly increase the sensitivity and specificity of SV detection [32]. Furthermore, previous works were limited to both sample size and breed representation that cannot accurately and effectively evaluate the extent and abundance of SVs in the global pig populations. Finally, the lack of functional annotation and GWAS results from a wide range of complex traits in the previous studies also restricted our understanding of how SV affects the regulatory landscape of the genome and, eventually, complex traits of economic value in pigs.

Here, we applied a combination of multiple approaches to build a comprehensive SV map across the pig genome by using 1060 genomes representing 101 pig breeds sampled worldwide. The pipeline identified millions of SV events, which were used to assemble an enhanced SV catalog. We reconstructed breed ancestry and crossbreeding processes using SV among European and Asian pig breeds. We then conducted an initial exploration into how SVs might impact gene expression, functional elements, and complex traits of economic significance. As described previously [33], this was performed by examining their linkage disequilibrium with other functional variants produced by the Pig Genotype-Tissue Expression (PigGTEx) project. As part of the FarmGTEx project, the PigGTEx generated a comprehensive catalog of expression quantitative trait loci (eQTLs) and splicing quantitative trait loci (sQTLs) in 34 pig tissues, as well as conducted sequence-based genome-wide association studies (GWAS) and gene-based transcriptome-wide association studies (TWAS) for many complex traits in pigs [34, 35]. Therefore, this SV catalog represents a valuable resource for studying diversity and evolutionary history in pigs and how domestication, trait-based breeding, and adaptive evolution have functionally shaped the pig genome.

## Results

### SV contents of worldwide pig populations

**Fig. 1:**
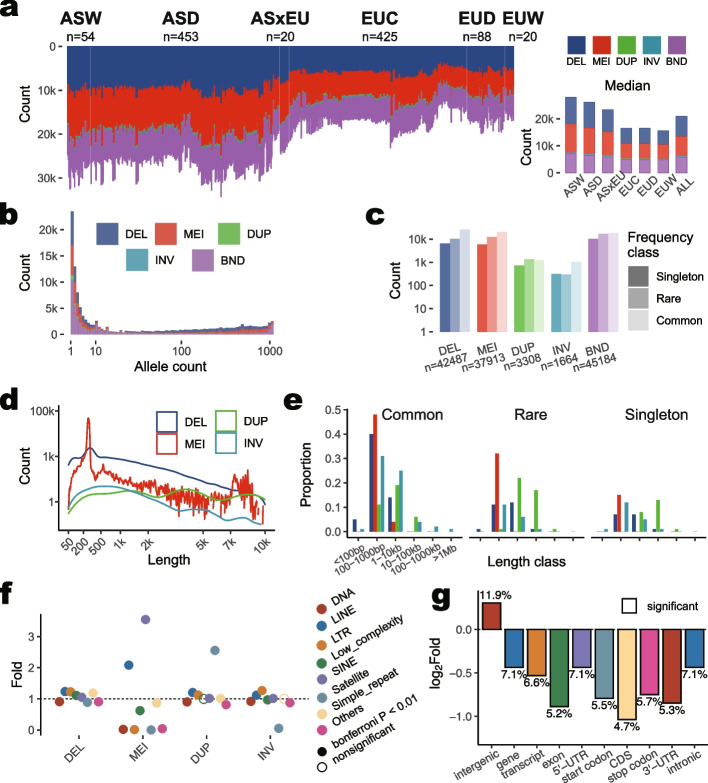
SV distributions across individuals, populations, and genomes of 1060 pigs. **a** Number of SV across 1060 pigs. The *k* in the y-axis denotes one thousand. Counts for five SV types in individuals are shown on the left. The lower right panel refers to the median SV count for individuals in each population. Five SV types are deletion (DEL), mobile-element insertion (MEI), duplication (DUP), inversion (INV), and breakend (BND). **b** SV frequency distribution. The *x*-axis (allele count) is transformed by log 10. **c** SV counts in each frequency class for each SV type. Singleton, rare, and common respectively denote SV only found in one individual, found in more than one individual but not exceeding 1% of individuals, and found in more than 1% of individuals. The y-axis is transformed by log 10. **d** Size distributions of SVs. The *x*-axis is transformed by square root and y-axis by log 10. **e** Proportion distributions of length for each SV frequency class. It included ≤ 100 bp, 100–1000 bp, 1–10 kb, 10–100 kb, 100–1000 kb, and ≥ 1 Mb. The color legend is shown in** d**. **f** Enrichments of repeats in SV against the whole genome. Folds were calculated by the proportion of repeat length in SV divided by the proportion of total repeat length in the genome. Significance was determined by the Chi-squared Test and adjusted by the Bonferroni methods with threshold adjust *P* ≤ 0.01. Filled circle: significant; open circle: nonsignificant. **g** Enrichments of protein-coding genes in SV to the genome. The numbers above or underneath bars denote the value of fold changes. All bars boxed in solid black are significant

We started from a total of 1208 short-read whole-genome sequence (WGS) datasets with ≥ 10× collected for the PigGTEx project, of which 90.7% were kept after filtering, encompassing 1060 pigs (*Sus scrofa*) and 36 outgroup individuals (See Methods). For deep-sequenced samples, we randomly downsampled their reads to 15×. The workflow of this project is shown in Additional file 1: Fig. S1. Based on the ancestry composition and geographical locations, we divided them into 7 main populations and 112 sub-populations (101 for pigs). Using an integrated SV calling pipeline, we discovered and genotyped a total of 130,556 nonredundant high-quality SVs across 1060 pigs. For each individual pig, the SV count ranged from 11,578 to 34,302, with a median count of 20,594. These nonredundant events included 42,487 deletion (DEL), 37,913 mobile-element insertion (MEI), 3308 duplication (DUP), 1664 inversion (INV), and 45,184 breakend (BND) events (Fig. 1a). The sum of the lengths of DEL, MEI, DUP, and INV were 96.3, 16.6, 61.3, and 59.6 Mb, respectively, constituting 9.6% (233.8 Mb) of the *Sus scrofa* genome (Table 1, Additional file 2: Tables S1, S2, and S3). To validate the SVs called from short reads (i.e., WGS SVs) using our current pipeline, we collected SVs called from the comparison of PacBio long-read-based assemblies, the Meishan MSCAASv1 [25] *vs*. Duroc Sscrofa11.1 [36], using the mummer program. In the 30 Meishan pigs analyzed, 51.70% (by length) and 44.65% (by count) of the WGS SVs could be successfully validated (Additional file 2: Tables S4 and S5). For the overlapped SVs, 83.9% of WGS SVs from WGS Meishan pigs and 81.0% of SVs from the comparison of long-read assemblies had more than 98% length consistency.

**Table 1:** Nonredundant SV statistics for main populations

**Main-population**	**Sub-population count**	**Sample count**	**Count**							**Length(Mb)**					**Percentage of genome**			
			**ALL**	**DEL**	**MEI**	**DUP**	**INV**	**BND**	**ALL**	**DEL**	**MEI**	**DUP**	**INV**	**ALL**	**DEL**	**MEI**	**DUP**	**INV**
**ALL(NoOTG)**	101	1060	130,556	42,487	37,913	3308	1664	45,184	233.83	96.32	16.60	61.29	59.58	9.60%	3.96%	0.68%	2.52%	2.45%
**ASW**	17	54	63,802	26,279	19,371	920	962	16,270	87.08	26.84	10.84	8.48	40.91	3.58%	1.10%	0.45%	0.35%	1.68%
**ASD**	48	453	91,235	34,271	25,880	2540	1337	27,207	177.29	72.01	13.09	45.31	46.86	7.28%	2.96%	0.54%	1.86%	1.92%
**ASxEU**	1	20	46,566	18,283	16,589	583	655	10,456	66.60	15.02	9.56	4.45	37.56	2.73%	0.62%	0.39%	0.18%	1.54%
**EUC**	5	425	95,379	24,964	32,808	1443	962	35,202	98.60	25.51	14.12	14.25	44.68	4.05%	1.05%	0.58%	0.59%	1.83%
**EUD**	21	88	58,521	21,477	18,510	1225	787	16,522	85.22	24.34	9.69	11.71	39.46	3.50%	1.00%	0.40%	0.48%	1.62%
**EUW**	9	20	33,637	12,515	10,927	481	427	9287	54.43	11.80	6.80	4.83	30.99	2.23%	0.48%	0.28%	0.20%	1.27%
**OTG**	11	36	164,806	56,184	48,412	798	4414	54,998	274.21	73.33	25.95	32.30	142.58	11.26%	3.01%	1.07%	1.33%	5.85%

We detected median counts of 26,458 and 16,609 SVs in Asian (ASD and ASW) and European pigs (EUC, EUD, and EUW), respectively (Fig. 1a), suggesting a possible bias for SV discovery using the current pig reference assembly (Duroc Sscrofa11.1) as it was derived from the Duroc breed. The 164,806 SVs detected from 36 outgroup individuals covered 11.3% of the pig genome with a total nonredundant length of 274.2 Mb. We observed a lower percentage for singleton SVs (23,497, allele count, AC = 1) as compared to rare SVs (40,916, 1 < AC ≤ 10; i.e., allele frequency, AF < 0.01). A large portion of SVs is common SVs (66,143, AC > 10, AF > 0.01), reflecting a lower diversity in these pig samples which might partially explain lower percentages of singleton SVs and rare SVs in these pig samples (Fig. 1 b and c). The size distribution of SVs showed that the longer events had lower frequencies for DELs, INVs, and MEIs, while longer DUPs held their frequency relatively constant (Fig. 1d and e). MEIs had two peaks at lengths of ~250 bp and ~7500 bp (Fig. 1d), respectively, corresponding to short interspersed nuclear element (SINE) or long interspersed nuclear element (LINE) repeats (Fig. 1f, Additional file 2: Table S6). As expected, LINE and SINE elements were enriched in MEIs. Compared to DELs, DUPs were enriched in satellite regions. In contrast, INVs were underrepresented in satellites.

The distribution of DUP count was asymmetrical as compared with other SV types, in which a larger number (> 300) of DUP events were found in 4 ASD, 4 EUC, and 3 EUD individuals (Additional file 1: Fig. S2a). The total SV lengths and median lengths for EUC (760.1 bp) and EUD (725.3 bp) were generally higher than those of other populations (mean of 668.8 bp), including EUW (Additional file 1: Fig. S2b and c). Of those, the average differences in the median lengths between AS and EU pigs were small for DELs (< 5 bp, 1.3% for the mean of median length) and MEIs (< 1 bp, 0.2%), while those were large for DUPs (> 325 bp, 27.9%) and INVs (> 90 bp, 8.9%). When all SVs were combined, the average counts of homozygous SVs were higher than heterozygous SVs in ASW, ASD, EUD, and EUW, while two counts were either close in EUC or in the opposite direction in ASxEU (Additional file 1: Fig. S2d). The gentle raising accumulative curves for SV allele frequency were observed in ASxEU, ASW, and EUW. At the same time, EUC pigs had the steepest accumulative curve indicating they have low biodiversity, which may be due to the high selection strength during industrial breeding (Additional file 1: Fig. S2e).

### SV colocalization with genes, chromatin states, regulatory variants, and complex trait QTLs

**Fig. 2:**
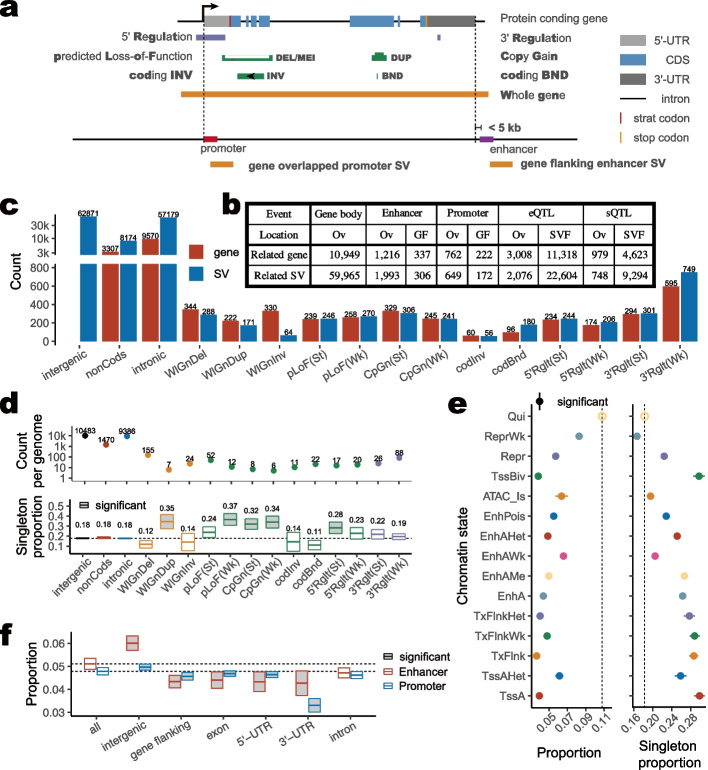
SV-related gene, regulator, and e/sQTL. **a** Categories of gene-overlapping SVs. The top shows gene structure, including transcribe start site (array), 5**′**-UTR (light grey), start codon (red), coding sequence (blue), stop codon (yellow), 3**′**-UTR (dark grey), intron and intergenic region (thin black line). A total of 15 categories were defined, including whole gene DEL (WlGnDel), DUP (WlGnDup), and INV (WlGnInv), predicted loss-of-function (pLoF) when DEL or MEI occurred in CDS, copy gain (CpGn) when DUP occurred in CDS, coding INV (codInv), coding BND (codBnd), 5**′** Regulation (5**′**Rglt), and 3**′** Regulation (3**′**Rglt). CDS-mapped SVs were defined as weak (Wk) impact if mapped CDS counts less than 20% of its own length. In contrast, it had a strong (St) impact. Similarly, UTR-mapped SVs were defined as weak (Wk) impacts if mapped UTR lengths were less than 20% of their own lengths. In the bottom part of this panel, we defined the relationships between SVs and genomics features like eQTL, sQTL, promoter, and enhancer as overlapped if they were localized in SVs or flanking if they were localized in 5-kb flanking regions of an SV on both sides. **d** Table of count statistics for SV-related gene, enhancer, promoter, and e/sQTL. Ov denotes overlapped, GF denotes gene flanking, which is 5 kb of each side, and SV F denotes SV flanking. **c** SV and gene counts of each SV category. The upper *y*-axis is transformed by log 10 when more than 3000. **d** Count per genome and singleton proportion for each SV category. Bars represent sample mean, lower and upper Gaussian 95% confidence limits in each individual, based on the t-distribution. The significant difference test for singleton proportion was carried out by the Student’s *t*-test for each category against intergenic. The dotted line shows the mean singleton proportion of intergenic. The Bonferroni-corrected *P* values ≤ 0.01 were considered significant and represented by the bar color fillings of light grey. **e** Count per genome and singleton proportion of each chromatin state. The proportion was calculated by the overlapped length of the chromatin state and SV divided by the length of the chromatin state for each tissue, respectively. The significant difference test for proportion and singleton proportion was by the Student’s *t*-test for each chromatin state against Qui. The dotted line shows the mean of Qui and significant value. The Bonferroni-corrected *P* values ≤ 0.01 were considered significant and shown as filled circles. A total of 15 chromatin states include strongly active promoters/transcripts (TssA), Flanking active TSS without ATAC (TssAHet), transcribed at gene (TxFlnk), weak transcribed at gene (TxFlnkWk), transcribed region without ATAC (TxFlnkHet), strong enhancer (EnhA), medium enhancer with ATAC (EnhAMe), weak active enhancer (EnhAWk), active enhancer no ATAC (EnhAHet), poised enhancer (EnhPois), ATAC island (ATAC_Is), bivalent/poised TSS (TssBiv), repressed polycomb (Repr), weak repressed polycomb (ReprWk), and quiescent (Qui). **f** Ratios of enhancers or promoters in SVs. The *x*-axis denotes the genomic locations of enhancers or promoters. Ratios (the *y*-axis) were calculated by the counts of SV-related enhancers or promoters divided by counts of enhancers or promoters in the genome for each genomic location, respectively. Bars represent the sample mean and lower and upper Gaussian 95% confidence limits of 34 tissues, based on the t-distribution. The significant difference test for ratios was carried out by the Student’s *t*-test for each genomic location to all enhancers and promoters. The dotted lines show mean proportions of enhancer or promoter, respectively

Potential effects of SV on genome function were examined by overlapping tests with distinct genome features, including genic sequence, chromatin states, regulatory variants, and complex trait QTLs in pigs. To investigate the impacts of SV occurring in the genic region, we annotated those 130,566 SVs using gene contents. We found that 45.9% (59,965/130,556) of SVs overlapped with 55.6% (10,949/19,360) of the Ensembl protein-coding genes by count, and 34.5% (80.68 Mb/233.83 Mb) of SVs overlapped with only 7.1% (80.68 Mb/1.14Gb) of genes by length. Those SV-overlapped genes were significantly (Bonferroni adjusted *P* < 0.01) enriched in 23 Gene Ontology (GO) terms, such as phosphorylation (2 top terms; starting with an adjusted P value of 2.38 × 10^−6^), kinase activity, exopeptidase activity, and ion transmembrane transporter activity (Additional file 2: Table S7). Among them, genes overlapped with singleton SVs were related to 5 phosphorylation/kinase terms and 3 anion/chloride channels/transporters (Additional file 2: Table S8). Intergenic regions were 1.41-fold enriched for SVs in the pig genome, significantly higher than intragenic regions. Within the exon regions, coding sequence (CDS) was the most underrepresented partition, with only 3.9% by count and 4.7% by length overlapped SVs (Fig. 1g and Additional file 2: Table S9). In addition, we further broke 59,965 SVs into 15 categories based on their locations in the gene (Additional file 2: Table S10). The 15 category definitions were described in the “Methods” section and shown in Fig. 2a and b. A total of 3078 SVs (2.4%) overlapped with 3080 (15.9%) protein-coding genes, which was much fewer than those SVs in intergenic regions (62,871, 48.2% of SVs), intron (57,179, 43.8% of SVs, and 3307, 17.1% of genes), and noncoding region (8174, 6.3% of SVs). Within exons, the largest number (749, 0.6%) of SVs were found within 3′Rglt(Wk) regions of 595 (0.5%) genes, suggesting that SVs could more likely fine-tune genes’ function by interrupting their 3′-UTR (Fig. 2c). The singleton SV proportion can reflect the diversity of a region in the genome. We found that lower counts per genome and higher singleton proportions of SVs in WlGnDUP, pLoF(Wk), CpGn(St), CpGn(Wk), and 5′Rglt(St) than those in low function regions, indicating SV events in those high functional regions were under evolutionary constraints and severely selected against (Fig. 2d).

We also did colocalization analyses with the 15 chromatin states defined using histone codes, CTCF-seq, and ATAC-seq, as previously reported [37, 38]. The SV-related proportion of length for each state was significantly less than that for the quiescent state (Qui, 10.8%) in 14 tissues (Fig. 2e). The antisymmetric patterns of lower SV-related proportion and higher singleton proportion were found in transcribed at gene (TxFlnk), bivalent/poised transcription start site (TssBiv), active promoters/transcripts (TssA), transcribed region without ATAC (TxFlnkHet), and strong enhancer (EnhA) (Fig. 2e). In contrast, weak repressed polycomb (ReprWk) had the second highest SV-related proportion and the lowest singleton proportion (Fig. 2e, Additional file 2: Tables S11 and S12). Moreover, we further investigated the SVs in active promoters and strong enhancers. Overall, we found that low count percentage for both of them in SV regions, including 5.0% (22,901/438,869) of enhancers and 4.8% (5862/119,892) of promoters after removing the redundancy for all tissues. Of those, the highest proportions were intergenic enhancers and promoters (6.1% and 5.1%, respectively), and the least were in 3′-UTR with 5.2% of enhancers and 4.9% of promoters (Fig. 2f, Additional file 3: Tables S13 and S14).

Quantitative trait locus (QTL) is a genomic region that is associated with a particular phenotypic trait [39]. To explore the potential functional roles of SVs on complex trait variation, we further examined 22,176 QTLs (after filtering for length < 1 Mb) of 565 different pig traits from Animal QTLdb (release 47) [40]. We noticed that 22.3% (4935/22,176) of QTLs for 81.4% (460/565) of traits overlapped with SVs, and 4.6% (26/565) traits of them were significantly enriched (> 2×) in SVs, related to the slaughter, resistance, production, growth, quality, and survival traits in pigs (Additional file 1: Fig. S3a and Additional file 3: Table S15). We also noted that enrichments of SVs with QTLs of 12 traits were different across 6 main pig populations (Bonferroni-adjusted ANOVA *P* < 0.01 and coefficient of variation > 1), including relative area of type IIa fibers, oleic acid to stearic acid ratio, body weight (22 weeks), ovary weight, Interferon-gamma level, meat color L*, mean corpuscular hemoglobin content, average daily gain, backfat at rump, 3–5 h and 9–24 h pH decline, and HDL cholesterol (Additional file 1: Fig. S3b and Additional file 3: Table S16).

### Population structure derived from SVs

**Fig. 3:**
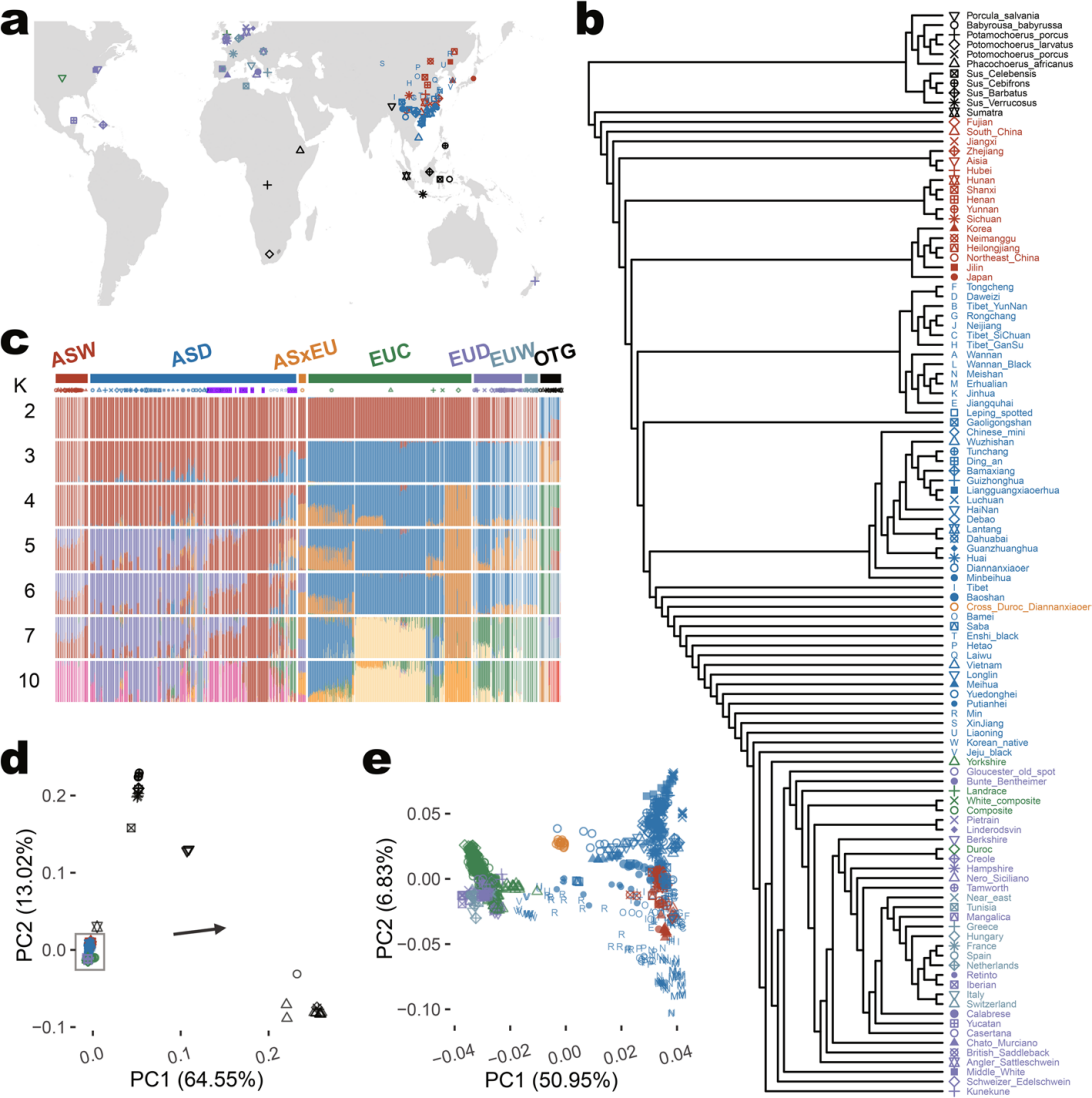
Population structure by SV genotypes in autosomes for 1096 individuals. **a** Pig sample origins around the world. The colors and shapes denote main populations and sub-populations, as shown in panel **b**. **b** Maximum likelihood tree for 111 sub-populations was inferred by TreeMix based on allele frequency for each autosomal SV genotype and plotted by R package ggtree. **c** Admixture analysis of pigs from 7 main populations by fastStructure. *K* denotes the assumed number of ancestors. **b** Principal component analysis (PCA) for 1060 pigs and 36 outgroup individuals. Explained variation percentages in parentheses for PC1 and PC2 were calculated by PLINK. **e** PCA for 1060 pigs. Explained variation percentages in parentheses for PC1 and PC2 were calculated by PLINK

To further explore SV characteristics across global pig populations, we carried out population genetic analyses. We plotted the geographical locations for the ancestral group origins of those 1060 pigs and 36 outgroup individuals in Fig. 3a. After clustering based on 201,589 SV genotypes using a maximum likelihood method for all 112 sub-populations from 7 main populations, we obtained a phylogenetic tree that grouped individuals into their ancestral groups clearly (Fig. 3b). The admixture analysis shows that EUC pigs were well-separated according to their specific breeds. For example, Duroc and Yorkshire had their own specific clusters (Fig. 3c). But Landrace and the Composite (AS×EU) were mixed when *K* = 5. In contrast, most AS pigs were mixtures of two or more distinct presumed ancestors, except for two Chinese native breeds—Erhualian and Meishan (K = 7). When *K* = 10, the Chinese mini pig clustered by itself (Fig. 3c). Taking together, our admixture analysis results demonstrated, for the first time in pigs, that SV genotypes produce a similar population structure and ancestral components as SNP genotypes did, as published before [10], providing validation that our SV calling pipeline correctly identified and genotyped these SVs.

To further globally evaluate the genotype accuracy of SVs, we used individual SV calls as alleles of genetic markers to analyze population structure and compare the predicted structure to that inferred from SNP markers (Fig. 3d–e). The principal component analysis (PCA) of SVs successfully distinguished individuals from different geographical regions (Fig. 3d–e) and mirrored the distributions predicted from SNPs [7, 10]. For PCA with 36 outgroup individuals, we found the distributions of different pig breeds were tightly squeezed together into points on the lower left (Fig. 3d). After removing 36 outgroup individuals (Fig. 3e), the first PC divided European and Asian pigs into two main groups, with ASxEU in the middle between them, and the second PC separated European pigs (Fig. 3e). Jeju black, Korean native, Liaoning, Min pigs were close to Yorkshire. Duroc is the most distinct breed in EUC, far away from AS pigs. Also, Luchuan, Liangguangxiaoerhua, Erhualian, and Meishan breeds in AS pigs were more divergent from EU pigs (Fig. 3b and c).

### Selection signatures of SVs among different pig populations

**Fig. 4:**
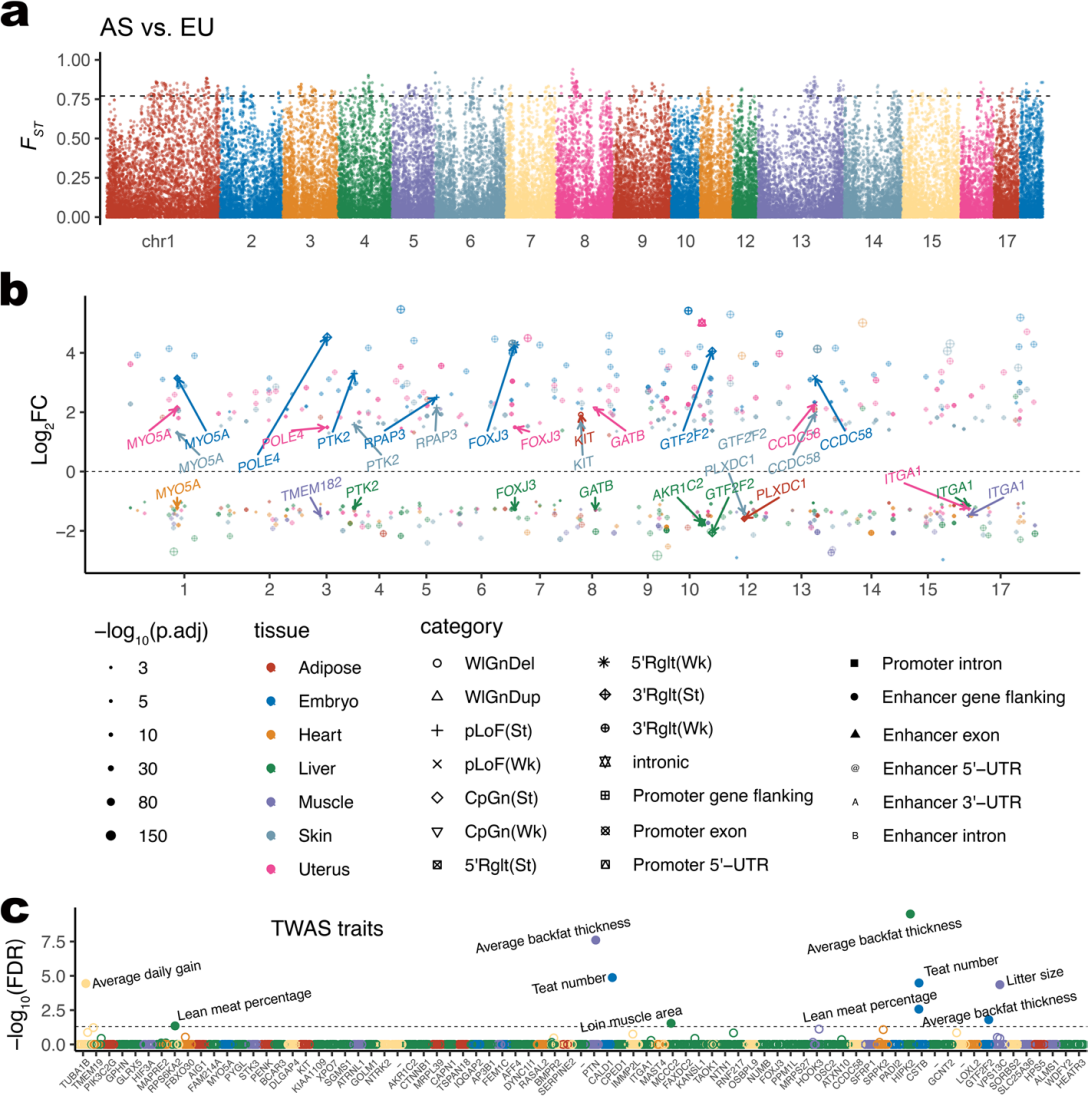
Pairwise comparisons of SV-related gene expressions between AS and EU ancestral pigs. **a** Manhattan plot of *F*_*ST*_ value between AS and EU ancestral pigs based on autosomal SVs. *F*_*ST*_ values were calculated by PLINK. The dotted line at 0.754 shows the threshold for the top 1%* F*_*ST*_ values of 130,556 SVs. **b** Fold changes of SV-related DEGs between AS and EU ancestral pigs. The significant difference test was based on the exactTest in R package edgeR. Common (AF ≥ 0.01) DEL (including ref-MEI) and DUP-related DEGs in at least two tissues were labeled out with their gene symbols. **c** SV-related DEG associated with pig complex traits integrating by TWAS data. The *x*-axis denotes each SV-related DEG analyzed in TWAS. The *y*-axis denotes the false discovery rate (FDR) of each TWAS gene, and the threshold of FDR ≤ 0.05 is shown by the dotted line

SV genotypes were also used to identify signatures of selection between populations, beginning with an estimation of fixation index (*F*_*ST*_) statistics to determine deletion frequency differences between AS and EU pigs. We calculated SV-based *F*_*ST*_ and identified lineage-differential SVs as those with the highest *F*_*ST*_ (top 1%) between AS and EU ancestral groups, main populations, and sub-populations (see the “Methods” section, Fig. 4a and Additional file 1: Fig. S4ab).

There were 571 SVs with a nonredundant length of 221.2 kb and overlapping with 216 genes, corresponding to the top 1% of *F*_*ST*_ values, which implies potential selection signatures between AS and EU ancestral groups (Additional file 3: Tables S17 and S18). For those in population-specific SVs, we found 27,144 AS-specific SVs with a nonredundant length of 102.22 Mb. Of those, 10,081 were common SV (AF ≥ 0.01 in the group) with a length of 23.79 Mb overlapped with 2531 genes that were significantly related to the KEGG pathway for axon guidance, while 5240 EU-specific common SV with 5.32 Mb (total 33,351 SVs with 39.82 Mb) related to 1766 genes enriched in amyotrophic lateral sclerosis pathway, and catalytic complex, intracellular protein-containing complex, RNA polymerase II, holoenzyme, transferase complex (Additional file 1: Fig. S4c, d and Additional file 3: Tables S19 and S20). The 509 SVs with the highest *F*_*ST*_ between Meishan and Pietrain with 196.8 kb length overlapped 204 genes, which were significantly enriched in adult behavior.

### SV-related differentially expressed genes (DEGs) between AS and EU ancestral pigs

In the above section, we detected 281 SVs with the highest *F*_*ST*_ between AS and EU ancestral groups, along with 3031 AS-specific SVs and 1104 EU-specific SVs, which overlapped with protein-coding gene (either directly overlapped with the gene body or overlapped with enhancer/promoter regions ranging from 5 kb upstream to 5 kb downstream of the gene body), after removing low-frequency SVs (AF < 0.05). They covered 98.3 kb, 6.17 Mb, 1.47 Mb genomic regions and were annotated with 217, 1872 (24 flanking), and 733 (4 flanking) genes, respectively (Additional file 3: Tables S21, S22, and S23). Based on the 1171 public RNA-seq data from 12 tissues in 8 sub-populations, including Meishan, Erhualian, Wuzhishan, Bamaxiang, Yorkshire, Duroc, Composite, and Pietrain, we performed DEG analyses in each tissue between both AS and EU groups with at least two sub-populations for each group (see Methods for details). A total of 17.5% (811/4643) DEGs mapped to 1478 SVs were detected (Fig. 4b and Table S24).

In addition, 35.1% (285/811) of SV-related DEGs, representing 38.6% (570/1478) SVs, were multiple-tissue DEGs (≥ 2), including 45.7% (32/70), 35.4% (219/618), and 34.3% (79/230) genes mapped to the highest *F*_*ST*_, AS-specific, and EU-specific SV, respectively (Additional file 3: Table S25). The most prevalent SV types of multiple-tissue DEGs between AS and EU ancestral groups were intronic DELs (or ref-MEI, 216/285, 75.8%). There were 4 and 1 multi-tissue DEG with SVs in their enhancer and promoter regions, respectively (Additional file 3: Table S26). One EU-specific whole gene DUP (*8:41223207–41783659*) was also detected in this analysis covering the *KIT* gene, whose expression was significantly higher in the skin and adipose of EU pigs than those of AS breeds. The *KIT* gene with high expression facilitates the white coat color phenotype, which has been well investigated previously (Additional file 1: Fig. S5) [24, 41]. Of those 286 SV-related multiple-tissue DEGs, 51.0% (146/286) genes related with 50.3% (287/571) SVs had the discordant expression levels (up- or down-regulation) between tissues (Additional file 3: Table S26). We annotated those SV-related multiple-tissue DEGs using 14 TWAS traits of 34 tissues and found 9 of them, including *RPS6KA2*, *PTN*, *CALD1*, *HIPK2*, *GTF2F2*, *VPS13C*, *CSTB*, *TUBA1B*, and *MCCC2* whose expression levels in a given tissue were genetically associated with pig lean meat percentage, average backfat thickness, teat number, litter size, average daily gain, and loin muscle area, respectively (Fig. 4c and Additional file 3: Table S27).

We showed 5 DEL-related, 3 ref-MEI-related, and 4 DUP-related multiple-tissue DEGs (Table 2). There was the highest *F*_*ST*_ DEL-related gene *MYO5A*, whose expression was significantly different between AS and EU pigs in the embryo (log_2_FC = 3.15), skin (1.31), uterus (2.13), and heart (-1.22). It is noted that the expression pattern of *MYO5A* in AS and EU pigs’ heart tissue was different from those of the other three tissues, indicating the potential SV regulation mechanisms of gene expression tend to be tissue-specific. Nine AS-specific SV-related genes were *AKR1C2* (related to age of puberty and possibly ovulation rate) [42], *GTF2F2* (feed efficiency) [43], *PLXDC1* (response to infection with a highly pathogenic strain of porcine reproductive and respiratory syndrome virus) [44], *CCDC58* (energy metabolism) [45], *ITGA1* (related to muscle development, glycogen metabolism and mitochondrial dynamics of piglet, marbling development of Korean Hanwoo breed) [46, 47], *POLE4* (venous thromboembolism) [48], *PTK2* (milk production traits in Chinese Holstein) [49], *FOXJ3* (abnormality of refraction) [50], and *GATB* (piglet mortality) [51]. Finally, two EU-specific SV-related genes were *KIT* (as mentioned above) and *RPAP3* (related to strong selection signatures, immune system, food efficiency, and low nutritional requirement) [52, 53].

**Table 2:** SV-related DEGs in AS and EU ancestral group pairwise comparison

**SV**	**Specific or high *F***_***ST***_	**Gene name**	**Event type**	**Tssue**	**logFC**	**Bonferroni *P***
1:119154722–119155024:DEL	Highest *F*_*ST*_	*MYO5A*	3′-UTR DEL (MEI)	Embryo	3.15	4.66E−12
				Heart	− 1.22	3.44E−03
				Skin	1.31	3.29E−03
				Uterus	2.13	1.13E−08
10:65581173–65581438:MEI	AS specific	*AKR1C2*	5′-UTR DEL (MEI)	Liver	− 1.72	4.38E−18
			pLoF (St)			
			Promoter DEL (MEI)			
			5′-UTR DEL (MEI)	Uterus	5.01	8.93E−27
			pLoF (St)			
11:22010860–22011147:MEI	AS specific	*GTF2F2*	3′-UTR DEL (MEI)	Embryo	4.06	3.25E−21
				Liver	− 2.06	1.09E−20
				Skin	1.38	1.35E−03
12:23080412–23096524:DUP	AS specific	*PLXDC1*	CpGn (Wk)	Adipose	− 1.59	1.88E−11
			Enhancer DUP			
			CpGn (Wk)	Skin	− 1.41	3.67E−04
13:138175850–138229580:DUP	AS specific	*CCDC58*	CpGn (St)	Embryo	3.17	2.70E−12
				Skin	1.96	9.79E−09
				Uterus	2.28	1.63E−08
16:32285866–32287949:DEL	AS specific	*ITGA1*	pLoF (Wk)	Liver	− 1.15	7.65E−05
				Muscle	− 1.48	3.27E−04
				Uterus	−1.26	1.58E−03
3:68099167–68099419:MEI	AS specific	*POLE4*	3′-UTR DEL (MEI)	Embryo	4.53	3.71E−26
				Uterus	1.49	3.13E−03
4:2689951–2695878:DEL	AS specific	*PTK2*	pLoF (Wk)	Embryo	3.30	1.75E−11
			Enhancer DEL (MEI)	Liver	− 1.22	2.11E−04
			pLoF (Wk)			
			pLoF (Wk)	Skin	1.58	2.11E−05
6:169060140–169064984:DEL	AS specific	*FOXJ3*	3′-UTR DEL (MEI)	Embryo	4.27	5.39E−24
				Liver	− 1.27	1.72E−03
				Uterus	1.49	3.31E−03
8:77217828–77233717:DUP	AS specific	*GATB*	5′-UTR DUP	Liver	− 1.30	8.83E−03
			Enhancer DUP			
			5′-UTR DUP	Uterus	2.13	1.67E−05
5:77968631–77972413:DEL	EU specific	*RPAP3*	pLoF (Wk)	Embryo	2.50	1.99E−06
				Skin	2.15	8.93E−10
8:41223207–41783659:DUP	EU specific	*KIT*	eQTL DUP	Adipose	1.92	4.53E−12
			WlGnDup			
				Skin	1.68	1.40E−06

### Examples of SV-related DEGs between AS and EU ancestral pigs

**Fig. 5:**
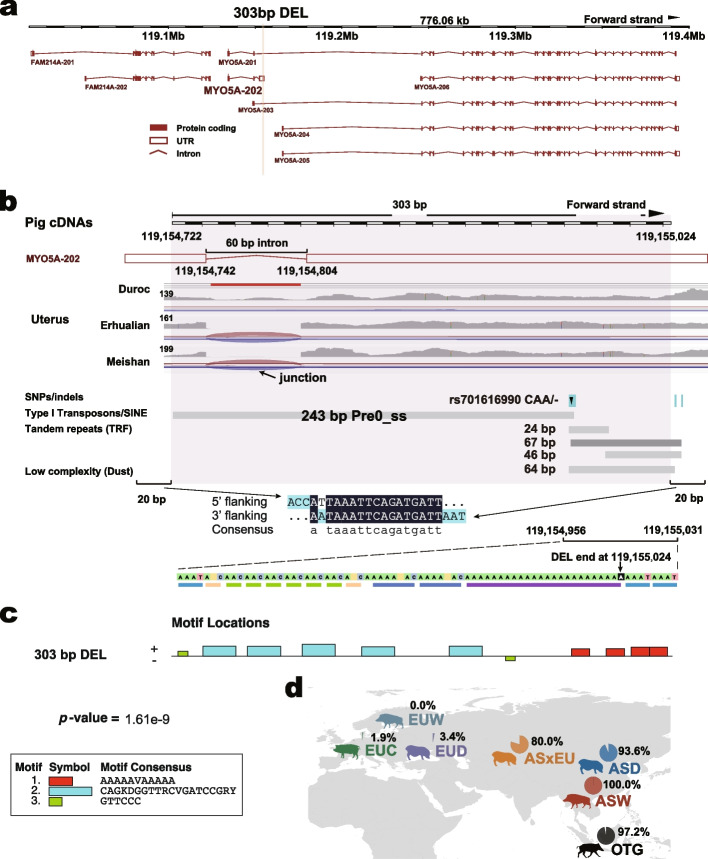
The 303 bp DEL in a 3**′**-UTR of gene *MYO5A*.** a** Genome locations of the 303 bp DEL. Plots were based on the *Sus scrofa* genome annotation of the ENSEMBL database. The yellow box denotes the 303 bp DEL occurred location. **b** Illustration of the genomic region for the 303 bp DEL. Screenshot of integrative genomics viewer for the 303 bp DEL. The top black and white bar shows the location of the 303 bp DEL for RNA or cDNA in the uterus of Duroc, Erhualian, and Meishan pigs. The red and blue lines show forward and reverse strand junction reads, respectively. The upper junction reads for each sample were read peaks. The DEL was located at the 3**′**-UTR of transcript MYO5A-202, across an intron and two exons, which consist of a 243-bp SINE/Pre0_ss transposon, a 67-bp A-rich low complexity sequence. All annotations are from the ENSEMBL database. The middle sequence shows the alignment for flanking 20 bp of DEL two ends. The lower color boxed sequences and different color blocks show the low complexity sequence characteristic for the 3**′** tail of DEL. **c** The 303 bp DEL related 3 motifs. The MEME online tool (https://meme-suite.org/meme/) was used to search and enrich related gene ontology terms of motifs in the sequence of the 303-bp DEL in the *MYO5A* gene. **d** Allele frequency of this 303-bp DEL for each main population

We further checked the 303 bp DEL (1:119154722–119155024) occurred in the second intron of a transcript of gene *MYO5A* (MYO5A-201) (Table 2), and there were many reads flanking it from RNA-seq in AS pigs (Fig. 5a–b). This DEL in AS corresponded perfectly with a young pig SINE/Pre0_ss of 243 bp and a ~64-bp tandem repeat ploy-A tail, which were flanked by a signature of target site duplication of 15-bp sequence within their 20-bp flanking regions. The observed allele frequency of DEL was near 0 % for EUW pigs and near 100% for ASW pigs, and this was true for most EUD and EUC pigs, which did not have this DEL as compared to ASD pigs (Fig. 5d and Additional file 3: Table S28). As the selection of a reference genome (a European Duroc pig) can affect the interpretation of SV results, MEIs in the reference genome were frequently identified as deletions in studied animals lacking such MEIs. Also, because it is almost impossible to remove inserted MEIs from the genomes without leaving any mark, these discovered deletions should be classified as MEIs in the European pigs instead. We thus hypothesize that this SINE/Pre0 MEI was specifically inserted into EU breeds recently. We first screened this SINE/Pre0 MEI and found three candidate motif types that may mediate the transcription factor binding (Fig. 5). We then reassembled the *MYO5A* gene transcripts using StringTie (v2.1.7). Wuzhishan and Bamaxiang pigs with diluted (dark patch on pale skin) color phenotype made up the samples for the AS group, while Yorkshire and Composite pigs with white color phenotype made up the samples for EU pigs. A total of 20 transcripts were obtained as shown in Additional file 1: Fig. S6a, including 6 known transcripts (MYO5A-201 to 206) and 14 novel and predicted transcripts (beginning with MSTRG.8280). We did not detect significant expressions in skin for 3 of them (MSTRG.8280.4, MSTRG.8280.7, and MSTRG.8280.10). For the rest, we found AS (Wuzhishan and Bamaxiang) pigs had at least 2 times higher expression than EU (Yorkshire and Composite) pigs for 16 transcripts (Additional file 1: Fig. S6ab), such as for transcripts MYO5A-204, MYO5A-205, MSTRG.8280.2, MSTRG.8280.3, MSTRG.8280.11, and MSTRG.8280.12. The only exception is MYO5A-201, for which expression in EU pigs was significantly higher than in AS pigs (FC = 2.15, adjusted *p*-value ≤ 0.05). It is remarkable to note that this 303 bp MEI/DEL overlaps with alternative splicing for MYO5A-202, as shown in Fig. 5b. We speculated that similar overlapping thus alternative splicing situations may occur for MSTRG.8280.1, 2, 3, 4, 6, and 7. Especially for MSTRG.8280.3, As pigs had significantly higher absolute transcript levels and ratios, as compared to EU pigs (Additional file 1: Fig. S6ab). All these results made us speculate the 303 bp MEI/DEL might influence the MYO5A transcript splicing and expression, thus resulting in the coat color difference among these four pig breeds. For example, it is possible that this new MEI brought in a new untranslated region, which broke MYO5A-201 into the two disconnected regions, leading to the creation of two short transcripts of MYO5A-202 and MYO5A-206 (Fig. 5a). The changes in splicing patterns and expression level of the *MYO5A* transcripts may be related to altered protein level and melanosome transport, suggesting a potential mechanistic link between the MEI and the coat color phenotype. Future experiments will be needed to validate this hypothesis.

### SV-related DEGs among sub-populations

To investigate SV-related DEGs among sub-populations, we selected three EU breeds and four AS breeds, which were good representations and had large sample sizes of WGS datasets, including Yorkshire (*n* = 187), Duroc (70), Pietrain (33), Meishan (30), Erhualian (24), and Wuzhishan (18). Landraces were removed due to the lack of RNA-seq data. A total of 1085 samples for 14 tissues from those 7 breeds were used in this analysis (Additional file 3: Table S29). Similar to ancestral group comparison, we paired every breed with each of the other six breeds, focusing on breed-specific SVs or breed-differential SVs with the top 1% *F*_*ST*_ values.

We detected 2246 SVs and 1286 SV-related DEGs (Additional file 3: Table S30). Of those, 20.8% (267/1286) genes (related with 20.5% for SV, 461/2246) were differently expressed in at least two tissues, and 20.7% (266/1286) DEGs (related with 19.5% for SV, 437/2246) in at least two sub-populations. There were 113 (8.8%, 113/1286) DEGs, related to 8.4% (189/2246) of SVs, both in at least two tissues and two breeds.

**Fig. 6:**
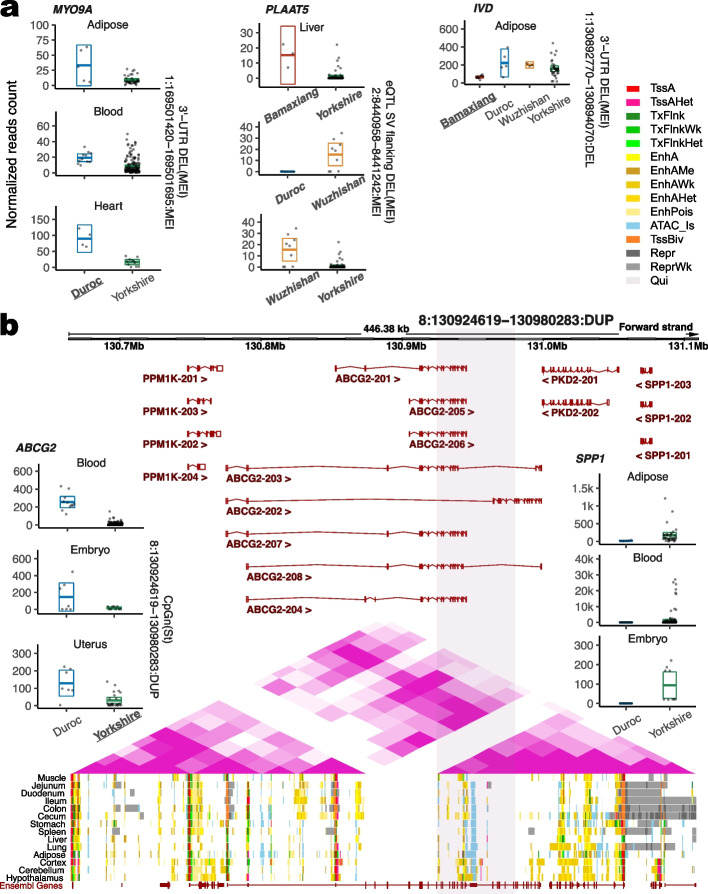
SV-related DEGs among sub-populations. **a** Normalized reads counts for three gene examples. For the crossbar plot, bars represent the sample mean, and lower and upper Gaussian 95% confidence limits of each individual, based on the t-distribution. Small points show gene expression for each individual. Bold underline texts of breeds refer to the specific SV occurred breeds. Bold italic texts refer to the highest *F*_*ST*_ SV paired breed. **b** Illustration of the genomic region for copy number gain of *ABCG2*. 8:130924619−130980283:DUP is shown by the pink box. Gene expression for three genes is shown on two sides of the figure. Hi-C TADs are annotated by purple triangles. Fifteen chromatin states of 14 tissues for Yorkshire are shown in different colors. Screenshot of integrative genomics viewer including read peaks and alignments for embryo RNA-seq data in Duroc and Yorkshire at the bottom

Three examples are shown in Fig. 6a. The first one is *MYO9A*, distinct from the previously mentioned *MYO5A. MYO9A*’s 3′-UTR is interrupted by a Duroc-specific SINE/Pre0_SS element (1:169501420–169501695:MEI), which may be linked to the ~4× increased *MYO9A* expression in adipose (log_2_FC = 1.97), blood (2.00), and heart (2.48), when we compared Duroc with Yorkshire. Another SV with the highest *F*_*ST*_, covering a SINE/Pre0_SS element (2:8440958-8441242:MEI), seems to be under selection in three breed pair comparisons, including Bamaxiang vs. Yorkshire, Wuzhishan vs. Duroc, and Wuzhishan vs. Yorkshire. It was found to be associated with higher *PLAAT5* expression in the liver in AS breeds compared to EU breeds [34]. Finally, Bamaxiang-specific DEL (1:130892770–130894070:DEL) occurs in a 3′-UTRs of *IVD* transcripts, which correlated with the lower *IVD* expression in Bamaxiang than those in Duroc, Yorkshire, and Wuzhishan.

We detected a copy number gain (Yorkshire-specific DUP of 55,665 bp, 8:130924619–130980283) within *ABCG2*, which could potentially downregulate its expression in blood, embryo, and uterus in Yorkshire as compared to Duroc. We further illuminated this region with related genomic features, chromatin states of 14 tissues from two Yorkshire males, and Hi-C interactions in the liver from Yorkshire (Fig. 6b). Interestingly, we found that a large topologically associating domain (TAD) was precisely interrupted by the upstream breakpoints of this DUP in the *ABCG2* gene body, resulting in two similar TADs with half size. In the first small TAD, we observed strong chromatin interactions between the genes *PPM1K* at 3047 bp upstream and *ABCG2*, but no expression difference between Duroc and Yorkshire in 9 tissues (Additional file 1: Fig. S7, Bonferroni corrected *P* ≥ 0.01 or filtered). On the other hand, we found a breed-specific upregulation of a remote gene in Yorkshire potentially mediated by the interactions in the second TAD induced by the DUP. Specifically, this DUP did not seem to affect the expression of its neighbor gene *PKD2* (7011 bp downstream of *ABCG2*) but led to the increased expression of *SPP1* (79,547 bp downstream of *ABCG2*) in adipose, embryo, and uterus in Yorkshire when compared to Duroc (Additional file 1: Fig. S7). Also, we noted that no chromatin state was assigned in front of this DUP, as indicated by the blank regions (Fig. 6b). ATAC islands and strong enhancers (EnhA) of 14 tissues were covered by this DUP, while their patterns were different, such as adipose had the largest region (9205 bp in light blue) of ATAC islands and muscle had the shortest (801 bp). Nine tissues, including hypothalamus (2205 bp), cortex (1804 bp), cecum (1604 bp), muscle (1004 bp), cerebellum (1003 bp), colon (1002 bp), ileum (802 bp), lung (801 bp), and jejunum (602 bp) had strong enhancers (EnhA) but not for the other five tissues (adipose, duodenum, liver, spleen, and stomach) in Yorkshire pigs (Additional file 3: Table S31). Therefore, we identified a Yorkshire-specific copy number gain within *ABCG2*, which could rearrange chromatin interactions, downregulate the *ABCG2* gene, but upregulate the downstream gene *SPP1* over an 80-kb distance in multiple tissues.

### Genetic linkage between SV and functional SNP

Due to the lack of long-read WGS and matched RNA-seq data, attempting to directly impute SVs using SNP based on a single haplotype within selected individuals or breeds proved to be challenging, if not impossible. Therefore, evaluating their impacts on RNA-seq or complex traits was difficult. To address this question, we needed to rely on calculations of the linkage disequilibrium (LD) conducted on the population level.

To explore the LD between SV and functional variants within the pig genome, we conducted these analyses on three population levels: 187 Yorkshire pigs, 425 EUC pigs, and all 1060 pigs. We first computed the LD for the combined genotype matrix of SVs and SNPs using PLINK (v1.90b6.21). We reported all pairwise *r*^2^ values if they were larger than 0 within a 1-Mb genomic distance. We observed LD of each SV type with SNPs followed a similar decay pattern over the genomic distance, like SNP-SNP pairs and each SV type with itself (Additional file 1: Fig. S8). Notably, at the 200 kb distance, SNP-SNP pairs exhibited the highest r^2^ values, with a median of ~0.18 for Yorkshire (Additional file 1: Fig. S8a). DEL-DEL pairs displayed LD levels closest to SNP-SNP pairs, producing a median *r*^2^ value of ~0.16 (the top panel of Additional file 1: Fig. S8b), followed by MEI-MEI pairs with a median *r*^2^ value of ~0.10. With expanded sample sizes representing a more complex genetic background, the LD of all pairs displayed a rapid decline. Specifically, in DEL-SNP pairs at 200 kb, the median *r*^2^ values were 0.006 for 425 EUC pigs and 0.002 for 1060 pigs, demonstrating a quick decrease compared to the value of 0.023 observed for 187 Yorkshire pigs (Enlarged panels in Additional file 1: Fig. S8a and Additional file 3: Table S32). Moreover, in 1060 pigs, we identified higher *r*^2^ values of DEL-DEL (median 0.029) and MEI-MEI pairs (0.027) than SNP-SNP pairs (0.014) (the bottom panel of Additional file 1: Fig. S8b), as well as similar *r*^2^ values between DEL-DEL pairs (0.089) and SNP-SNP pairs (0.083) for 425 EUC pigs (the middle panel of Additional file 1: Fig. S8b), in comparison to 187 Yorkshire pigs, as described above and depicted in the top panel of Additional file 1: Fig. S8b. These findings suggest that SVs, particularly DELs or MEIs, exhibit greater stability compared to SNPs in resisting LD breakdown when there is an expanded sample size encompassing a more complex genetic background.

Besides the above general trends, we also summarized the statistics of the LD for all SV-SNP pairs and all SV-functional SNP pairs in this section. We found that more than half (66.45%, 52.96%, and 50.64%) of SVs exhibited LD *r*^2^ ≥ 0.2 (defined as “linked”) with at least one SNP for Yorkshire, EUC and 1060 pigs. Within these three groups, 37.38%, 24.46%, and 22.76% of SVs were found to have their tagged SNPs (*r*^2^ ≥ 0.5), respectively. Additionally, 31.75%, 21.88%, and 19.22% of SVs demonstrated highly tagged linkage (*r*^2^ ≥ 0.8) with their flanking SNPs (Additional file 3: Table S33). While GWAS report significant SNP signals associated with phenotype traits, eQTLs, and sQTLs are genetic variants that modulate gene expression and splicing, respectively [54, 55]. To explore the potential effects of SV on the pig transcriptome and phenotype traits, we then focused on these functional SNPs including leading eQTL and sQTL from 34 pig tissues, as well as significant GWAS signals from 14 pig complex traits (*n* = ~ 60K) in the PigGTEx dataset [34]. In 187 Yorkshire pigs, a total of 5034 (12.49%, 5034/40,290) SVs exhibit linked functional SNPs, including 4097 (10.17%), 2735 (6.79%), and 1783 (4.43%) with eQTLs, sQTLs, and GWAS loci, respectively (Additional file 1: Fig. S9). In 425 EUC pigs, 857 (1.79%), 487(1.02%), and 357 (0.75%) SVs (total unique 1138, 2.38%) were found to be linked with eQTLs, sQTLs, and GWAS loci, respectively. Conversely, among the 1060 pigs, only a small proportion of SVs (87, 0.14%) were linked with 680 functional SNPs (431, 126, and 143 for eQTLs, sQTLs, and GWAS loci, respectively) (Additional file 3: Table S34). These SVs potentially regulate the expression of 241 genes, alter the splicing of 81 transcripts, and impact 11 complex pig traits (Additional file 3: Table S35). Among these, 10 SVs directly overlapped with the introns of nine e/sGenes of their linked e/sQTLs, potentially contributing to the regulation of expression of *CLCA2*, *FDFT1*, *HACD2*, *KISS1*, *NEK3*, *RASSF3*, *SCD5* and the splicing of isoforms of *CLCA2*, *RBFOX1*, *SLC30A8*, *SLC40A1* (Additional file 3: Table S36). Additionally, 19 SVs were linked with more than one functional SNP type, including 10 SVs linked to both eQTL and sQTL loci, 3 SVs linked to both eQTL and GWAS loci, and 6 SVs linked to eQTL, sQTL, and GWAS loci (Additional file 3: Table S35).

**Fig. 7:**
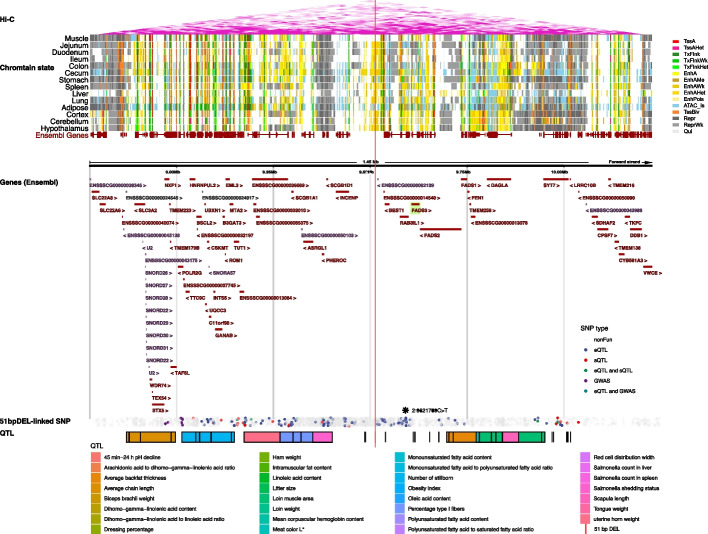
Genomic tracks near the 2 Mb region around the 51 bp DEL event. From top to bottom are Hi-C: Hi-C TADs annotated by purple triangles; chromatin states: 14 chromatin states for 14 tissues from FAANG project; genes (Ensembl): gene annotations from ENSEMBL database; 51-bp DEL-linked SNP: a total of 3584 SNPs were linked to the 51-bp DEL with their color legends listed above; and QTL: 66 QTL regions located in this 2-Mb SV-SNP linked region. The red line indicates the location of the 51-bp DEL, and the black asterisk denotes the eQTL “2:9621709C>T” which was associated with the loin muscle area by GWAS. The eGene *FADS3* is highlighted in green, and its expression was associated with the loin muscle area in the embryo by TWAS

We discovered an intriguing example of the “2:9513511–9513561:DEL” deletion linked with the leading eQTL “2:9621709C>T”, an SNP significantly associated with the loin muscle area trait by GWAS. This SNP was also validated by its corresponding eGene, *FADS3* (fatty acid desaturase 3) in TWAS, which further confirmed a significant association between *FADS3* and the loin muscle area in embryo tissue (Additional file 3: Table S35). This 51-bp deletion “2:9513511–9513561:DEL” directly overlaps strong enhancer regions “2:9513400–9513600” in muscle, “2:9513400–9513800” in the ileum and jejunum, as well as various other types of enhancers in 13 other tissues (Fig. 7 and Additional file 3: Table S37). Additionally, this deletion “2:9513511–9513561:DEL” is linked to 3584 SNPs (*r*^2^ ≥ 0.2, see the “Methods” section for definitions of “linked,” “tagged,” and “highly-tagged”), including 126 eQTLs, 30 sQTLs, and 10 GWAS loci, which have the potential to regulate the expression of 51 genes, splice 20 isoforms, and influence 3 traits (average backfat thickness, lean meat percentage, and loin muscle area) (Additional file 3: Table S38). The 3584 SV-linked SNPs span a near 2 Mb genomic region ranging from 8,525,445 to 10,513,127 (a total of 1,987,683 bp). Moreover, we identified 66 QTL regions located in this SV-SNP linked region, including obesity index, dihomo-gamma-linolenic acid content, arachidonic acid to dihomo-gamma-linolenic acid ratio, etc. (sorted by the distance to “2:9513511–9513561:DEL”) (Additional file 3: Table S39). The *FADS3* gene belongs to the fatty acid desaturase gene family, which has been identified as a strong candidate gene for lipid metabolism in pig muscle and backfat [56, 57]. Furthermore, 119 tagged SVs (*r*^2^ ≥ 0.5) were linked to 595 functional SNPs, 318 e/sGenes and 8 GWAS traits for 425 EUC pigs, and 323 highly-tagged SVs (*r*^2^ ≥ 0.8) were linked to 2003 SNPs involved in the expression of 722 genes, splicing of 244 genes, and 8 GWAS traits for 187 Yorkshire pigs (Additional file 3: Tables S40 and S41)

## Discussion

Selective (natural and human-imposed) and non-selective forces (demographic events and introgression) have driven changes within the pig genome. Their combined effects have created extensive phenotypic diversity and genetic adaptation to local environments across the globe within the modern pig breeds. Genomics has dramatically improved animal health, production, and well-being by shortening the generation interval, identifying genetic markers, and illustrating molecular mechanisms underlying economic traits [58].

Although short-read sequencing presents challenges in the accurate identification of SVs, it is currently the most practical way to assess SV diversity, characterize retention and loss of genomic sequence during domestication, and evaluate the effects of selection on SV at the population scale. The pipeline we developed combines different bioinformatic strategies to improve the accuracy and sensitivity of short read-based SV detection, using strict filtering criteria for SR, RP, and RD approaches to reduce false-positive rates. This pipeline was applied to 1060 pig samples from 101 breeds representing global pig subpopulations. The pipeline generates individual SV genotypes for each animal, which were used to search for population-specific frequency differentiation that represents a signature of local adaptation.

### Improved SV detection to enhance the SV catalog

This study combined four strategies into an integrated SV prediction pipeline and identified 130,556 SV events across 1096 genomes. It was observed that Asian pigs contained more SVs relative to the reference assembly, while European pigs had a lower incidence, likely because the pig reference assembly was derived from the Duroc female of European origin. The differences in SV counts are consistent with population demography, as previously suggested in humans [27]. More deletions were observed compared to duplications and inversions, agreeing with previous studies [59, 60], possibly resulting from bias against the detection of duplications in the RP and RD strategies due to the small insert size or weak signal, respectively. A significant under-representation of SVs in genic regions and regulatory elements was found, implying negative selection against SV in these functional regions. These new insights into pig genome biology are valuable for understanding the effects SVs have on gene function, with the prospect of identifying important novel alleles that can be utilized to improve pigs.

MEI complicates SV detection, but true MEI events were accurately predicted by our pipeline (see validation results). The abundance of SVs with high sequence similarity to known MEIs suggests that some SVs are products of MEI activity. The occurrence of MEIs in the upstream regions of genes, where promoters and regulatory motifs reside, indicates that SVs may be critical agents for gene expression pleiotropy which is often observed in stress-responsive genes [61]. Studies in human genomes have found SV and MEIs associated with aberrant expression of nearby genes [16].

### Population genetics of deletions

This study effectively overcame some of the current problems for the SV study, i.e., complexity for genotyping and inconsistent breakpoint mapping for different individuals. Pigs are an excellent model species to study hybridization because European and Asian wild boars diverged ~1.2 Mya, and pigs were domesticated independently in Europe and Asia. Our genotyping and evolution results using SVs generally agreed with the previous SNP-based studies, providing additional validation of the identified SVs. Our population analyses generally divided the animals into European and Asian pigs. Our SV-based findings confirmed both evolutionary histories of pigs in Europe and Asia, revealing the importance of human-mediated introgression to improve meat quality, development, and fertility commercial traits in modern European pig breeds [7].

### Selection signatures

Population differentiation of SV may contribute to the phenotypic variation between populations [62]. SVs were confirmed to be less likely to occur in the exon regions, consistent with the drastic effects they could have on gene expression and function. The harmful or lethal SVs will have more chances to be selectively eliminated, especially when disrupting coding sequences with out-of-frame mutations. Genes with the exon overlapped with the SV were found to be highly enriched in the immune function, which is supported by many research results that the immune gene was highly diverse and complex among individuals [14, 62–64]. Chr7 and chr9 have drawn the attention of the SV studies because they are enriched for major histocompatibility complex (MHC) genes and olfactory receptor (OR) genes. Some of them were caused by the high variable gene families among animals, such as ZNF and β-defensins.

Other interesting genes harboring SVs include *KIT* and *MYO5A*. Duplications of regulatory elements upstream and downstream of the *KIT* gene locus cause the belted and whole white coat color phenotypes in pigs (Additional file 1: Fig. S5) [24]. Like the *KIT* gene, it is interesting to note that coat color differences may also be correlated with the *MYO5A* gene. MYO5A (Myosin VA) is a class of actin-based motor proteins involved in cytoplasmic vesicle transport, spindle-pole alignment, and mRNA translocation [65]. The protein encoded by this gene is abundant in melanocytes and nerve cells. Multiple alternatively spliced transcript variants encoding different isoforms have been reported, but the full-length nature of some variants has not been determined. Mutations in this gene cause albinism and neurological diseases in humans (Additional file 3: Table S42). In dogs and mice, frameshift and microsatellite expansions increased the expression of truncated transcripts and decreased the expression of full-length transcripts in mutant individuals [66, 67]. Our observation suggests that this may be an MEI insertion in EU pigs instead of a DEL in AS pigs. To our knowledge, this is the first time to report a potential example of a retrotransposon insertion in 3′-UTR of the *MYO5A* gene, changing gene alternative splicing and expression in particular tissues and cells, leading to phenotypic changes for complex economic traits.

Fortunately, the Functional Annotation of Animal Genomes (FAANG) project gave us access to a new landscape of 3D genome reorganization that dynamically regulates gene expression by spatial recruitment of enhancers and promoters [68]. Specifically, we identified a Yorkshire-specific copy number gain within *ABCG2*, which could rearrange chromatin interactions and downregulate the *ABCG2* gene. Notably, this DUP did not seem to affect the expression of its neighbor gene *PKD2* (7011 bp downstream of *ABCG2*) but led to the increased expression of *SPP1* (79,547 bp downstream of *ABCG2*) in adipose, embryo, and uterus in Yorkshire when compared to Duroc. Additionally, this DUP shows tissue-specific regulation features, by spatial reorganization of ATAC peaks for enhancers in different tissues.

### SV functional impacts

We performed an initial exploration into how SVs might impact gene expression, functional elements, and phenotype traits through examining their LD on the population level. During the process, we detected the example of the 51 bp deletion (“2:9513511–9513561:DEL”) linked with the leading eQTL “2:9621709C>T”, which regulated the expression of eGene—*FADS3*. Both the eQTL and eGene were confirmed by GWAS and TWAS, respectively. This example showcased the idea of constructing interaction networks among SVs, e/sQTLs, GWAS loci, and TWAS genes aligns well with the overarching goal of the Agricultural Genome to Phenome Initiative (AG2PI) project (Fig. S1).

### Comparative SV analyses

Comparing SVs in humans, cattle, and pigs will advance our knowledge of genome evolution, genetic diversity, functional genomics, and complex diseases and traits. This effort could offer a comprehensive view of how genomic landscapes adapt to environmental factors across species. It will uncover both conserved regions and specific SVs linked to each lineage, providing insights into the genetic mechanisms driving species evolution and breed development. SVs are pivotal in shaping genetic diversity, via processes like recombination, replication errors, or mobile element insertions. Studying SVs will help reveal their impact on gene regulation, protein function, disease susceptibility, and the varied phenotypes observed.

Both cattle and pigs have undergone extensive selective breeding to produce various breeds tailored for specific purposes such as meat production, disease resistance, and reproduction. The diversity within them provides a rich genetic resource for studying traits relevant to growth rate, meat quality, milk production, disease resistance, and reproductive performance. For instance, cattle genomes harbor unique genes associated with specific adaptations, such as genes for rumen development and digestion of cellulose-rich diets. Additionally, certain gene families related to immune, lactation, metabolism, and even production traits are more diverse or unique in cattle. Conversely, in pigs, genes associated with immune response and olfaction show rapid evolution. Pigs have the largest repertoire of functional olfactory receptor genes, reflecting the importance of smell in this scavenging animal.

### Limitations and future directions

To our knowledge, this is the most comprehensive SV catalog in pigs. We integrated our SV catalogs with results from the most up-to-date FAANG and PigGTEx to fully annotate the impacts of SV on genomes, transcriptomes, and phenotypes. It is important to acknowledge the limitations surrounding SV genotyping, phasing, and imputation using short-read data, which have been reviewed extensively before [1, 2, 4, 5]. These limitations stem from (1) short read sequencing; (2) the lack of a direct alignment-based SV call strategy; and (3) the constraint posed by using a single linear haplotype reference genome like Sscrofa11.1. The catalog of SV could be greatly expanded by the application of long-read sequencing technologies when their costs decrease for population-scale sequencing in the future for livestock. As shown in humans and other species [69–73], the two emerging trends are the so-called T2T complete sequence of a genome and the construction of pangenome in one species, which are both tightly linked to SV events. Additionally, direct SV-based e/sQTL mappings using short reads and long reads will be warranted, as shown recently in humans [64, 74, 75].

## Conclusion

In summary, our results indicate that this SV catalog is a valuable resource for studying diversity and evolutionary history in pigs and how domestication, trait-based breeding, and human selection have functionally shaped Asian and European pigs at the SV level. Our study provided proof of concept for utilizing SVs as potential markers in evolutionary studies and breeding programs.

## Methods

### Data and samples

A total of 1208 WGS datasets with at least 10× coverage were retrieved from the data collected for the FarmGTEx project for pigs (PigGTEx) [34]. We obtained 1060 pigs (*Sus scrofa*) and 36 outgroups (*Babyrousa babyrussa, Phacochoerus africanus*, *Potamochoerus porcus*, *Potamochoerus larvatus*, *Porcula salvania, Sus celebensis*, *Sus verrucosus*, *Sus barbatus*, *Sus cebifrons*, and *Sumatr*) individuals after removing samples with low data quality (61 individuals), clustering errors (14), labeling errors (12), hybrid individuals (12), genotype missing rate of more than 15% (9), and SV count less than 10,000 (4). The metadata of the left samples are summarized in Additional file 3: Table S43. Based on their ancestry composition and geographical locations, specific breeds/groups were divided into seven main populations and 112 (101 for pig) sub-populations, including 54 (4.9%) pigs of 17 sub-populations for Asian wild (ASW), 453 (41.3%) pigs of 48 sub-populations for Asian domestic (ASD), 20 (1.8%) pigs of one sub-population (Duroc and Diannanxiaoer) for crossbreed of Asian domestic pig and European commercial pig (ASxEU), 425 (38.8%) pigs of 5 sub-populations for European commercial (EUC), 88 (8.0%) pigs of 21 sub-populations for European domestic (EUD), 20 (1.8%) pigs of 9 sub-populations for European wild (EUW), and 36 (3.3%) outgroup individuals (OTG) from 10 sub-populations (Table 1).

### Data preprocessing

Paired end (PE) reads of each sample were trimmed by Trimmomatic (v 0.39). BWA (v0.7.5a) *mem* was used to align clean reads against the pig reference genome Sscrofa11.1 downloaded from ENSEMBL ftp://ftp.ensembl.org/pub/release-101/fasta/sus_scrofa/dna/Sus_scrofa.Sscrofa11.1.dna.toplevel.fa.gz. Aligned reads were sorted by genome coordinate using Samtools (v 0.1.18). To reduce potential polymerase chain reaction (PCR) or sequencing optical artifacts, the duplicated reads were marked and removed by *MarkDuplicates* function in GATK (v4.1.4.1). To obtain functionally equivalent results [76], we down-sampled 417 deeply sequenced samples (> 20×) into 15× coverage using Picard (v2.25.2) *DownsampleSam* function with parameters RANDOM_SEED = 1 and STRATEGY = ConstantMemory.

### SV calling and genotyping

We utilized an integrated pipeline of Lumpy (v 0.2.13), SVTyper (v0.7.1), and CNVnator (v0.4.1) for per-sample SV calling [26]. Software Lumpy integrating both read-pair (RP) and split-read (SR) strategies, is a probabilistic framework that runs individually for each sample and gives four main types of SV, including DEL (deletion), DUP (duplication), INV (inversion), and BND (break end). To decrease run time and false positives, we utilized smoove (v0.2.6, https://github.com/brentp/smoove) to process alignment information. After individual calling by smoove *call*, we generated a merged set of variants across all samples using SVtools (v0.3.2) *lsort* and *lmerge*. Then we genotyped all variants for each sample using SVtools *genotype*, and annotated with copy number (CN) information from a read depth (RD) strategy CNVnator by SVtools *copynumber*. The SVtools *vcfpaste*, *prune*, and *classify* (large sample mode) were used to paste, filter, and refine the identified SVs with default parameters, respectively. The SVtools *classify* was used to sort DEL into MEI when more than 90% of DEL length was covered by repetitive elements, whose information was from the UCSC genome browser’s pig RepeatMasker database (https://hgdownload.soe.ucsc.edu/goldenPath/susScr11/database/rmsk.txt.gz). Allele balances were calculated as the number of ALT allele-supporting reads divided by the sum of the number of ALT and the number of REF allele-supporting reads.

### Quality control for SVs

We divided the pig reference susScr11 assembly into 100 bp windows using bedtools (v2.30.0) *makewindows*, and genotyped SVs using CNVnator *genotype* across all samples. A total of 5973 regions, within which the CN values in at least 90% (996) samples were higher than 10 (5 times the expected diploid copy number), were considered as “bad bin,” e.g., the pig genomic regions which were similar to human leukocyte antigen (HLA) sequences (Additional file 3: Table S44). SVs overlapping those high CN regions were removed in this analysis. We then filtered away those SVs which overlapped with the gap regions in the reference genome by at least 1 bp (ftp://128.114.119.163/goldenPath/susScr11/database/gap.txt.gz). The lengths of intra-chromosomal generic BNDs were calculated using AWK. We removed events with lengths smaller than 50 bp.

We then filtered each type of SV using the optimal parameters, as recommended by the previous publication [77]. Small DELs ≤ 1000 bp were eliminated unless they had split read support in at least one sample; INVs were kept only if mean sample quality (MSQ) > 150 and provided at least 10% of read support. BNDs were retained if MSQ > 250. MEIs and DELs were kept if MSQ > 100. Meanwhile, MEI and DEL genotypes were set to missing on a per-sample basis if the site was poorly captured by split reads (filter_del.py) or the size of the DEL or MEI was smaller than the minimum size reported by SVTyper at 95% confidence (del_pe_resolution.py).

### Verification of short read-based SVs using long read-based assembly comparison

Using the mummer (v3.23) program, we detected SVs by comparing two high-quality pig assemblies (contig N50 > 48 Mb) based on PacBio long-reads: Meishan MSCAAS v1 [25] vs. Duroc Sscrofa11.1 [36]. We used the *findOverlaps* function, implemented in the R package GenomicRanges (v1.40.0), to overlap (at least 1bp) Meishan WGS SVs and SVs derived from long-read-based assembly comparison. We measured the consistency rate for each overlapped WGS SV with long read-based assembly comparison results.

### SV annotation

We annotated repeat- and gene-overlapping SVs using the *findOverlaps* function of the R package GenomicRanges, (v1.40.0), with at least 1 bp threshold. The gene annotation of the *Sus scrofa* genome was downloaded from Ensembl (http://ftp.ensembl.org/pub/release-101/gtf/sus_scrofa/Sus_scrofa.Sscrofa11.1.101.gtf.gz). The multiple gene versions could cause multiple genes to overlap in one region. So, we removed those old genes (*n* = 934) that had new version annotation or unplaced sequence genes (*n* = 1448) from a total of 31,908 genes, with 29,526 genes and 19,360 protein-coding genes remaining in the pig genome (Additional file 3: Table S45). As described previously [26, 27], we carried out fold tests to estimate the enrichment of genome characteristic regions (GCR) in SV with the whole genome as background using the Chi-squared Test. The fold of GCR in SV to the genome was calculated by the proportion of GCR in SV divided by the proportion of GCR in the whole genome. The significance *P* values were adjusted by the Bonferroni methods. The fold tests were also performed on all genomic annotations of interest. Gene ontology (GO) enrichment and Kyoto Encyclopedia of Genes and Genomes (KEGG) pathway analyses were performed for gene lists of interest, respectively, using *enrichGO* and *enrichKEGG* functions from the R (v4.0.2) package clusterProfiler (v3.16.1). *P* values were adjusted by the Bonferroni method, and the threshold was set to 0.01.

### Classifying gene-overlapping SVs

Following the previous publication [26], we classified those gene-overlapping SVs into 15 categories based on their location within a gene, including whole gene DEL (WlGnDel), DUP (WlGnDup), and INV (WlGnInv), predicted loss-of-function (pLoF) when DEL or MEI occurred in the coding sequence (CDS), copy gain (CpGn) when DUP occurred in CDS, coding INV (codInv), coding BND (codBnd), 5′ Regulation (5′Rglt), and 3′ Regulation (3′Rglt). CDS-mapped SVs were defined as weak (Wk) impact if overlapped CDS accounted for less than 20% of its own length. In contrast, they defined a strong (St) impact when overlapped CDS accounts for greater than 20%. Similarly, UTR-mapped SVs were defined as weak (Wk) impact if mapped untranslated region (UTR) lengths were less than 20%. The significant difference test for singleton proportion is carried out by the Student’s *t*-test for each category as compared to intergenic regions. The Bonferroni-corrected *P* values ≤ 0.01 were considered significant.

### SV overlapping chromatin states

We also analyzed 15 chromatin states predicted using ChIP-seq for four types of histone modification marks (i.e., H3K4me3, H3K4me1, H3K27ac, and H3K27me3) and chromatin accessibility (ATAC-seq), as previously reported [68, 78], including active promoters/transcripts (TssA), flanking active transcription start sites (TSS) without ATAC (TssAHet), transcribed at gene (TxFlnk), weak transcribed at gene (TxFlnkWk), transcribed region without ATAC (TxFlnkHet), strong enhancer (EnhA), medium enhancer with ATAC (EnhAMe), weak active enhancer (EnhAWk), active enhancer no ATAC (hetero) (EnhAHet), poised enhancer (EnhPois), ATAC island (ATAC_Is), bivalent/poised TSS (TssBiv), repressed polycomb (Repr), weak repressed polycomb (ReprWk), and quiescent (Qui) in 14 pig tissues. Pairwise comparisons were carried out for each SV type and each chromatin state as compared to Qui. At least one bp overlapping of SV and chromatin state was considered here.

We further annotated gene features using those active promoters and strong enhancers. Based on their relative locations, we divided promoters or enhancers into the following regions: gene flanking (located in 5 kb flanking regions of a gene on two sides), exon, 5′-UTR, 3′-UTR, intron, and intergenic region. We calculated the count ratio of SV-related promoters and enhancers to all promoters and enhancers for all gene locations. We then tested the significance of the ratio for each gene location as compared to the total, based on the Student’s *t*-test with a threshold of Bonferroni-corrected *P* < 0.01.

### Integrating SV with QTL

We retrieved a total of 32,874 QTLs for 704 traits from the animal genome database (www.animalgenome.org, release 47) [40]. To avoid potential bias, we removed QTLs that were larger than 1 Mb and kept 22,176 QTLs for 565 traits. We calculated the fold enrichment of QTLs and DEGs in nonredundant SVs (at least 1 bp overlap) as compared to the whole genome and tested significance by the Chi-squared Test. QTLs were considered as significantly enriched in SVs if their fold enrichment in SVs as compared to those in the whole genome > 2 with the Bonferroni corrected *P* value < 0.01. We compared SV enrichments in each of the QTL traits among 6 main populations, based on the analysis of variance (ANOVA) type III test, with the Bonferroni-adjusted threshold *P* < 0.01. We kept QTL traits with the coefficient of variation of group mean log_2_FC larger than one among 6 main populations.

### Population structure based on SV genotypes

We performed population genetic analysis for 1060 pigs and 66 outgroup individuals of 112 sub-populations from 7 main populations, starting from a total of 193,550 (including Outgroup) autosomal SV genotypes. After removing 86,046 SVs with minor allele frequency (MAF) < 0.01 by PLINK (v1.90b6.21), we retained 107,504 SVs with the genotype rate of individuals > 0.95 for the subsequent analyses. We first ran principal component analysis (PCA) for all these 1096 individuals and then just 1060 pigs using PLINK. We used fastStructure (v1.0) for the population structure analysis based on PLINK format, with parameters --seed=1 (specify seed for random number generator) --full (output all variation parameters) --tol=10e-8 (convergence criterion), and set K from 2 to 7, and 10. The maximum likelihood tree was inferred by TreeMix (v1.12) and plotted by R package ggtree (v3.1.4.991), based on the allele frequency of each SV calculated by PLINK (v1.07). Pairwise fixation index (*F*_*ST*_) was also calculated by PLINK between groups for common SV genotypes (without Outgroup, MAF ≥ 0.01). The top 1% of them were identified as the SVs with high *F*_*ST*_.

### Definitions of lineage-specific SVs

In the AS and EU comparison, we removed ASxEU pigs as they were crossbred between Diannanxiaoer and Duroc. We defined group-specific SVs as those events that were only detected in one group. For instance, if an SV was detected in at least one pig of the AS group but not in any EU pig, we labeled it AS-specific SV. If an SV was presented in both AS and EU, we defined it as a shared SV.

### RNA-seq data analysis

We obtained 7095 RNA-seq samples from the PigGTEx project [34], and among them, 2101 (from 26 tissues in 19 sub-populations) had the matched sub-population information as the 1060 pigs with WGS data. To ensure the power of subsequent analyses, each tissue in each sub-population had at least three RNA-seq samples, and each sub-population had at least 15 WGS individuals. Luchuan pigs with low RNA-seq read depth and the crossbreed of Duroc and Diannanxiaoer were filtered away, resulting in 1635 RNA-seq samples from 19 tissues in 8 sub-populations.

Gene expression was defined by normalized read count for full gene length, which was calculated by the *cpm* function from R package edgeR (v3.30.3). Genes with low expression levels were filtered by the *filterByExpr* function in edgeR, with default parameters min.count (Minimum count required for at least some samples) = 10 and min.total.count (Minimum total count required) = 15.

### DEGs between AS and EU pigs

To detect differentially expressed genes (DEGs) between AS and EU, we kept those tissues present at least in two sub-populations for both AS and EU groups. We focused on 1171 RNA-seq samples from 12 tissues (i.e., embryo, skin, adipose, liver, muscle, ovary, small intestine, uterus, brain, heart, spleen, and testis) in 8 sub-populations (i.e., Meishan, Erhualian, Wuzhishan, Bamaxiang, Yorkshire, Duroc, Composite, and Pietrain).

The DEGs between AS and EU pigs were detected using *exactTest* in the R package edgeR. The raw *P* values were adjusted by the Bonferroni method, and the adjusted *P* < 0.01 was considered significant. The common (AF ≥ 0.01) SV-related DEGs were kept for downstream analyses.

### DEGs between sub-population pairs

We employed edgeR to detect DEGs after removing low-expression genes in each tissue for sub-population pairs. We performed pairwise DEG analyses in each tissue between two paired populations, with one population having its specific SV (AC > 1) or high *F*_*ST*_ SV against other populations. We removed Composite pigs because they were crossbred among the Duroc, Landrace, and Yorkshire breeds. In total, we considered 1141 RNA-seq samples from 14 tissues (with placenta and blood added) in 7 sub-populations in this exercise.

### SV-related gene examples

We illustrated the junction read distribution of RNA-seq data for randomly selected samples using the integrative genomics viewer (IGV, v2.11.3). Chromatin states derived using ChIP-seq (H3K4me3, H3K4me1, H3K27ac, H3K27me3, input control) and ATAC-seq of 14 tissues from two Yorkshire male biological replicates and high-throughput chromosome conformation capture sequencing (Hi-C) data of pig liver were illustrated through the UCSC Genome Browser, as previously reported [68]. Official gene features were retrieved from the Ensembl. We reassembled the *MYO5A* gene transcripts using StringTie (v2.1.7) for the embryo, heart, skin, and uterus samples (*N* = 296) (Additional file 3: Table S21).

The MEME online tool (https://meme-suite.org/meme/) was used to search for enriched gene ontology terms of motifs in the sequence of the 303-bp DEL in the *MYO5A* gene.

The consensus sequence of aligned reads for each sample was extracted by samtools v1.15.1, and then aligned with each other by ClustalX v2.1 with default parameters.

### Integrating SVs with e/sQTLs, GWAS loci, and TWAS genes

We first calculated LD (r^2^) for all SVs and SNPs for the combined genotype matrix within a 1 Mb genomic distance on three population levels, by PLINK (v1.90b6.21) with parameters “--r2 --ld-window-kb 1000 --ld-window 99999 --ld-window-r2 0”. The three population groups include the entire set of 1060 pigs, a combination of EU and AS pigs representing ancestral diversity; 425 EUC pigs with the same ancestor; and a specific breed, Yorkshire, comprising 187 pigs, which has the largest sample size among the breeds in our dataset. Because it is a huge data that reported LD *r*^2^ more than 0 for all SVs and all SNPs, we reduced the number of SNPs by retaining one SNP per every 1000 bp using the command vcftools (v0.1.16) “--thin 1000”.

To integrate SVs and functional SNPs, we obtained eQTL and sQTL data for 34 tissues and GWAS results for 14 traits from the PigGTEx project [34]. We retained independent e/sQTLs and GWAS loci (*P* ≤ 5 × 10^−8^) if their LD *r*^2^ was higher than 0.2 with SVs within a 1-Mb genomic distance (SNPs was not thinned as the LD list with an *r*^2^ of 0.2 is not extensive). We defined SV-SNP pairs with *r*^2^ ≥ 0.2 as “linked”, *r*^2^ ≥ 0.5 as “tagged”, and *r*^2^ ≥ 0.8 as “highly-tagged” within our dataset.

To annotate SVs, we also retrieved TWAS data of 34 tissues for 14 traits from the PigGTEx project [34]. We kept TWAS genes (false discovery rate, FDR ≤ 0.05) if they were also detected as eGene or sGene. Furthermore, TWAS traits matching GWAS traits were preserved for subsequent analysis. TWAS examines the association between gene expressions and phenotype traits, establishing pairs of genes and phenotype traits. On the other hand, GWAS investigates the association between SNP genotypes and phenotypes, forming pairs of SNPs and phenotype traits. The e/sQTL mapping explores SNP genotypes and molecular phenotypes like gene expression and isoform alternative splicing, generating pairs of e/sQTL and their corresponding e/sGenes. We attempted to construct interaction networks among SVs (*r*^2^ ≥ 0.2), e/sQTLs-e/sGenes, GWAS loci, and TWAS genes (Fig. S1). We defined an overlap between an SV and one e/sGene or TWAS gene, as either the SV directly overlapped with the gene body or overlapped with enhancer/promoter regions ranging from 5 kb upstream to 5 kb downstream of the gene body.

### Supplementary Information

**Additional file 1:** **Fig. S1**. Workflow. **Fig. S2**. Comparison of SV content across 7 main populations. **Fig. S3**. SV-related QTL and GWAS traits. **Fig. S4**. Gene enrichment analyses for group-specific SVs. **Fig. S5**. Illustration of the genomic region for whole gene DUP of *KIT*. **Fig. S6**. Reassembly of the *MYO5A* gene transcripts. **Fig. S7**. Gene expressions of *ABCG2*, *PKD2*, *SPP1*, and *PPM1K* for 9 tissues. **Fig. S8**. LD decay for SVs and SNPs in 1060 pigs, 425 EUC pigs, and 187 Yorkshire pigs. Fig. S9. LD r^2^ at different genome distances for SVs and SNPs in Yorkshire, EUC, and 1060 pigs.

**Additional file 2:** **Table S1**. Non-redundant SV statistics for sub-populations. **Table S2**. SV statistics for individuals. **Table S3**. SV list. **Table S4**. Overlaps between WGS SVs derived from 30 Meishan pigs and SVs derived from long-read-based assembly comparison of MSCAAS v1 and Sscrofa11.1. **Table S5**. Summary statistics of overlapping rate between Meishan WGS SV and SVs derived from long-read-based assembly comparison of MSCAAS v1 and Sscrofa11.1. **Table S6**. Statistics of SVs overlapped by repeats. **Table S7**. GO enrichment results of total SV overlapped genes. **Table S8**. GO enrichment results of singleton SV overlapped genes. **Table S9**. Summary Statistics of SVs overlapped by protein coding genes. **Table S10**. Categories of gene-overlapping SVs. **Table S11**. Proportions of SVs overlapped by various chromatin states for tissues. **Table S12**. Proportions of singleton SVs overlapped by various chromatin states for tissues.

**Additional file 3:** **Table S13**. Locations of SV-related enhancers and promoters. **Table S14**. Proportions of SV-related regulators in various locations of tissues. **Table S15**. Statistics of SV-related QTL. **Table S16**. QTL mapping SV for individual genome. **Table S17**. Locations of group-specific or high *F*_*ST*_ SVs. **Table S18**. Summary of group-specific or group-differential SVs. **Table S19**. GO enrichment results of genes in group-specific or group-differential SVs. **Table S20**. KEGG pathway analysis of genes mapping/flanking group-specific or group-differential SVs. **Table S21**. RNA-seq sample list in AS and EU ancestral group comparison. **Table S22**. Statistics of RNA-seq samples for AS and EU comparison. **Table S23**. The SV and gene list for AS and EU comparison. **Table S24**. SV-related DEGs for AS and EU ancestral group comparison. **Table S25**. Statistics of multiple-tissue SV-related DEGs between AS and EU groups. **Table S26**. Statistics of SV-related multiple-tissue DEGs with discordant expression levels between tissues. **Table S27**. TWAS annotation of SV-related multiple-tissue DEGs between AS and EU pigs. **Table S28**. Allele frequency of MYO5A DEL in each population. **Table S29**. RNA-seq sample list in SV-related analyses for sub-populations. **Table S30**. SV-related DEGs for sub-population comparisons. **Table S31**. Length statistics for chromatin states in the chr8:130924619-130980283 DUP region. **Table S32**. Median r^2^ values for 1060 pigs, 425 EUC pigs, and 187 Yorkshire pigs at different genomic distances. **Table S33**. Proportions of SV with linked, tagged, and highly-tagged r^2^ with SNPs. **Table S34**. Proportions of SV with linked, tagged, and highly-tagged r^2^ with functional SNPs. **Table S35**. List of SV-linked functional SNPs with multiple signals for 1060 pigs. **Table S36**. List of SV directly overlapped e/sGenes for their linked SNPs in 1060 pigs. **Table S37**. List of the 51 bp DEL overlapped enhancers in 14 tissues. **Table S38**. List of the 51 bp DEL-linked SNPs. **Table S39**. List of QTLs mapped within the 2 Mb region. **Table S40**. List of SV-linked functional SNPs with multiple signals for 425 EUC pigs. **Table S41**. List of SV-linked functional SNPs with multiple signals for 187 Yorkshire pigs. **Table S42**. MYO5A functions based on GWAS results of EMBL-EBI. **Table S43**. List of data information for all analyzed samples. **Table S44**. Pig genome bad bins in our dataset. **Table S45**. All analyzed genes.

**Additional file 4.**

## Data Availability

The data that support the results of this research are available within the article and its Supplementary Information files. All raw data analyzed in this study are publicly available for download without restrictions from the SRA (https://www.ncbi.nlm.nih.gov/sra/) and BIGD (https://bigd.big.ac.cn/bioproject/) databases. Details of RNA-seq, WGS, WGBS, single-cell RNA-seq, and Hi-C datasets can be found in Supplementary Tables 1, 2, 5, 8, and 9 of the PigGTEx paper, respectively [34]. All WGS data information is also shown in Table S43. Additionally, newly generated WGS data by this study are available under CNCB GSA (https://ngdc.cncb.ac.cn/) under accessions PRJCA016012, PRJCA016120, PRJCA016130, PRJCA016216, and PRJCA017284.

## Abbreviations

3′Rglt: 3′ Regulation
5′Rglt: 5′ Regulation
AFgt: Allele frequency greater than 0.95
AFle: Allele frequency less than 0.05
ANOVA: Analysis of variance
ASD: Asian domestic
ASW: Asian wild
ASxEU: Crossbreed of Asian domestic pig and European commercial
ATAC_Is: ATAC island
ATAC-seq: Transposase-accessible chromatin with high-throughput sequencing
BND: Breakend
CDS: Coding sequence
CN: Copy number
codBnd: Coding BND
codInv: Coding INV
CpGn: Copy gain
DEG: Different expression gene
DEL: Deletion
DUP: Duplication
EnhA: Strong enhancer
EnhAHet: Active enhancer no ATAC (hetero)
EnhAMe: Medium enhancer with ATAC
EnhAWk: Weak active enhancer
EnhPois: Poised enhancer
eQTL: Expression quantitative trait loci
EUC: European commercial
EUD: European domestic
EUW: European wild
FarmGTEx: Farm animal genotype-tissue expression project
FST: Pairwise fixation index
GCR: Genome characteristic regions
GO: Gene ontology
HLA: Human leukocyte antigen
IGV: Integrative genomics viewer
INV: Inversion
KEGG: Kyoto Encyclopedia of Genes and Genomes
LINE: Long interspersed nuclear element
Log2FC: Log2 fold change
MAD: Median absolute deviations
MAF: Minor allele frequency
MEI: Mobile element insertions
MSQ: Mean sample quality
OTG: Outgroup
PCA: Principal component analysis
PCR: Potential polymerase chain reaction
PE: Paired end
PigGTEx: FarmGTEX for pig
pLoF: Predicted loss-of-function
QTL: Quantitative trait loci
Qui: Quiescent
RD: Reads depth
Repr: Repressed polycomb
ReprWk: Weak repressed polycomb
RP: Read-pair
SINE: Short interspersed nuclear element
sQTL: Splicing quantitative trait loci
SR: Split-read
St: Strong
TSS: Transcription start sites
TssA: Active promoters/transcripts
TssAHet: Flanking active TSS without ATAC
TssBiv: Bivalent/poised TSS
TxFlnk: Transcribed at gene
TxFlnkHet: Transcribed region without ATAC
TxFlnkWk: Weak transcribed at gene
UTR: Untranslated regions
WGS: Whole genome sequencing
Wk: Weak
WlGnDel: Whole gene DEL
WlGnDup: Whole gene DUP
WlGnInv: Whole gene INV
DEG: Differentially expressed gene
TWAS: Transcriptome-wide association studies
GWAS: Genome-wide association studies
eQTL: Expression quantitative trait loci
sQTL: Splicing quantitative trait loci
PigGTEx: Pig Genotype-Tissue Expression (PigGTEx) project
LD: Linkage disequilibrium
