# Supplementary material for: Mapping and functional characterization of structural variation in 1060 pig genomes
Source: Genome Biol. 2024 May 7;25:116. doi: 10.1186/s13059-024-03253-3 (PMC11075355; doi:10.1186/s13059-024-03253-3)
Supplement: Supplementary file 1 — Additional file 1: Fig. S1. Workflow. Fig. S2. Comparison of SV content across 7 main populations. Fig. S3. SV-related QTL and GWAS traits. Fig. S4. Gene enrichment analyses for group-specific SVs. Fig. S5. Illustration of the genomic region for whole gene DUP of KIT. Fig. S6. Reassembly of the MYO5A gene transcripts. Fig. S7. Gene expressions of ABCG2, PKD2, SPP1, and PPM1K for 9 tissues. Fig. S8. LD decay for SVs and SNPs in 1060 pigs, 425 EUC pigs, and 187 Yorkshire pigs. Fig. S9. LD r2 at different genome distances for SVs and SNPs in Yorkshire, EUC, and 1060 pigs. [file 13059_2024_3253_MOESM1_ESM.docx]

## Supplementary Figures

###
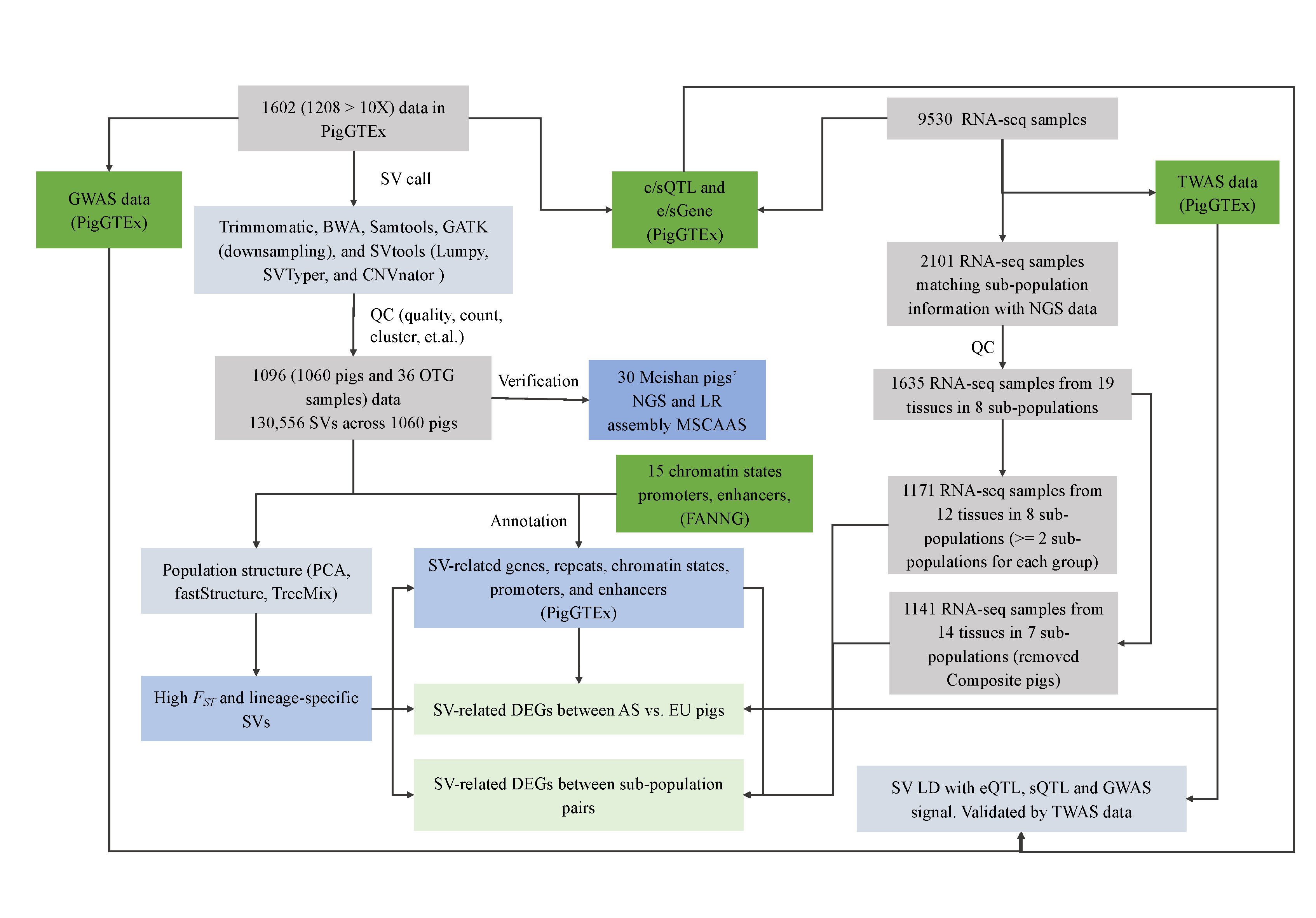
Fig. S1. Workflow.

###
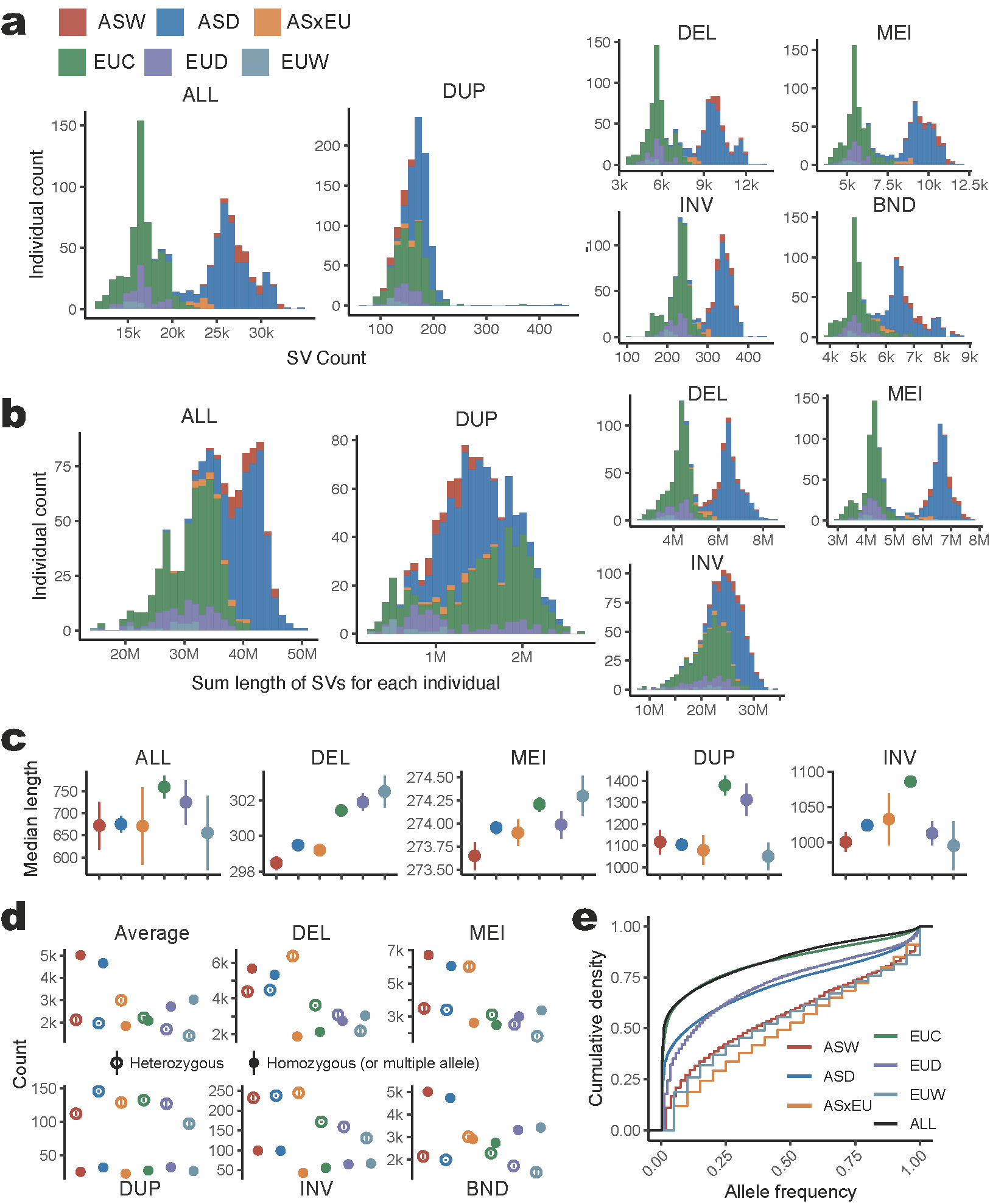
Fig. S2. Comparison of SV content across 7 main populations.

**a.** Individual count histogram of for SV count. The y axis denotes the relative frequencies in each main population. The x-axis denotes SV counts for all SVs or each SV type. As the reference genome is from a Duroc, the expectation of distribution is that EU groups remain on the left side of x-axis, while AS groups on the right side and ASxEU pigs are in the middle. DEL, MEI, INV, and BND matched the expectation, but DUP did not.

**b.** Relative frequency histogram of total SV length. The y axis denotes the relative frequencies in each main population. The x-axis denotes SV lengths for all SVs.

**c.** Median length for each SV type across 7 main populations. The SV median lengths of EU pigs were generally larger than those of AS pigs. Especially, DUPs in EUC and EUD were more than 200 bp longer than DUPs in AS pigs.

**d.** Count of heterozygous and homozygous SVs. Multiple allele DUPs were combined into the homozygous.

**e.** Accumulative density of SV allele frequency for each main population.

###
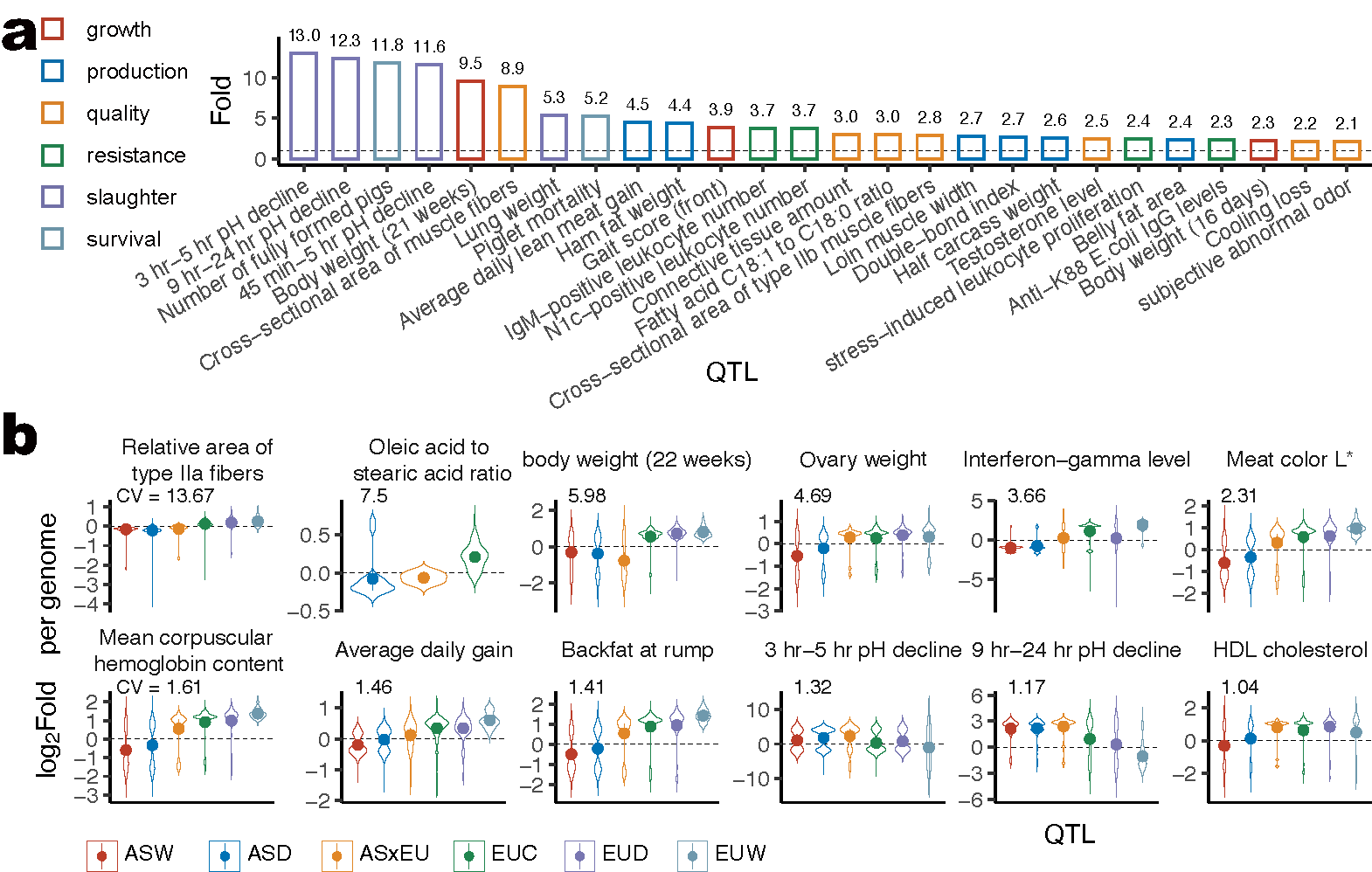
Fig. S3. SV-related QTL and GWAS traits.

**a.** Enrichments of QTL in SVs as compared to the whole genome. Enrichment folds were calculated as the length proportion of QTL in SV divided by the length proportion of QTL in the genome. The 26 QTLs are shown with the Bonferroni corrected Chi-squared *P* ≤ 0.01 and enrichment fold higher than 2. See **Table S17** for details.

**b.** Enrichments of QTL in SVs as compared to the whole genome for 6 main populations. Enrichment folds were calculated as the proportion of QTL length in SV divided by the proportion of total QTL length in the genome for each individual population. Based on the ANOVA type III test, a total of 12 QTLs were kept by the Bonferroni corrected *P* ≤ 0.01 and coefficient of variation ≥ 1. Bars represent sample mean and lower and upper Gaussian 95% confidence limits based on the t-distribution. The value texts on the top left denote the coefficients of variation (CV) between 6 main populations.


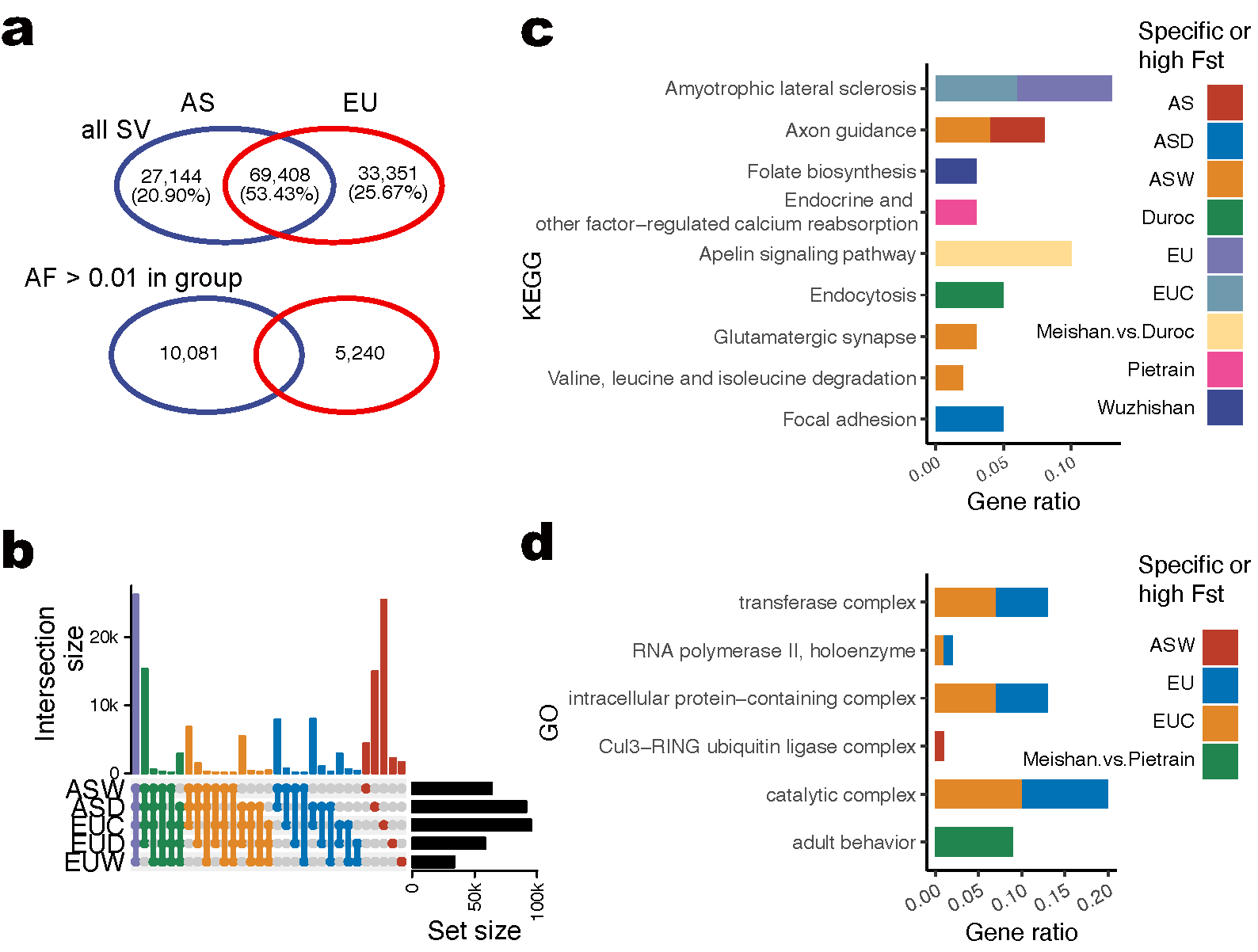


### Fig. S4. Gene enrichment analyses for group-specific SVs.

**a.** Venn plots of SV counts and proportions of AS-, EU-specific, or shared SV.

**b.** Upset plot SV counts for ASW, ASD, EUC, EUD, and EUW pigs.

**c.** KEGG pathway results. Calculated by *enrichKEGG* function from R package clusterProfiler v3.16.1. *P* values were adjusted by the Bonferroni method, and the threshold was set as 0.01.

**d.** GO enrichment results. Calculated by *enrichGO* function from R package clusterProfiler.


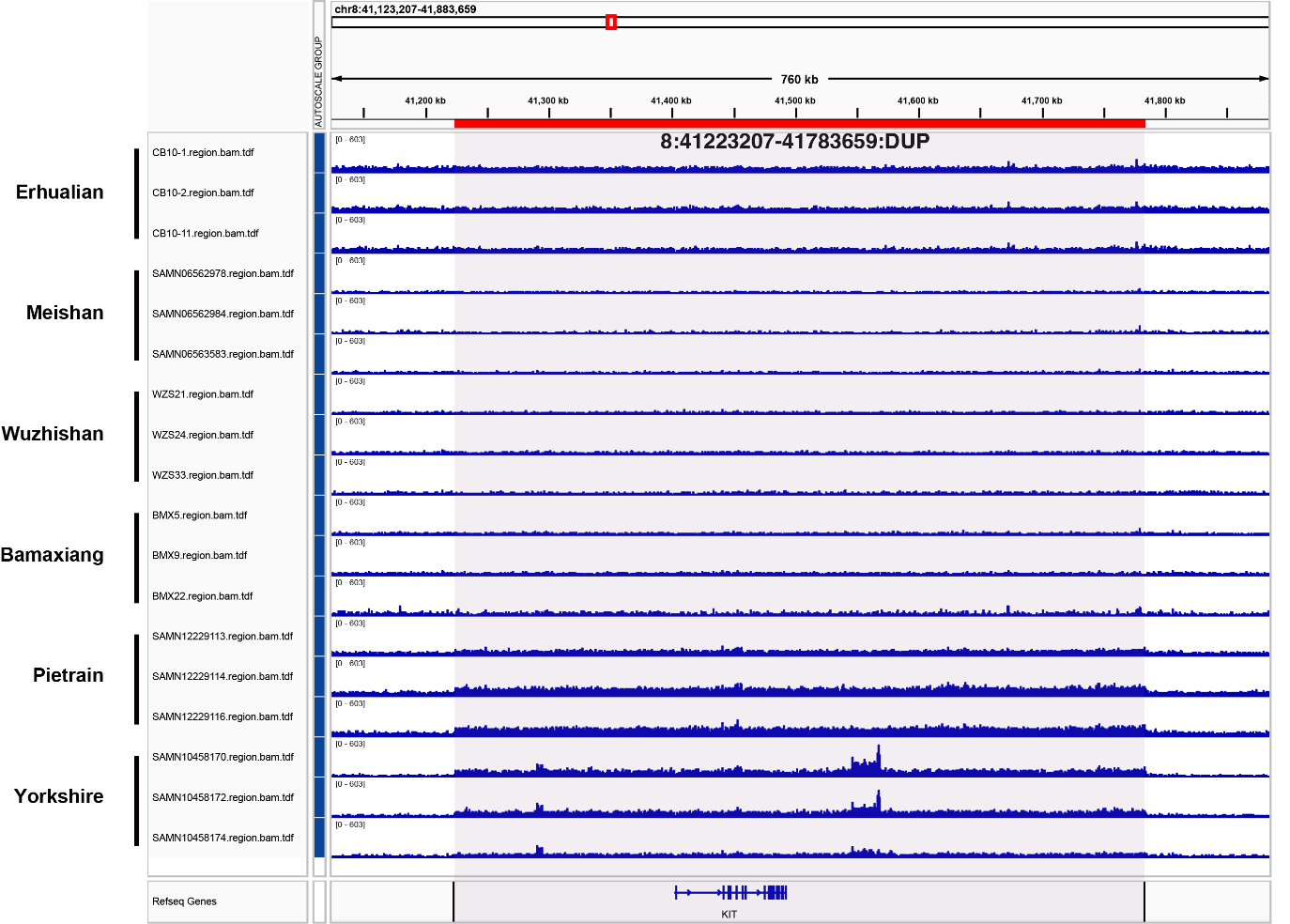


**Fig. S5.** Illustration of the genomic region for whole gene DUP of *KIT*.

In the Integrative Genomics Viewer (IGV), aligned reads were derived from three randomly selected individuals each belonging to six different breeds: Erhualina, Meishan, Wuzhishan, Bamaxiang, Pietrain, and Yorkshire. Notably, the red box and background highlight the genomic region corresponding to a whole-gene duplication (DUP) at coordinates 8:41223207-41783659.
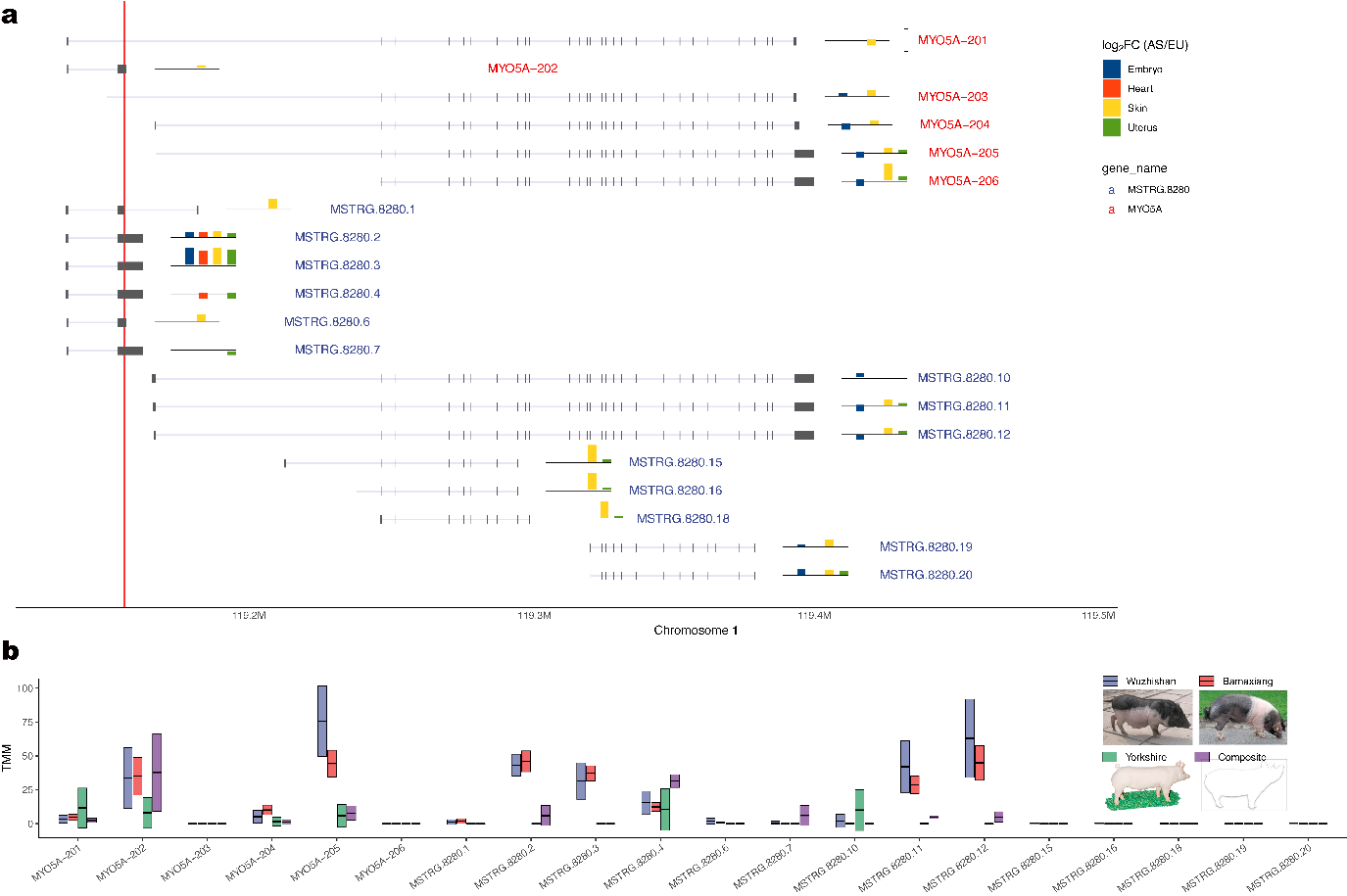


### Fig. S6. Reassembly of the *MYO5A* gene transcripts.

**a.** Reassembly of the *MYO5A* gene transcripts. The bar and line on the right of each transcript indicate the log_2_FC of normalized expression of AS and EU (AS/EU). Bars on the top of line denote higher transcript expressions in AS compared to EU.

**b.** Expression levels of MYO5A transcripts from official annotations and reassembled transcripts MSTRG.8280s.


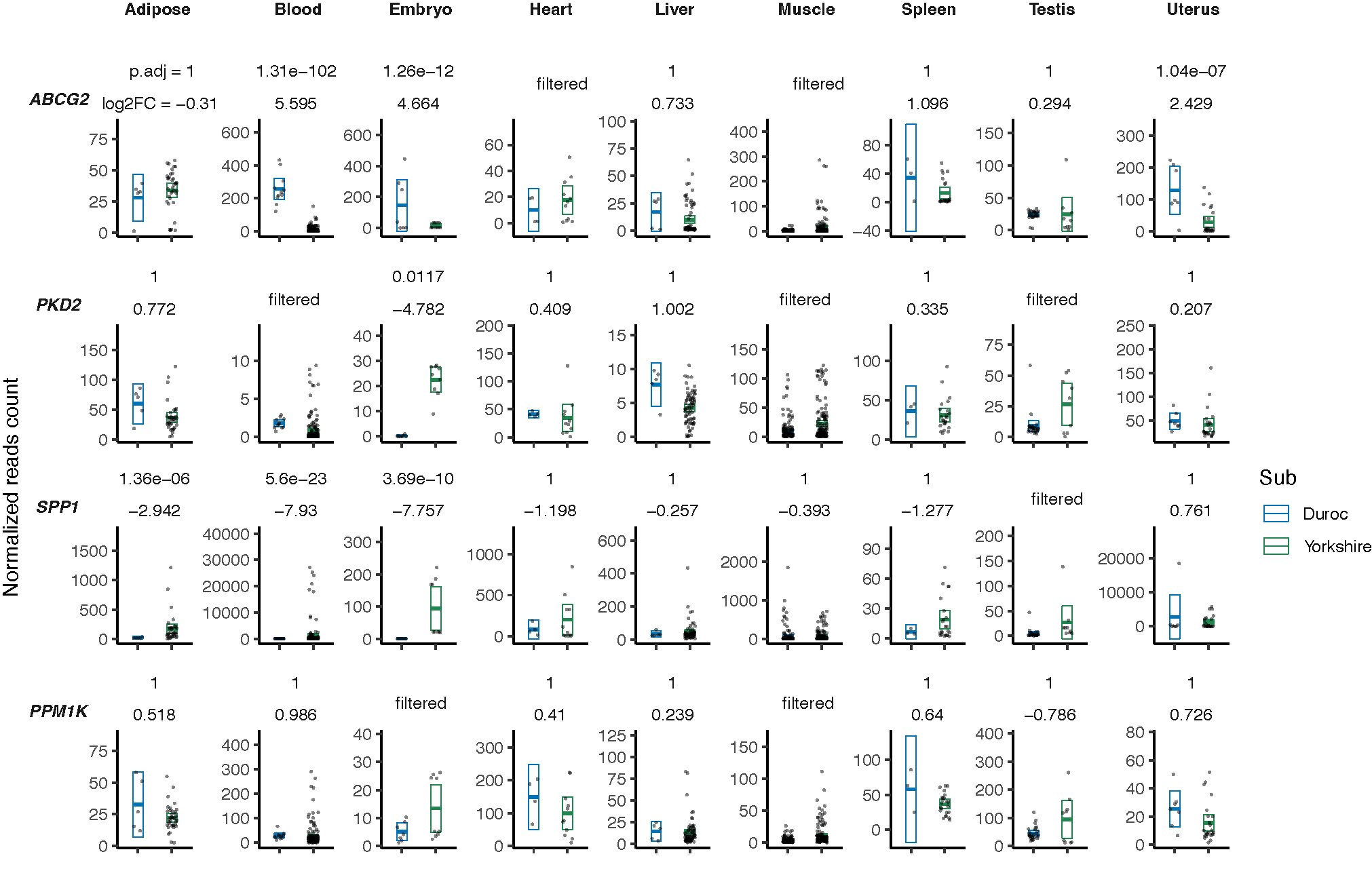


### Fig. S7. Gene expressions of *ABCG2*, *PKD2*, *SPP1*, and *PPM1K* for 9 tissues.

The x-axis denotes sub-populations of Duroc and Yorkshire, and the y-axis denotes the normalized read counts of this gene.


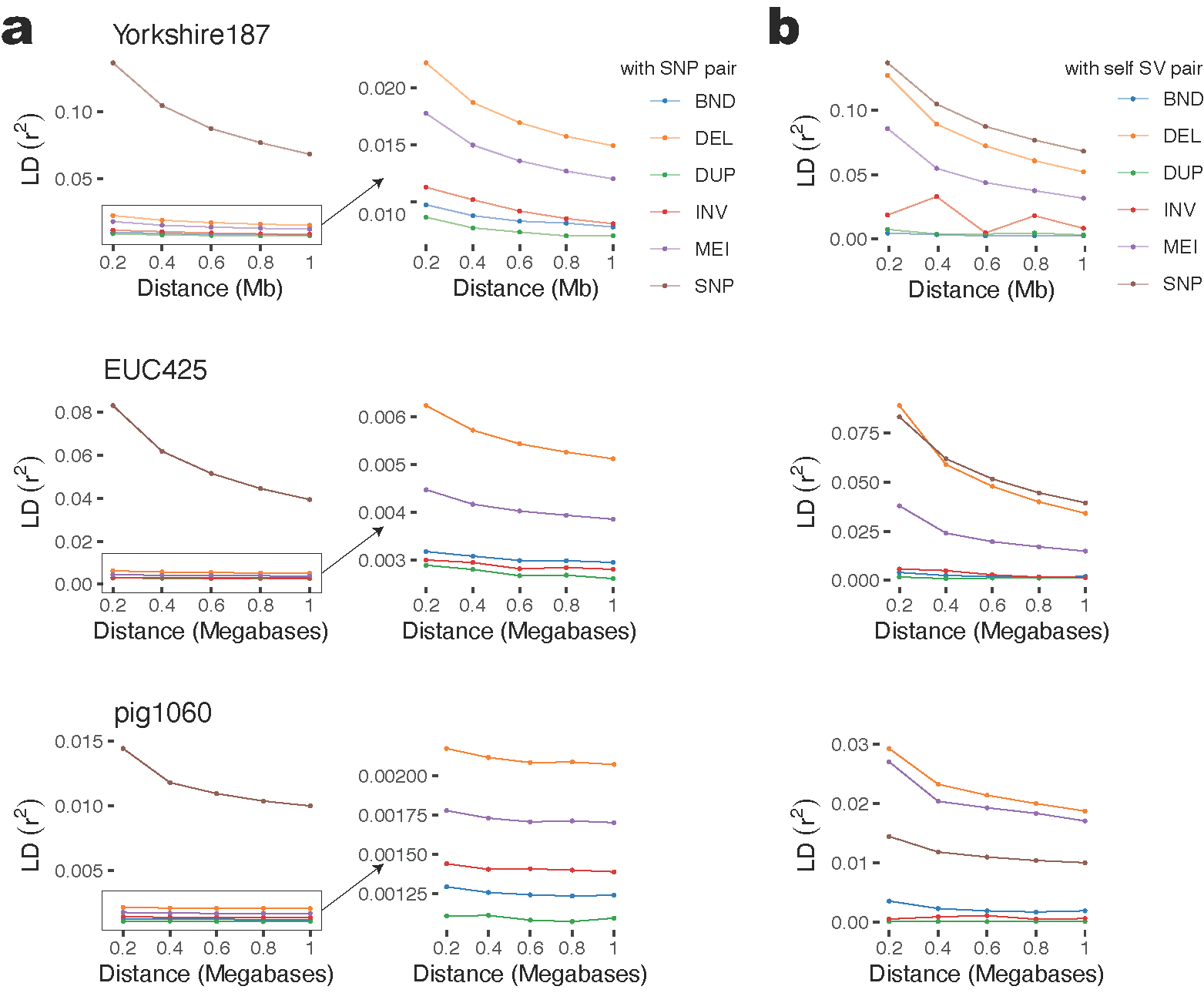


### Fig. S8. LD decay for SVs and SNPs in 1060 pigs, 425 EUC pigs, and 187 Yorkshire pigs.

Generated using PLINK --r2 with a threshold of 0, indicating the reporting of all pairs of SNPs and SVs within a 1 Mb distance (--ld-window-kb 1000). (a) The LD decay of each type of SV with SNP pairs. (b) The LD decay of SV and SV pairs within each SV type.


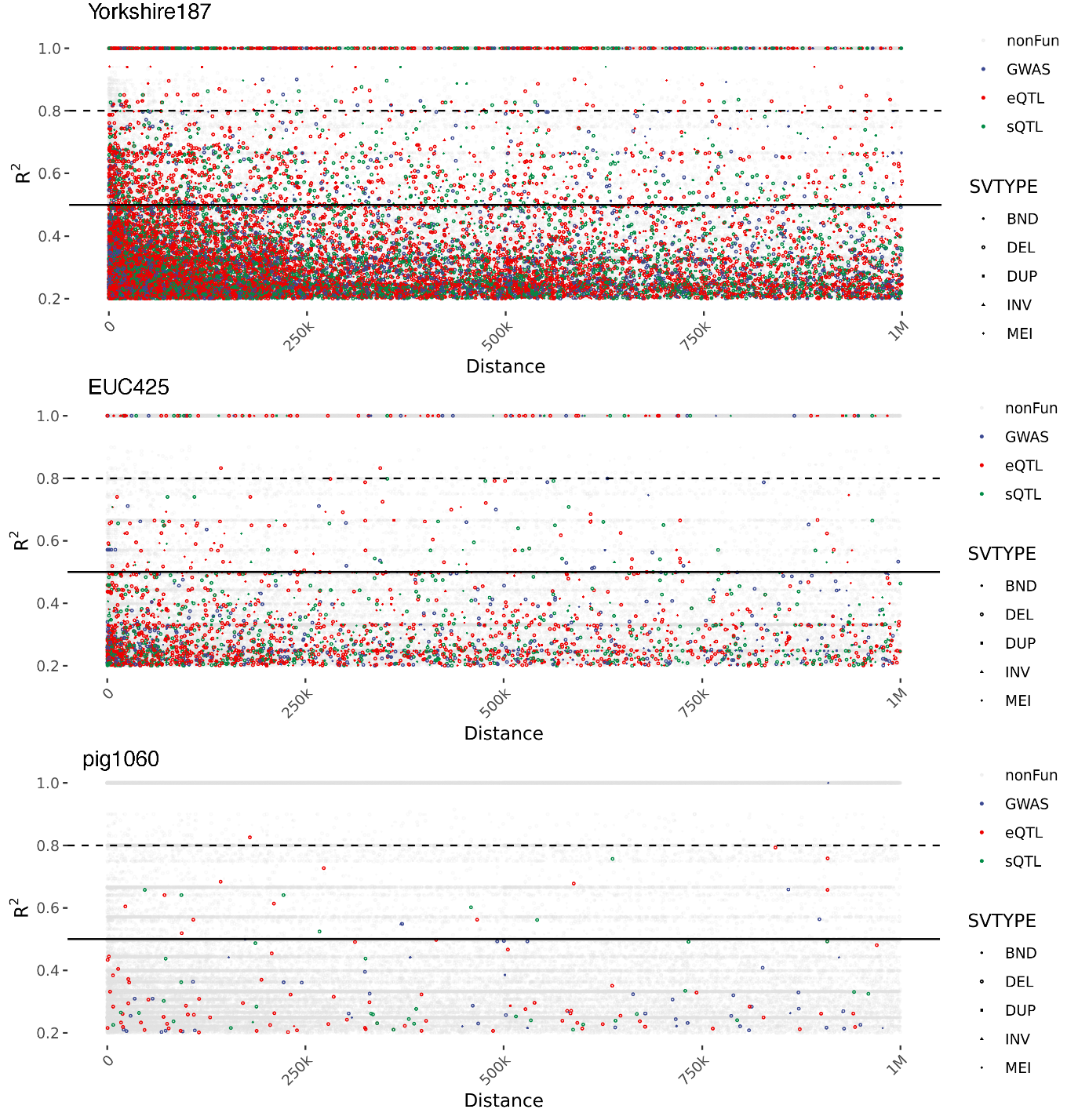


### Fig. S9. LD r^2^ at different genome distance for SVs and SNPs in Yorkshire, EUC and 1060 pigs.

Only displays the SNP with the highest LD r^2^ with the SV when this SV is associated with multiple linked SNPs.
