# Supplementary material for: Mapping and functional characterization of structural variation in 1060 pig genomes
Source: Genome Biol. 2024 May 7;25:116. doi: 10.1186/s13059-024-03253-3 (PMC11075355; doi:10.1186/s13059-024-03253-3)
Supplement: Supplementary file 4 — Additional file 4. [file 13059_2024_3253_MOESM4_ESM.docx]

**Review history**

**First round of review**

**Reviewer 1**

The authors report a comprehensive SV catalog based on the whole genome sequence of 1,060 pigs representing 101 breeds. It comprises 9.6% of the pig genome nonredundantly, including 42,487 deletions, 37,913 mobile element insertions, 3,308 duplications, 1,664 inversions, and 45,184 break ends. They explored the impacts of SVs on gene expression, functional elements, and complex traits of economic importance by systematically integrating them with expression quantitative trait loci (eQTLs) from 34 major tissues, chromatin states from 14 tissues, and sequence-based genome-wide association study (GWAS) signals of 14 complex traits from the Pig Genotype-Tissue Expression (PigGTEx) project, as part of FarmGTEx project. Variants in MYO5A and ABCG2 are nominated to be possible functional variants.

This is important work for providing a thorough - short read WGS-based - catalog of porcine structural variation. Thus, the data resource is likely to be of great impact (with the caveat that long read sequencing will add more detail in the future; but one cannot assume that is true in the near future for all pig breeds studied here). The sequencing details, a major technical aspect of this work, is essentially omitted. The work appears to be derived from a parent work that is cited as a preprint, and that work also does not have the sequencing details. It is difficult to assess the usefulness of the rest of this work attempting to nominate functional SVs/SV regions. The individual novel examples appear likely to have functional consequences, but the molecular mechanisms are not dissected experimentally, and it is not clear what the associated biological effects are or are likely to be for most.

The writing is generally acceptable, but the work is difficult to review due to lack of clarity about what was done and why. The Introduction is written well. The Results are written unevenly and are dense and are frustrating to read due to so many abbreviations and numbers. The Discussion is unevenly written (e.g., section "Selection signatures" could be improved). The work is very ambitious, and I commend the investigators for creating a single paper with so many computational resources investigated for relevance. The methods seem appropriate and rigorously done, but the work could use more rationale/justification and description as I am unsure of much of the functionality analysis.

It may be too late to change without major effort, but it seems the Results could have been streamlined by relying on figs. and tables; the Introduction could have included the rationales/approaches for the functional analyses; and the Discussion could have included a proper discussion of key functional analyses. Comparisons to domesticated species SV and human SV knowledge are completely and largely omitted, respectively. Given the massive analyses performed, I see that as a trade off with the functional genomic analyses of pig SVs. I could be convinced that is appropriate, except I'm not sure I understand the functional genomics findings.

Major comments
1. The use of outgroup should be clarified. As written, the distinction is between pigs and outgroup individuals. It should be clearer that "pig" is used to describe only Sus scrofa, and that the outgroup individuals are from other species in the family Suidae: some from the genus Sus but others from other genera.

2. The Abstract states "We also explored the impacts of SVs on gene expression, functional elements, and complex traits of economic importance". From reading the Methods, it seems SVs were not tested for such impact, but for enrichment in eQTL, QTL, and GWAS intervals <1 Mb. I don't recall seeing citations of previous studies using this approach. My impression is that this concept is difficult to interpret and assess for usefulness.

My understanding is that i) each of those existing resources were based on studies done in specific pig sub-populations such as Meishan or Duroc; ii) since the present work used WGS, then all the SVs are available in the context of haplotypes (maybe requiring imputation for 10-15X coverage used), which can be rare or common in all pigs or rare/common in individual sub-populations; and iii) that means the consideration should be whether eQTL/QTL/GWAS loci correspond to haplotypes containing SVs (followed by nomination of functional elements affected by the SV). For instance, an ideal example could be a Yorkshire-specific SV which corresponds to a Yorkshire GWAS locus.

If this work is only reporting positional correlation of SVs and eQTL/QTL/GWAS loci, the rationale should be provided and justified with references, and hopefully with examples of validated utility/interpretation.

3. Line 259 & Suppl Table 19: "There was no significant enrichment for [sequence-based] GWAS signals of 14 complex traits as compared to the global SV background (Fold changes range from 0.78 to 1.29, or the Bonferroni P > 0.01) (Figure S3c & Table S19)." This is among the most interesting analyses of the study: can SVs be nominated to explain GWAS loci. In my preceding comment, I note that was not thoroughly/precisely tested. However, beyond that issue, when I look at S3c and Table S19, there appear to be many significant findings (also very relevant to production traits like back fat thickness) - but the text I quoted here says "no significant enrichment". This again suggests the authors have a rationale that they assume readers know without being told.

The readers want to know if likely functional SVs were nominated to explain GWAS loci. If that was not done, the authors offer a test of SV enrichment at GWAS loci and say there was no significant enrichment but show fig/table that appears to indicate otherwise. As I suggest in the preceding comment, more rationale and explanation would be useful (e.g., difference between QTL and GWAS tests performed)

4. I randomly checked Suppl Table 21 and compared to the text: Line 301: "There were 571 SVs with a nonredundant length of 221.2 kb and overlapping with 216 genes, corresponding to the top 1% of FST values, which implies potential selection signatures between AS and EU ancestral groups (Table S21 &Table S22)."

I counted the genes for AS vs. EU in that table and found 515 genes rather than the 216 in the text.

5. Line 322 "A total of 20.1% (932/4632) DEGs mapped to 1646 SVs were detected… (Figure 4b &Table S28). Like preceding comments, I don't know if the claim is that DEGs were mapped to actual SVs (i.e., a specific haplotype) or SV interval (genome region that has an SV in any population).

6. Line 731: "We kept TWAS genes... if they were localized in SVs or overlapped with the flanking 5 kb regions of SVs on both sides… Based on SV-related DEGs between sub-population pairs, we selected e/sQTL-related genes from them to map GWAS loci using their concordant SNP ID. The traits representing significant correlation with GWAS position (P < 5e-8) were then confirmed by TWAS traits."

As in the preceding comments, it is unclear to me whether the SV is in the same haplotype and presumably a candidate functional variant for the specific mRNA expression detected by TWAS (or is it a positional correlation of a population specific SV and a gene with TWAS mapped expression in any of a group of members of same/related population?). Here there is mention of "SNP ID" but I am not sure I understand what that refers to and whether that is linking SNV and e/sQTL SNPs to determine they are the same haplotype.

7. Line 360: "We further checked the 303 bp DEL… MYO5A". I found this to be so confusing that I can't be sure there is anything interesting about the SV. There is a claim that the "DEL" is not a DEL but rather a SINE insertion. That is fine, except why is it presented as a DEL and not reclassified as an MEI?

We are told the MEI "broke MYO5A-201 into the two disconnected UTRs, leading to the creation of two short transcripts of MYO5A-202 and MYO5A-206 (Figure 5a). The decrease in full-length mRNA level was associated with reduced protein level and deficient melanosome transport, suggesting a potential mechanistic link between the MYO5A mutations and the dilute color phenotype."

Seems the way to say that is more like the following: Skin RNAseq data showed that, compared to WT, MEI SV carriers expressed #% less of isoform 201 and additionally expressed isoforms 202/206 which are not present in WT (Fig. #).

That is, we are not told specifically what the evidence is and what is the level of effect - is it a 10%, 20%, 40% or what level of reduced expression. Similarly, re: "dilute color phenotype": Table S32 shows something like 30 breeds fixed for non-deletion SV and 50 fixed for deletion SV - seems to me the authors could have mentioned if one group has lighter and the other darker coat generally. Instead, they write "suggesting a potential mechanistic link between the MYO5A mutations and the dilute color phenotype".

8. Line 399: "We detected a strong-effect copy number gain (Yorkshire-specific DUP of 55,665 bp, 8:130924619-130980283) within ABCG2, which could potentially down-regulate its expression in blood, embryo, and uterus in Yorkshire as compared to Duroc."

What is meant by "strong-effect"? When one speaks of CNV, effects can reflect the number of copies gained or biological effects, including gene expression or physiological impact. If it is a many-copy number gain, it seems straightforward to say that, and, if there is an estimate of minimum or most likely copy number, that is useful to know.

9. Lines 403 and 1126/"Fig 6b. Illustration of the genomic region for copy number gain of ABCG2." This figure is difficult for me to interpret because it is one genomic interval illustrating two structurally variant versions. For the gene/mRNA and TAD structure, it would be simpler to show both states separately so they/the evidence can be visually compared. The panels at the very bottom have very faint data that was impossible for me to interpret visually.

10. Discussion: Line 441 "Although short-read sequencing presents challenges in accurate identification of SVs, it is currently the most practical way to assess SV diversity". It would be useful for readers to understand similar work going forward will probably use long read methods (or combination of the two). PacBio is used for validation purposes here. The authors could say in one or two sentences what that transition will be like (as it has been done in other species).

11. Discussion: Line 493: Similarly to Results of the same: The MYO5A discussion is confusing and - if I understand correctly - unconvincing. It is first discussed as a deletion, at the end of this MYO5A section, the authors say they changed their mind - that it is not a deletion in deletion SV carriers but a likely mobile element insertion in non-carriers. Again, Table S32 shows something like 30 breeds fixed for non-deletion SV and 50 fixed for deletion SV - how can there be no mention of coat color/pattern differences between the two groups? As a result, one of a total of only two SV-gene biological findings mentioned in the Abstract is unsatisfying for both the molecular genetics of the SV and the candidate relevant-physiology (and I am confused as to why that is since they have RNAseq data to report the quantitative and qualitative effects and presumably have the coat phenotype data for all the breeds used).

12. Methods: Line 528: "A total of 1,208 WGS data with at least 10× coverage were retrieved from the data collected for the FarmGTEx project for pigs (PigGTEx) [33]." As far as I can tell, the work refers to the genetic data it is based on as "short read sequence" and "paired end", but I couldn't find a description of any other detail (read length, sequencing platform, etc.). Ref. 33 is a bioRxiv preprint: it states that details of the WGS are in Suppl. Table 2, but I could not find such a table on that site, and could not find description of the WGS in the pre-print.

13. Given the species studied and, say, George Liu's participation (who studies cattle CNV), I was disappointed there was no discussion of the domestication angle - e.g., for the domesticated species with a body of knowledge of SVs, how do they compare for numbers of SVs or biochemical pathways affected, and signatures of selection? Similarly, it seems likely there is reason to use the deep knowledge of human SVs to glean insights, but this doesn't appear to have been done thoroughly. I understand this was likely because the present approach was to push the limits of all available pig resources - which are many. However, I found that effort to be so confusing that I didn't know what firm knowledge was gained (i.e., as asked several times above, is it all positional correlations of SVs and phenomena in any pig population, or is it SV haplotypes associated with specific traits in specific pig populations).

Minor comments
1. Suppl figs. would be improved by providing fig. # and legends on the same PDF
2. Abstract: first sentence: "large regions of DNA sequence" presumably was intended to mean large "amounts" vs large "regions". Also line 99: "This form of variation involves larger segments of the genome": larger "segments" should be a larger "proportion"
3. Abstract: third sentence: "It covers 9.6% of the pig genome": use of "covers" may confuse some readers vs. SVs "comprise" 9.6%
4. Line 102: "observed to range from 50 base pairs (bp) to 5 megabase pairs (Mb)". I think "observed" here is intended to mean "defined as" 50 bp or more (and generally refers to up to 5 Mb but has no upper limit)
5. Line 103: "The large size of SV increases the likelihood that SV might impact gene expression and function". I think that intends to say "With increasing size of SVs, the likelihood of impacting genes and their expression increases"
6. Line 151: "we totally discovered and genotyped 130,556" should be "we discovered and genotyped a total of 130,556"
7. Line 155: "The lengths of DEL, MEI, DUP, and INV were 96.3, 16.6, 61.3, and 59.6 Mb, respectively, covering 9.6%" should be something like "The sum of the lengths of…, constituting 9.6%"
8. Line 171: "A large part" should be "A large portion"
9. Line 203: "enriched in 23 Gene Ontology (GO) terms, such as membrane protein complex, cell junction, kinase activity, and ATP-dependent activity (Table S7)." It is not clear why three of the four chosen are among the weakest by P adj (marginally significant). I believe mentioning the top four as follows makes more sense: phosphorylation (4 of 5 top terms; starting with P adj= 2.38E-06), exopeptidase activity, ion transmembrane transporter activity, and cytoskeletal protein binding
10. Line 204: Similarly to preceding comment: Table S8 has 8 significant GO terms, but 5 are phosphorylation/kinase terms and 3 are anion/chloride channels/transporters. Thus, it is straightforward to concisely describe the results more thoroughly and clearly
11. Line 204: "Among them, genes overlapped singleton SVs" should say "gene-overlapping singleton SVs"
12. Line 234: "Of them, the highest partitions" should be "Of those…" and, I believe the intention is to continue "… the highest proportions". Line 305 also has "Of them" which should be Of those
13. Line 410, "Intriguingly, this DUP leaped over its neighbor gene PKD2". It is not clear what "DUP leaped over its neighbor" was intended to mean. In Line 509 in Discussion, it is clear it refers to the SV not affecting the expression of the closest downstream gene but influencing that of the next one after that
14. Line 465: "The occurrence of MEIs… indicates that SVs may be critical agents for gene expression pleiotropy that is often observed in stress-responsive genes." Please provide ref. for stress-responsive genes statement

**Reviewer 2**

The paper by Yang et al is certainly comprehensive and represents the largest and most complete - also in terms of pig diversity considered - to date. In fact it may be a landmark paper now that we are moving on to the next phase of variation analyses using long reads and pangenomes. For pigs, this paper may represent the best that could be done using short reads.

So, I have no doubt the paper has potential to be cited a lot. Still I am somewhat conflicted since there is relatively little biological insight coming from the paper despite the deep integration of structural variation analysis and functional data. Authors do provide lots of results but it is not always very clear what the significance is, and it is also not always easy to take in the results to begin with. Figure 4b is a good example of such a presentation of results - very interesting, but where to begin with reading it and what exactly to take home from it? Given the broad readership of the journal this is perhaps a concern, although I leave that to the editor to decide.

The paper has lots of value as a resource paper - again, it could be seen as a landmark paper. But then it is very important that ALL data is easily accessible publicly. Part of the data is already in public databases, but - maybe I've missed it - in most cases there are no database identifiers noted in the Sup tables. I'm also concerned about the functional data sources - expression data especially - since unclear to me that it can be retrieved from a public database. For the paper to really have value it is important that all these data are not only in public databases but also easily retrievable with no gatekeeping system.

Lastly I'm very concerned about the layout of most of the figures. Figures are cramped and often not easy to comprehend. I'm not sure how they would end up in print.
Some smaller points:

L 116-121 In the discussion on defining CNVs/large with short read sequencing: long read sequencing such as Nanopore sequencing makes such issues largely obsolete. It is ok to discuss this but this should probably be mentioned at least.

L 131: "Interbred hybridization", should probably better be "crossbreeding".

L 528: "A total of 1,208 WGS data" - "data" should be "datasets"? For clarity, "WGS data" should be replaced with "whole genome short-read sequence datasets".
L 575: Provide list of 'bad bins'.

L594: NGS? Short read sequencing?

Breakend (BND) poorly defined in the paper. Since this is a term that is not unambiguously used in the literature, authors should a) define it properly and unambiguously, and b) be explicit which part of the analysis particularly supports BND.

KIT example is confusing; maybe because it seems that Fig S5 is truncated (?). KIT structural variations have been well described from Yorkshire and Pietrain, so I'm wondering why these were not included, while Duroc is (although many Duroc nowadays are in fact white, traditionally they lack the SV).

**Authors’ response to reviewers**

**Comments from Reviewers**:

**Reviewer #1:**

**General comments**: The authors report a comprehensive SV catalog based on the whole genome sequence of 1,060 pigs representing 101 breeds. It comprises 9.6% of the pig genome nonredundantly, including 42,487 deletions, 37,913 mobile element insertions, 3,308 duplications, 1,664 inversions, and 45,184 break ends. They explored the impacts of SVs on gene expression, functional elements, and complex traits of economic importance by systematically integrating them with expression quantitative trait loci (eQTLs) from 34 major tissues, chromatin states from 14 tissues, and sequence-based genome-wide association study (GWAS) signals of 14 complex traits from the Pig Genotype-Tissue Expression (PigGTEx) project, as part of FarmGTEx project. Variants in MYO5A and ABCG2 are nominated to be possible functional variants.

This is important work for providing a thorough - short read WGS-based - catalog of porcine structural variation. Thus, the data resource is likely to be of great impact (with the caveat that long read sequencing will add more detail in the future; but one cannot assume that is true in the near future for all pig breeds studied here). The sequencing details, a major technical aspect of this work, is essentially omitted. The work appears to be derived from a parent work that is cited as a preprint, and that work also does not have the sequencing details.

It is difficult to assess the usefulness of the rest of this work attempting to nominate functional SVs/SV regions. The individual novel examples appear likely to have functional consequences, but the molecular mechanisms are not dissected experimentally, and it is not clear what the associated biological effects are or are likely to be for most.

The writing is generally acceptable, but the work is difficult to review due to lack of clarity about what was done and why. The Introduction is written well. The Results are written unevenly and are dense and are frustrating to read due to so many abbreviations and numbers. The Discussion is unevenly written (e.g., section "Selection signatures" could be improved).

The work is very ambitious, and I commend the investigators for creating a single paper with so many computational resources investigated for relevance. The methods seem appropriate and rigorously done, but the work could use more rationale/justification and description as I am unsure of much of the functionality analysis. It may be too late to change without major effort, but it seems the Results could have been streamlined by relying on figs. and tables; the Introduction could have included the rationales/approaches for the functional analyses; and the Discussion could have included a proper discussion of key functional analyses.

Comparisons to domesticated species SV and human SV knowledge are completely and largely omitted, respectively. Given the massive analyses performed, I see that as a trade off with the functional genomic analyses of pig SVs. I could be convinced that is appropriate, except I'm not sure I understand the functional genomics findings.

AU: Thank you very much for these positive and very constructive comments and suggestions on the manuscript. The details of all the 1,060 WGS data being analyzed in this study are provided in the revised Supplementary Table S43. As indicated by the reviewer, the main focus of this study was to provide a comprehensive SV catalog across global pig breeds using extensive whole-genome sequences followed by studying their potential functional impacts on pig molecular and complex traits through examining the results of PigGTEx. Although experimental validation of particular impacts falls beyond the current scope of this work, the identified SV candidates could serve as valuable resources for designing future functional investigations. We have carefully revised the manuscript, and provided detailed responses to each comment below, including the main efforts addressing the comments from reviewers:

1. To make the functional enrichment analysis of SV regions easily understood by the readers as suggested by the reviewer, we carefully introduced its rationales and approaches in the revised Introduction, improved the relevant results, and added more discussions. Furthermore, we conducted additional analysis for the *MYO5A* gene example, including re-assembling all its potential transcripts, comparing their splicing patterns and expression levels across tissues, and associating them with coat color in Asian and European pigs (L360-384).
2. To investigate whether SVs and adjacent functional SNPs (e.g., eQTL, sQTL, and trait-associated variants from GWAS) are genetically linked at the population level, we systematically explored their LD patterns within and across pig breeds (L436-512).
3. As suggested, we substantially improved the writing of the manuscript, particularly by adding rationales of functional enrichment analysis and LD analysis of SVs in the revised Introduction and streamlining Results by relying on figs. and tables, as well as discussing key functional analysis and improving the Discussion sections like "Selection signatures".
4. Added brief discussions on comparative SV analyses among humans, pigs, and cattle, including the shared and lineage-specific events and associated pathways (L604-622).

**Major comments**

1. The use of outgroup should be clarified. As written, the distinction is between pigs and outgroup individuals. It should be clearer that "pig" is used to describe only Sus scrofa, and that the outgroup individuals are from other species in the family Suidae: some from the genus Sus but others from other genera.

AU: Revised. We have added the description of pig (*Sus scrofa*) and outgroups (i.e., other species in the family Suidae, including *Babyrousa babyrussa*, *Phacochoerus africanus*, *Potamochoerus porcus*, *Potomochoerus porcus*, *Potomochoerus larvatus*, *Porcula salvania*, *Sus Celebensis*, *Sus Verrucosus*, *Sus Barbatus*, *Sus Cebifrons*, and Sumatr) in Methods L647-649.

2. The Abstract states "We also explored the impacts of SVs on gene expression, functional elements, and complex traits of economic importance". From reading the Methods, it seems SVs were not tested for such impact, but for enrichment in eQTL, QTL, and GWAS intervals ≤1 Mb. I don't recall seeing citations of previous studies using this approach. My impression is that this concept is difficult to interpret and assess for usefulness. My understanding is that i) each of those existing resources were based on studies done in specific pig sub-populations such as Meishan or Duroc; ii) since the present work used WGS, then all the SVs are available in the context of haplotypes (maybe requiring imputation for 10-15X coverage used), which can be rare or common in all pigs or rare/common in individual sub-populations; and iii) that means the consideration should be whether eQTL/QTL/GWAS loci correspond to haplotypes containing SVs (followed by nomination of functional elements affected by the SV). For instance, an ideal example could be a Yorkshire-specific SV which corresponds to a Yorkshire GWAS locus.

If this work is only reporting positional correlation of SVs and eQTL/QTL/GWAS loci, the rationale should be provided and justified with references, and hopefully with examples of validated utility/interpretation.

AU: We divided our responses into the below subsections.

**2.1 Summary:** Thanks for the suggestions, and we agree with the reviewer. Following the reviewer's suggestion to effectively link SVs and functional variants (e.g., sQTL) genetically rather than purely based on genomic positions, we have carefully assessed the genetic relationship between SVs and their neighboring functional variants (lead e/sQTL and GWAS lead SNP) by computing their linkage disequilibrium (LD) within and across various pig breeds/populations (details see below). In general, we considered LD r^2^ ≥ 0.5 and 0.8 as tagged and highly tagged, respectively. **In the revised manuscript, we have replaced position-based enrichment analyses with the new LD-based analyses** to explore the potential impact of SV on molecular and complex phenotypes. These LD-based analyses have been routinely used in the most recent human short read SV papers (Collins et al., gnomAD and Abel et al. NHGRI’s CCDG) [1, 2], to test the relationship between e/sQTL and GWAS loci. Please see Chiang et al. and Kojima et al. for details [3, 4]. We thus have rephrased this sentence in the Abstract as below: “…we also conducted an initial exploration into how SVs might impact gene expression, functional elements, and phenotype traits on the population level. This was achieved by examining their linkage disequilibrium with regulatory variants (e/sQTLs-e/sGenes, GWAS loci), as well as gene-trait associations (TWAS) retrieved from the Pig Genotype-Tissue Expression (PigGTEx) project”.

**2.2 Fold enrichment**: Because physical distance is a good indicator of genetic linkage, fold enrichment through a position-based overlap test is also widely used to detect the relative enrichment or under-representation of a specific feature within a dataset compared to a background or control dataset [1, 2]. However, as mentioned above, following the reviewer’s suggestion, in the revised manuscript, we have replaced fold enrichment tests with the new LD-based analyses for eQTLs, sQTLs, and GWAS loci. The only remaining fold enrichment tests were for QTL and DEG, in which we consistently enforced a requirement of at least 1 bp overlap (L770). The “1 Mb cutoff” was only used in two specific instances: (1) to eliminate large trait-QTLs that were imprecisely mapped in pigs, and (2) to report all pairwise LD r^2^ values within a 1 Mb genomic distance.

**2.3 Pig populations**: We conducted extensive investigations to depict the LD between SVs and adjacent variants. In our PigGTEx project, we processed 9,530 RNA-sequencing and 1,602 whole-genome sequencing samples from pigs [5]. This work complements the PigGTEx project and serves as a valuable SV resource for global pig populations. Our primary goal is to offer a comprehensive catalog of SV within worldwide pig populations, providing overall statistics for functional elements associated with SVs and the potential functions they might influence.

**2.4 Limitations:** Due to the lack of long-read WGS and matched RNA-seq data, attempting to directly impute SVs using SNPs based on a single haplotype within selected individuals or breeds proved to be very challenging, if not impossible. Therefore, evaluating their impacts on RNA-seq or complex traits was difficult. To address this question, we needed to rely on calculations of the LD conducted on the population level. Unlike short reads, long reads can connect two or more SVs and SNPs using a single sequencing read over a considerable distance. In the future, we will be able to directly address this genetic relationship between SNPs and SVs using long-read sequencing. As a part of that initiative, we are generating pig pangenome graph assemblies and haplotype-resolved SV reference panels derived from long-read sequencing of multiple breeds [personal communications, Miao et al., in processing].

For the comment “since the present work used WGS, then all the SVs are available in the context of haplotypes”, it’s important to acknowledge the limitations surrounding SV genotyping, phasing, and imputation using short-read data, which have been reviewed extensively before [1, 2, 6, 7]. These limitations stem from (1) short read sequencing; (2) the lack of a direct alignment-based SV call strategy; and (3) the constraint posed by using a single linear haplotype reference genome like Sscrofa11.1. Most short-read SV detection methods rely on indirect inferences from unusually mapped reads to call SVs. Short reads also often fail to span the break ends of SVs, contributing to the difficulty in associating SVs with a specific haplotype. DELs are relatively easy to genotype as 0/1 (impacting one haplotype) or 1/1 (total deletion, affecting two haplotypes), similar to SNP genotyping. However, for DUPs and BNDs, it becomes challenging to ascertain which haplotype is involved and the exact number of sequence duplications. For example, 1/2 and 0/3 cannot be told apart on the sequence level. We thought about the strategy of phasing haplotypes using SNP information and subsequently integrating SVs into these haplotypes, as shown in [8]. Their SV set was called from the PacBio HiFi long-read sequencing data from 10 Chinese cattle. However, currently, there was no readily available population-scale long-read data or pangenome reference assembly for our analysis, despite a few pig pangenome papers [9].

**2.5 Genetic linkage between SV and functional SNP**

We first computed the LD for the combined genotype matrix of SVs and SNPs using PLINK (v1.90b6.21). We reported all pairwise r^2^ values if they were larger than 0 within a 1 Mb genomic distance. We observed LD of each SV type with SNPs followed a similar decay pattern over the genomic distance, like SNP-SNP pairs and each SV type with itself (**Figure S7**).

Notably, at the 200kb distance, SNP-SNP pairs exhibited the highest r^2^ values, with a median of ~0.18 for Yorkshire (**Figure S7a**). DEL-DEL pairs displayed LD levels closest to SNP-SNP pairs, producing a median r^2^ value of ~0.16 (the top panel of **Figure S7b**), followed by MEI-MEI pairs with a median r^2^ value of ~0.10. With expanded sample sizes representing a more complex genetic background, the LD of all pairs displayed a rapid decline.


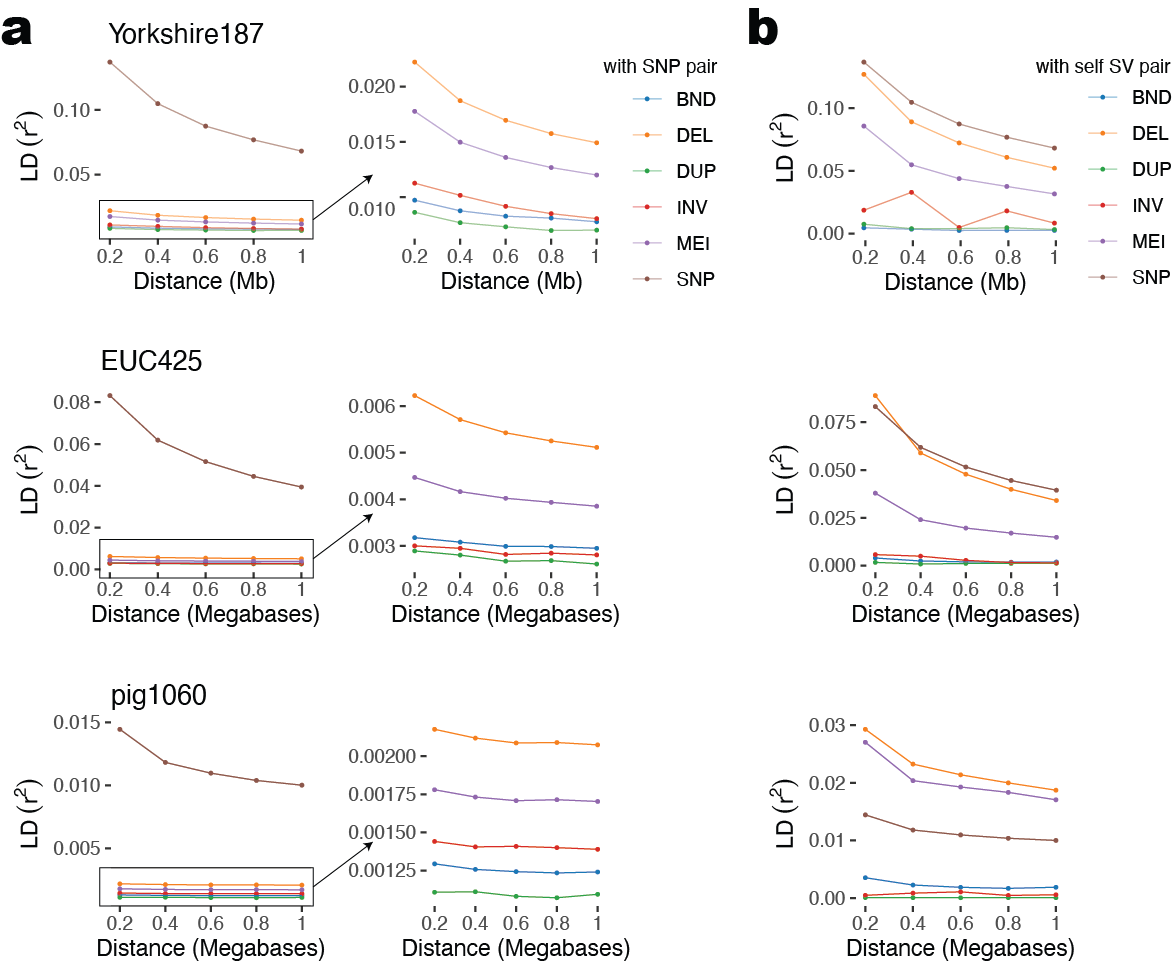


Figure S7. LD decay for SVs and SNPs in 1060 pigs, 425 EUC pigs, and 187 Yorkshire pigs.

Generated using PLINK --r2 with a threshold of 0, indicating the reporting of all pairs of SNPs and SVs within a 1 Mb distance (--ld-window-kb 1000). (a) The LD decay of each type of SV with SNP pairs. (b) The LD decay of SV and SV pairs within each SV type.

**2.6. SNP-tagged SVs**

Besides the above general trends, we also summarized the statistics of the LD for all SV-SNP pairs and all SV-functional SNP pairs in this section. We found that more than half (66.45%, 52.96%, and 50.64%) of SVs exhibited LD r^2^ ≥ 0.2 (defined as “linked”) with at least one SNP for Yorkshire, EUC and 1060 pigs. Within these three groups, 37.38%, 24.46%, and 22.76% of SVs were found to have their tagged SNPs (r^2^ ≥ 0.5), respectively. Additionally, 31.75%, 21.88%, and 19.22% of SVs demonstrated highly tagged linkage (r^2^ ≥ 0.8) with their flanking SNPs (**Table S33**).

**2.7. SV imputation and LD block.**

We tried to assess SV imputation accuracy by utilizing Eagle (v2.4.1) and Minimac (v4.1.3). Employing a 10-fold cross-validation strategy, we randomly selected 1/10 of the samples as the validation dataset. Within these validating samples, we randomly marked 10%, 20%, 50%, and 80% of the SV genotypes as missing values (./.). The remaining 9/10 samples were used as training data for Eagle2 to create a reference panel. We then used Minimac4 to impute the missing genotypes in the validation set based on the training reference panel. Then, we compared the raw genotypes before setting them as missing with the imputed genotypes. The accuracy was calculated by the number of match genotypes divided by the number of set missing genotypes. However, these attempts resulted in low imputation accuracies (<70%) and unreliable results. These limitations might stem from the constraints of short-read sequencing lengths and the absence of robust methods for detecting, classifying, and genotyping SVs. Consequently, we decided to leave them out for this manuscript. In our future work, we plan to explore long-read strategies to investigate SV imputation accuracy and the possibility of using neighboring SV-tagged SNPs from the same long read as markers. Additionally, we will explore pangenome graph assembly, which holds promise for more accurate and haplotype-resolved SV genotyping.

### 3. Line 259 & Suppl Table 19: "There was no significant enrichment for [sequence-based] GWAS signals of 14 complex traits as compared to the global SV background (Fold changes range from 0.78 to 1.29, or the Bonferroni P > 0.01) (Figure S3c & Table S19)." This is among the most interesting analyses of the study: can SVs be nominated to explain GWAS loci. In my preceding comment, I note that was not thoroughly/precisely tested. However, beyond that issue, when I look at S3c and Table S19, there appear to be many significant findings (also very relevant to production traits like back fat thickness) - but the text I quoted here says "no significant enrichment". This again suggests the authors have a rationale that they assume readers know without being told. The readers want to know if likely functional SVs were nominated to explain GWAS loci. If that was not done, the authors offer a test of SV enrichment at GWAS loci and say there was no significant enrichment but show fig/table that appears to indicate otherwise. As I suggest in the preceding comment, more rationale and explanation would be useful (e.g., difference between QTL and GWAS tests performed)

AU: As described in 2.1 above, we have replaced position-based enrichment analysis with this new LD-based analysis for GWAS loci. As described recently for human MEIs [4], we considered LD with r^2^ larger than 0.5 and 0.8 as tagged and highly tagged to find examples of GWAS trait-associated SVs. We then focused on local LDs and plotted the distribution of r^2^ values for SV-SNP pairs as physical distance increases (Figure S8).


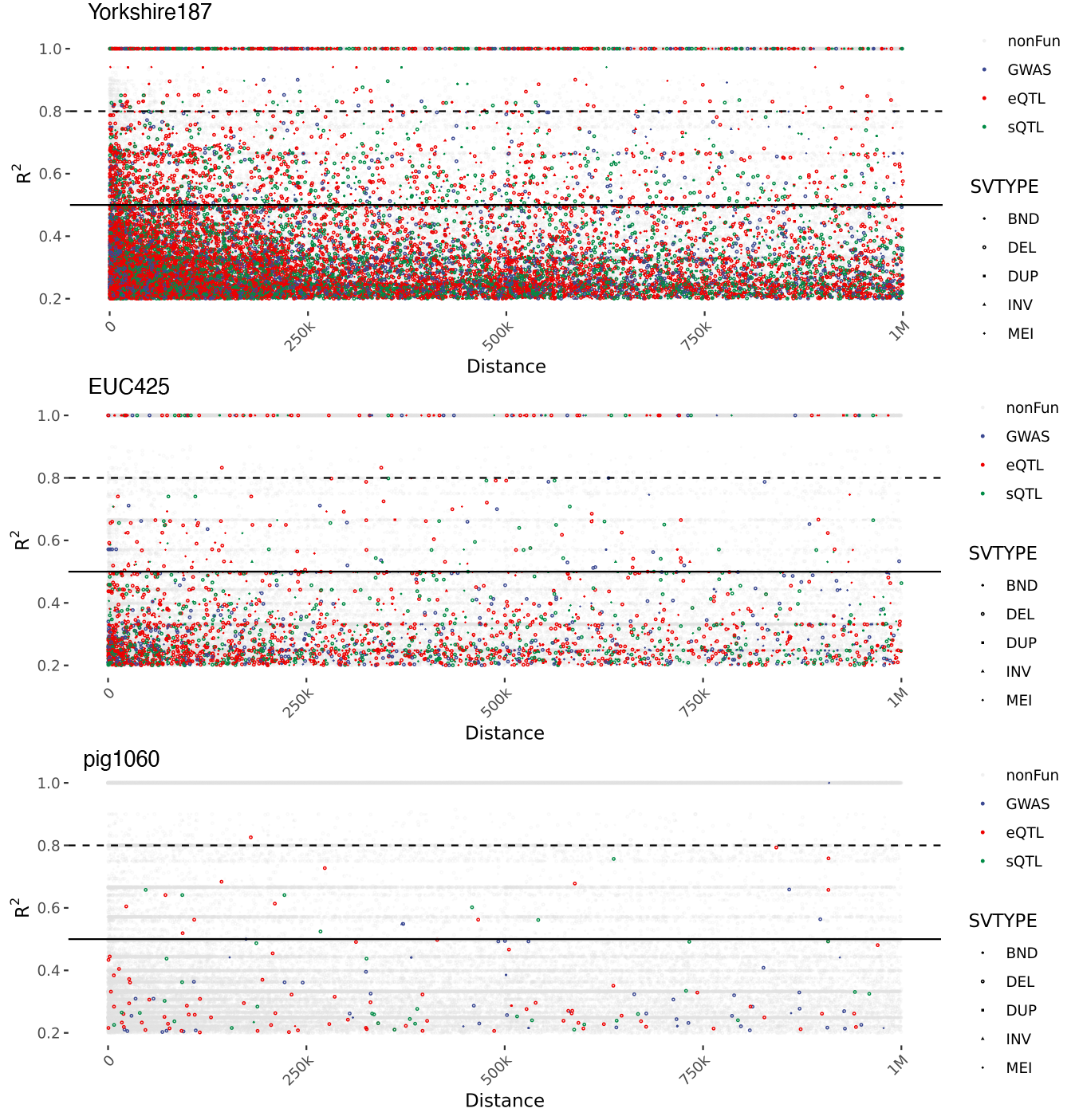


**Figure S8. LD r^2^ at different genomic distances for SVs and SNPs in Yorkshire, EUC, and 1060 pigs.** Only displays the SNP with the highest LD r2 with the SV when this SV is associated with multiple linked SNPs.

We discovered an intriguing example of the “2:9513511-9513561:DEL” deletion linked with the leading eQTL “2:9621709C>T”, an SNP significantly associated with the loin muscle area trait by GWAS. This SNP was also validated by its corresponding eGene - *FADS3* (Fatty Acid Desaturase 3) in TWAS, which further confirmed a significant association between *FADS3* and the loin muscle area in embryo tissue (**Table S35**). This 51-bp deletion “2:9513511-9513561:DEL” directly overlaps strong enhancer regions “2:9513400-9513600” in muscle, “2:9513400-9513800” in the ileum and jejunum, as well as various other types of enhancers in 13 other tissues (**Figure 7** and **Table S37**). Additionally, this deletion “2:9513511-9513561:DEL” is linked to 3,584 SNPs (r^2^ ≥ 0.2, see Methods for definitions of “linked”, “tagged”, and “highly-tagged”), including 126 eQTLs, 30 sQTLs, and 10 GWAS loci, which have the potential to regulate the expression of 51 genes, splice 20 isoforms, and influence 3 traits (average backfat thickness, lean meat percentage, and loin muscle area) (**Table S38**). The 3,584 SV-linked SNPs span a near 2 Mb genomic region ranging from 8,525,445 to 10,513,127 (a total of 1,987,683 bp). Moreover, we identified 66 QTL regions located in this SV-SNP linked region, including obesity index, dihomo-gamma-linolenic acid content, arachidonic acid to dihomo-gamma-linolenic acid ratio, etc. (sorted by the distance to “2:9513511-9513561:DEL”) (**Table S39**). The *FADS3* gene belongs to the fatty acid desaturase gene family, which has been identified as a strong candidate gene for lipid metabolism in pig muscle and backfat [10, 11]. Furthermore, 119 tagged SVs (r^2^ ≥ 0.5) were linked to 595 functional SNPs, 318 e/sGenes and 8 GWAS traits for 425 EUC pigs, and 323 highly-tagged SVs (r^2^ ≥ 0.8) were linked to 2,003 SNPs involved in the expression of 722 genes, splicing of 244 genes, and 8 GWAS traits for 187 Yorkshire pigs (**Tables S40 & S41**). Additionally, a high *F_ST_* value was observed between Yorkshire and Duroc pigs. These findings collectively suggest that the 51 bp DEL might be involved in regulating *FADS3* by modulating its enhancer in muscle tissue.

### 4. I randomly checked Suppl Table 21 and compared to the text: Line 301: "There were 571 SVs with a nonredundant length of 221.2 kb and overlapping with 216 genes, corresponding to the top 1% of FST values, which implies potential selection signatures between AS and EU ancestral groups (Table S21 &Table S22)."

I counted the genes for AS vs. EU in that table and found 515 genes rather than the 216 in the text.

AU: We are sorry for the errors. We have checked and corrected the numbers reported in the manuscript. Since the position-based enrichment analysis of e/sQTL overlapping SV was replaced and we now only report the location information of SV in newly revised Table S17, we deleted the columns for Ensemble genes and gene symbols.

### 5. Line 322 "A total of 20.1% (932/4632) DEGs mapped to 1646 SVs were detected… (Figure 4b &Table S28). Like preceding comments, I don't know if the claim is that **DEGs** were mapped to **actual SVs** (i.e., a specific haplotype) or **SV interval** (genome region that has an SV in any population).

AU: As mentioned before, we utilized SV intervals for the DEG overlap tests. Following the DEG calculations between the AS and EU groups for each tissue, we identified AS-specific, EU-specific, and high *F_ST_* SVs between the AS and EU groups. We retained SVs that either overlapped with or were in proximity to DEGs—directly overlapping with the gene body or with enhancer/promoter regions ranging from 5 kb upstream to 5 kb downstream of the gene body. This highlights the potential impacts of SVs on DEGs when they physically overlap with gene bodies or their enhancer/promoter regions. Typically, a 5kb genomic distance is generally accepted to be enriched for local regulatory elements.

To detect SV-related DEGs, we need to phase SV with specific haplotypes. However, due to limitations in our dataset—specifically, the insufficient number of samples with corresponding WGS (Whole Genome Sequencing) and RNA-seq data, along with the low accuracy of SV imputation mentioned in Section 2.7—we were unable to impute SVs using transcriptome SNPs. Consequently, conducting haplotype-based DEG analysis was not feasible with our current data.

### 6. Line 731: “We kept TWAS genes... if they were localized in SVs or overlapped with the flanking 5 kb regions of SVs on both sides… Based on SV-related DEGs between sub-population pairs, we selected e/sGenes from them to map GWAS loci using their concordant SNP ID. The traits representing significant correlation with GWAS position (P ≤ 5e-8) were then confirmed by TWAS traits.”

As in the preceding comments, it is unclear to me whether the SV is in the same haplotype and presumably a candidate functional variant for the specific mRNA expression detected by TWAS (or is it a positional correlation of a population specific SV and a gene with TWAS mapped expression in any of a group of members of same/related population?). Here there is mention of “SNP ID” but I am not sure I understand what that refers to and whether that is linking SV and e/sQTL SNPs to determine they are the same haplotype.

AU: TWAS examines the association between gene expression and phenotype traits, establishing pairs of genes and phenotype traits. GWAS investigates the association between SNP genotypes and phenotypes, forming pairs of SNPs and phenotype traits. The e/sQTL mapping explores SNP genotypes and molecular phenotypes like transcriptions, generating pairs of e/sQTLs and their corresponding eGenes. We combined three sets of information—gene by gene, SNP by SNP, and trait by trait—to establish interaction networks to illustrate how SNPs (e/sQTLs) regulate genes and impact phenotype traits. As suggested by Reviewer 1, we have replaced the position-based enrichment analysis with this new LD-based analysis in the revised manuscript. We then incorporated SV data into these interaction networks to depict the regulatory role of e/sQTLs on gene expression and splicing, as shown for the 2Mb region.

### 7. Line 360: “We further checked the 303 bp DEL… MYO5A”. I found this to be so confusing that I can’t be sure there is anything interesting about the SV. There is a claim that the “DEL” is not a DEL but rather a SINE insertion. That is fine, except why is it presented as a DEL and not reclassified as an MEI?

AU: The selection of a reference genome can introduce biases that significantly affect the interpretation of SV results. For instance, instances of novel MEIs in the reference genome were frequently identified as deletions in studied animals lacking such MEIs. However, because it is almost impossible to remove inserted MEIs from the genomes without leaving any mark, these newly discovered MEIs/deletions should be classified as insertions in the reference genome instead.

We are told the MEI “broke MYO5A-201 into the two disconnected UTRs, leading to the creation of two short transcripts of MYO5A-202 and MYO5A-206 (Figure 5a). The decrease in full-length mRNA level was associated with reduced protein level and deficient melanosome transport, suggesting a potential mechanistic link between the MYO5A mutations and the dilute color phenotype.”

Seems the way to say that is more like the following: Skin RNAseq data showed that, compared to WT, MEI SV carriers expressed less of isoform 201 and additionally expressed isoforms 202/206 which are not present in WT (Fig. #).

That is, we are not told specifically what the evidence is and what is the level of effect – is it a 10%, 20%, 40% or what level of reduced expression. Similarly, re: “dilute color phenotype”: Table S32 shows something like 30 breeds fixed for non-deletion SV and 50 fixed for deletion SV – seems to me the authors could have mentioned if one group has lighter and the other darker coat generally. Instead, they write “suggesting a potential mechanistic link between the MYO5A mutations and the dilute color phenotype”.

AU: We further checked the 303 bp DEL (1:119154722-119155024) occurred in the second intron of a transcript of gene *MYO5A* (MYO5A-201) (**Table 2**), and there were many reads flanking it from RNA-seq in AS pigs (**Figure 5a-b**). This DEL in AS corresponded perfectly with a young pig SINE/Pre0_ss of 243 bp and a ~64 bp tandem repeat ploy-A tail, which were flanked by a signature of target site duplication of 15 bp sequence within their 20bp flanking regions. The observed allele frequency of DEL was near 0 % for EUW pigs and near 100% for ASW pigs, and this was true for most EUD and EUC pigs, which did not have this DEL as compared to ASD pigs (**Figure 5d** **and Table S28**). As the selection of a reference genome (a European Duroc pig) can affect the interpretation of SV results, MEIs in the reference genome were frequently identified as deletions in studied animals lacking such MEIs. Also, because it is almost impossible to remove inserted MEIs from the genomes without leaving any mark, these discovered deletions should be classified as MEIs in the European pigs instead. We thus hypothesize that this SINE/Pre0 MEI was specifically inserted into EU breeds recently. We first screened this SINE/Pre0 MEI and found three candidate motif types that may mediate the transcription factor binding (**Figure 5c**). We then reassembled the *MYO5A* gene transcripts using StringTie (v2.1.7). Wuzhishan and Bamaxiang pigs with dark patch on white skin color made up the samples for the AS group, while Yorkshire and Composite pigs with white color phenotype made up the samples for EU pigs. A total of 20 transcripts were obtained as shown in **Figure S5a**, including 6 known transcripts (MYO5A-201 to 206) and 14 novel and predicted transcripts (beginning with MSTRG.8280). We did not detect significant expressions in skin for 3 of them (MSTRG.8280.4, MSTRG.8280.7, and MSTRG.8280.10). For the rest, we found AS (Wuzhishan and Bamaxiang) pigs had at least 2 times higher expression than EU (Yorkshire and Composite) pigs for 16 transcripts (**Figure S5ab**), such as for transcripts MYO5A-204, MYO5A-205, MSTRG.8280.2, MSTRG.8280.3, MSTRG.8280.11, and MSTRG.8280.12. The only exception is MYO5A-201, for which expression in EU pigs was significantly higher than in AS pigs (FC=2.15, adjusted p-value ≤ 0.05). It is remarkable to note that this 303 bp MEI/DEL overlaps with alternative splicing for MYO5A-202, as shown in **Figure 5b**. We speculated that similar overlapping thus alternative splicing situations may occur for MSTRG.8280.1, 2, 3, 4, 6, and 7. Especially for MSTRG.8280.3, As pigs had significantly higher absolute transcript levels and ratios, as compared to EU pigs (**Figure S5ab**). All these results made us speculate the 303 bp MEI/DEL might influence the MYO5A transcript splicing and expression, thus resulting in the coat color difference among these four pig breeds. For example, it is possible that this new MEI brought in a new untranslated region, which broke MYO5A-201 into the two disconnected regions, leading to the creation of two short transcripts of MYO5A-202 and MYO5A-206 (**Figure 5a**). The changes in splicing patterns and expression level of the *MYO5A* transcripts may be related to altered protein level and melanosome transport, suggesting a potential mechanistic link between the MEI and the coat color phenotype. Future experiments will be needed to validate this hypothesis.


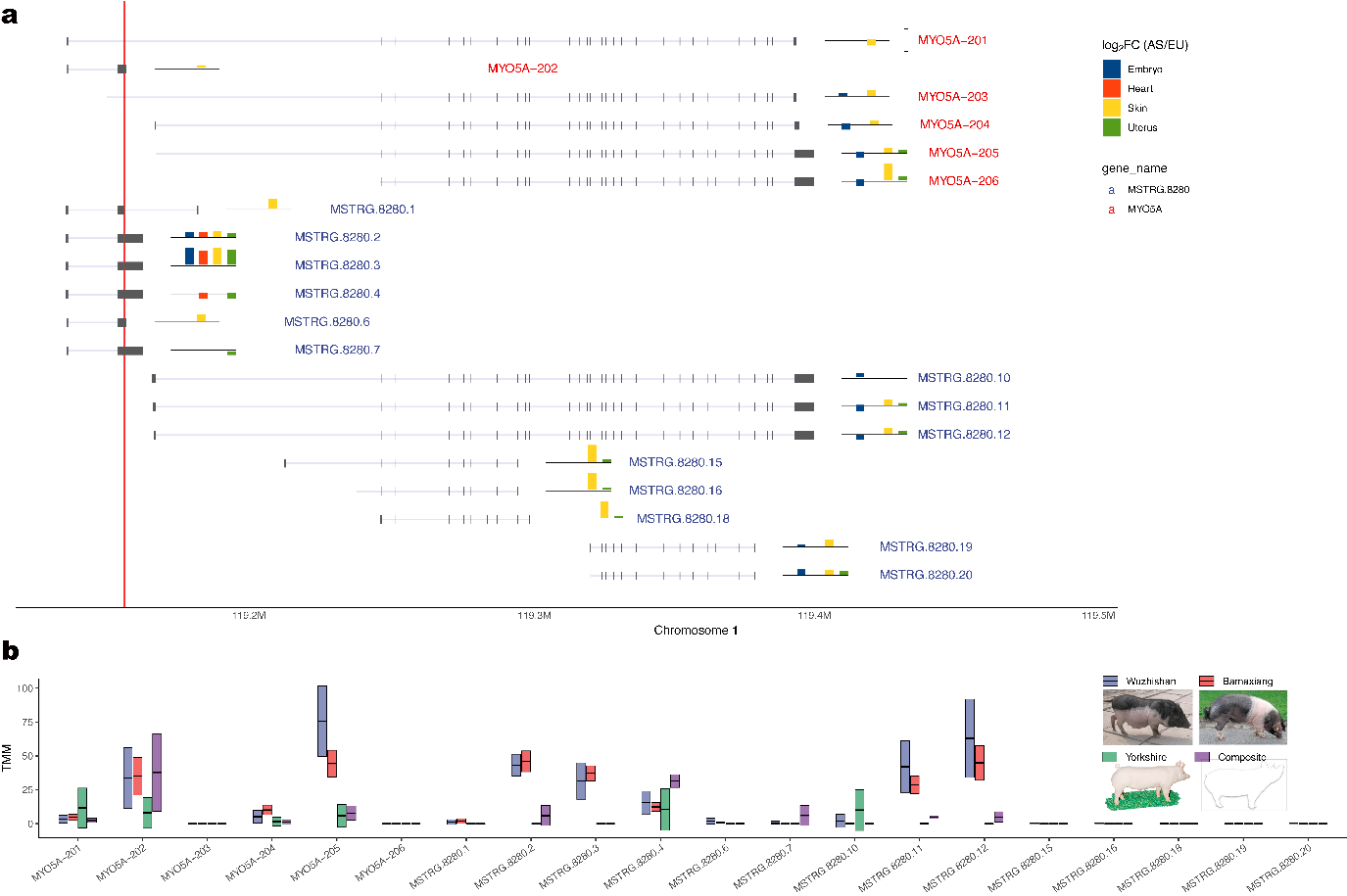


### Figure S6. Reassembly of the *MYO5A* gene transcripts.

### **a.** Reassembly of the *MYO5A* gene transcripts. The bar and line on the right of each transcript indicate the log_2_FC of the normalized expression of AS and EU (AS/EU). Bars on the top of the line denote higher transcript expressions in AS compared to EU.

**b.** Expression levels of MYO5A transcripts from official annotations and reassembled transcripts MSTRG.8280s.

### 8. Line 399: “We detected a strong-effect copy number gain (Yorkshire-specific DUP of 55,665 bp, 8:130924619-130980283) within ABCG2, which could potentially down-regulate its expression in blood, embryo, and uterus in Yorkshire as compared to Duroc.”

What is meant by “strong-effect”? When one speaks of CNV, effects can reflect the number of copies gained or biological effects, including gene expression or physiological impact. If it is a many-copy number gain, it seems straightforward to say that, and, if there is an estimate of minimum or most likely copy number, that is useful to know.

AU: By “strong effect”, we mean the DUP disrupted a TAD in the 3D genome structure. This DUP did occur within the coding sequence of ABCG2, leading to its decreased expression. Furthermore, this DUP did not seem to affect the expression of its neighbor gene *PKD2* (7,011 bp downstream of *ABCG2*) but led to the increased expression of *SPP1* (79,547 bp downstream of *ABCG2*) in adipose, embryo, and uterus in Yorkshire when compared to Duroc (**Figure S6**).

### 9. Lines 403 and 1126 “Fig 6b. Illustration of the genomic region for copy number gain of ABCG2.” This figure is difficult for me to interpret because it is one genomic interval illustrating two structurally variant versions. For the gene/mRNA and TAD structure, it would be simpler to show both states separately so they/the evidence can be visually compared. The panels at the very bottom have very faint data that was impossible for me to interpret visually.

AU: Hi-C/TAD data were generated by the FANNG project from Yorkshire. This DUP was also found to be Yorkshire-specific. After mapping them onto the Duroc pig reference genome, TAD was disrupted by the DUP. As all tracks are based on the Duroc pig reference genome assembly, we cannot easily LiftOver all of them to a version of assembly with this DUP, if exists. We deleted the IGV panel to improve the figure quality.

### 10. Discussion: Line 441 “Although short-read sequencing presents challenges in accurate identification of SVs, it is currently the most practical way to assess SV diversity”. It would be useful for readers to understand similar work going forward will probably use long read methods (or combination of the two). PacBio is used for validation purposes here. The authors could say in one or two sentences what that transition will be like (as it has been done in other species).

AU: We added “As shown in humans and other species [69-73], the two emerging trends are the so-called T2T complete sequence of a genome and the construction of pangenome in one species, which are both tightly linked to SV events. Additionally, direct SV-based e/sQTL mappings using short reads and long reads will be warranted, as shown recently in humans [64, 74, 75]”

11. Discussion: Line 493: Similarly to Results of the same: The MYO5A discussion is confusing and – if I understand correctly – unconvincing. It is first discussed as a deletion, at the end of this MYO5A section, the authors say they changed their mind – that it is not a deletion in deletion SV carriers but a likely mobile element insertion in non-carriers. Again, Table S32 shows something like 30 breeds fixed for non-deletion SV and 50 fixed for deletion SV – how can there be no mention of coat color/pattern differences between the two groups? As a result, one of a total of only two SV-gene biological findings mentioned in the Abstract is unsatisfying for both the molecular genetics of the SV and the candidate relevant-physiology (and I am confused as to why that is since they have RNAseq data to report the quantitative and qualitative effects and presumably have the coat phenotype data for all the breeds used).

AU: See our answer to Q7 above.

### 12. Methods: Line 528: “A total of 1,208 WGS data with at least 10× coverage were retrieved from the data collected for the FarmGTEx project for pigs (PigGTEx) [33].” As far as I can tell, the work refers to the genetic data it is based on as “short read sequence” and “paired end”, but I couldn’t find a description of any other detail (read length, sequencing platform, etc.). Ref. 33 is a bioRxiv preprint: it states that details of the WGS are in Suppl. Table 2, but I could not find such a table on that site, and could not find description of the WGS in the pre-print.

AU: We provided Table S43 for all these WGS. All data used in this study were labeled.

### 13. Given the species studied and, say, George Liu’s participation (who studies cattle CNV), I was disappointed there was no discussion of the domestication angle – e.g., for the domesticated species with a body of knowledge of SVs, how do they compare for numbers of SVs or biochemical pathways affected, and signatures of selection? Similarly, it seems likely there is reason to use the deep knowledge of human SVs to glean insights, but this doesn’t appear to have been done thoroughly. I understand this was likely because the present approach was to push the limits of all available pig resources – which are many. However, I found that effort to be so confusing that I didn’t know what firm knowledge was gained (i.e., as asked several times above, is it all positional correlations of SVs and phenomena in any pig population, or is it SV haplotypes associated with specific traits in specific pig populations).

AU: Comparative SV analysis across different species, such as humans, cattle, and pigs, can provide valuable insights into evolutionary dynamics, genetic diversity, and functional implications. However, as Reviewer 1 pointed out, our plate was already very full. We will leave it for another dedicated effort. We added the following summary to Discussion:

“Comparing SVs in humans, cattle, and pigs will advance our knowledge of genome evolution, genetic diversity, functional genomics, and complex diseases and traits. This effort could offer a comprehensive view of how genomic landscapes adapt to environmental factors across species. It will uncover both conserved regions and specific SVs linked to each lineage, providing insights into the genetic mechanisms driving species evolution and breed development. SVs are pivotal in shaping genetic diversity, via processes like recombination, replication errors, or mobile element insertions. Studying SVs will help reveal their impact on gene regulation, protein function, disease susceptibility, and the varied phenotypes observed.

Both cattle and pigs have undergone extensive selective breeding to produce various breeds tailored for specific purposes such as meat production, disease resistance, and reproduction. The diversity within them provides a rich genetic resource for studying traits relevant to growth rate, meat quality, milk production, disease resistance, and reproductive performance. For instance, cattle genomes harbor unique genes associated with specific adaptations, such as genes for rumen development and digestion of cellulose-rich diets. Additionally, certain gene families related to immune, lactation, metabolism, and even production traits are more diverse or unique in cattle. Conversely, in pigs, genes associated with immune response and olfaction show rapid evolution. Pigs have the largest repertoire of functional olfactory receptor genes, reflecting the importance of smell in this scavenging animal.”

Minor comments
1. Suppl figs. Would be improved by providing fig. # and legends on the same PDF

AU: It was provided with all supplemental figures and their legends.

2. Abstract: first sentence: “large regions of DNA sequence” presumably was intended to mean large “amounts” vs large “regions”. Also line 99: “This form of variation involves larger segments of the genome”: larger “segments” should be a larger “proportion”

AU: Revised.

3. Abstract: third sentence: “It covers 9.6% of the pig genome”: use of “covers” may confuse some readers vs. SVs “comprise” 9.6%

AU: Revised.

4. Line 102: “observed to range from 50 base pairs (bp) to 5 megabase pairs (Mb)”. I think “observed” here is intended to mean “defined as” 50 bp or more (and generally refers to up to 5 Mb but has no upper limit)

AU: Revised.

5. Line 103: “The large size of SV increases the likelihood that SV might impact gene expression and function”. I think that intends to say “With increasing size of SVs, the likelihood of impacting genes and their expression increases”

AU: Revised.

6. Line 151: “we totally discovered and genotyped 130,556” should be “we discovered and genotyped a total of 130,556”

AU: Revised.

7. Line 155: “The lengths of DEL, MEI, DUP, and INV were 96.3, 16.6, 61.3, and 59.6 Mb, respectively, covering 9.6%” should be something like “The sum of the lengths of…, constituting 9.6%”

AU: Revised.

8. Line 171: “A large part” should be “A large portion”

AU: Revised.

9. Line 203: “enriched in 23 Gene Ontology (GO) terms, such as membrane protein complex, cell junction, kinase activity, and ATP-dependent activity (Table S7).” It is not clear why three of the four chosen are among the weakest by P adj (marginally significant). I believe mentioning the top four as follows makes more sense: phosphorylation (4 of 5 top terms; starting with P adj= 2.38E-06), exopeptidase activity, ion transmembrane transporter activity, and cytoskeletal protein binding

AU: Revised. We rewrite it to “Those SV-overlapped genes were significantly (Bonferroni adjusted P < 0.01) enriched in 23 Gene Ontology (GO) terms, such as phosphorylation (2 top terms; starting with an adjusted P value of 2.38x10^-6^), kinase activity, exopeptidase activity, and ion transmembrane transporter activity.”

10. Line 204: Similarly to preceding comment: Table S8 has 8 significant GO terms, but 5 are phosphorylation/kinase terms and 3 are anion/chloride channels/transporters. Thus, it is straightforward to concisely describe the results more thoroughly and clearly

AU: Revised. Changed sentence to “Among them, genes overlapped with singleton SVs were related to 5 phosphorylation/kinase terms and 3 anion/chloride channels/transporters”

11. Line 204: “Among them, genes overlapped singleton SVs” should say “gene-overlapping singleton SVs”

AU: Revised.

12. Line 234: “Of them, the highest partitions” should be “Of those…” and, I believe the intention is to continue “… the highest proportions”. Line 305 also has “Of them” which should be Of those

AU: Revised.

13. Line 410, “Intriguingly, this DUP leaped over its neighbor gene PKD2”. It is not clear what “DUP leaped over its neighbor” was intended to mean. In Line 509 in Discussion, it is clear it refers to the SV not affecting the expression of the closest downstream gene but influencing that of the next one after that.

AU: Revised. Changed it to “this DUP did not seem to affect the expression of its neighbor gene PKD2 (7,011 bp downstream of ABCG2) but led to the increased expression of SPP1 (79,547 bp downstream of ABCG2) in adipose, embryo, and uterus in Yorkshire when compared to Duroc.”

14. Line 465: “The occurrence of MEIs… indicates that SVs may be critical agents for gene expression pleiotropy that is often observed in stress-responsive genes.” Please provide ref. for stress-responsive genes statement

AU: Revised. We added <https://pubmed.ncbi.nlm.nih.gov/23293987/> as ref [57].

**Comments from Reviewer #2:**

The paper by Yang et al is certainly comprehensive and represents the largest and most complete - also in terms of pig diversity considered - to date. In fact it may be a landmark paper now that we are moving on to the next phase of variation analyses using long reads and pangenomes. For pigs, this paper may represent the best that could be done using short reads.

So, I have no doubt the paper has potential to be cited a lot. Still I am somewhat conflicted since there is relatively little biological insight coming from the paper despite the deep integration of structural variation analysis and functional data. Authors do provide lots of results but it is not always very clear what the significance is, and it is also not always easy to take in the results to begin with. Figure 4b is a good example of such a presentation of results - very interesting, but where to begin with reading it and what exactly to take home from it? Given the broad readership of the journal this is perhaps a concern, although I leave that to the editor to decide.

The paper has lots of value as a resource paper - again, it could be seen as a landmark paper. But then it is very important that ALL data is easily accessible publicly.

AU: All data have been released to the public. The main message is this study was to provide a comprehensive SV catalog across global pig breeds using extensive whole-genome sequences followed by studying their potential functional impacts on pig molecular and complex traits through examining the PigGTEx results.

Part of the data is already in public databases, but - maybe I've missed it - in most cases there are no database identifiers noted in the Sup tables. I'm also concerned about the functional data sources - expression data especially - since unclear to me that it can be retrieved from a public database. For the paper to really have value it is important that all these data are not only in public databases but also easily retrievable with no gatekeeping system.

Lastly I'm very concerned about the layout of most of the figures. Figures are cramped and often not easy to comprehend. I'm not sure how they would end up in print.

AU: All data have been released to the public. We have also improved the figure quality.

Some smaller points:

L 116-121 In the discussion on defining CNVs/large with short read sequencing: long read sequencing such as Nanopore sequencing makes such issues largely obsolete. It is ok to discuss this but this should probably be mentioned at least.

AU: We added a few sentences about the impacts of incoming long-read sequencing on SV discovery in Discussion (L632-636).

L 131: "Interbred hybridization", should probably better be "crossbreeding".

AU: Revised.

L 528: "A total of 1,208 WGS data" - "data" should be "datasets"? For clarity, "WGS data" should be replaced with "whole genome short-read sequence datasets".

AU: Revised.

L 575: Provide list of 'bad bins'.

AU: Provided in Table S44.

L594: NGS? Short read sequencing?

AU: Revised. We removed NGS and used short read instead.

Breakend (BND) poorly defined in the paper. Since this is a term that is not unambiguously used in the literature, authors should a) define it properly and unambiguously, and b) be explicit which part of the analysis particularly supports BND.

AU: Breakend (BND) represents a discontinuity in the sample genome alignment with respect to the reference and can indicate the presence of an unresolved structural variant in the results of SVtools [17]. This can result from ambiguity when looking at the different categories of evidence at a certain site or from assembly issues. Because BND was defined as a generic rearrangement of unknown architecture, we focused on other well-defined SV types for all subsequent analyses.

KIT example is confusing; maybe because it seems that Fig S5 is truncated (?). KIT structural variations have been well described from Yorkshire and Pietrain, so I'm wondering why these were not included, while Duroc is (although many Duroc nowadays are in fact white, traditionally they lack the SV).

AU: As we reviewed before [7], mutations of KIT are associated with the dominant white coat color of several mammalian species. For instance, duplications of regulatory elements upstream and downstream of the KIT gene locus resulted in a belted phenotype in Yorkshire and Pietrain pigs. Subsequent duplication of this altered KIT gene locus, in addition to a splice site variant that excludes exon 17, results in the dominant white phenotype[18]. We double checked and found a DUP allele in Yorkshire and Piebald pigs (**Figure S5**). Especially, in the Yorkshire pig, multiple duplications were also observed both upstream and downstream of the *KIT* gene.

In the Integrative Genomics Viewer (IGV), aligned reads were derived from three randomly selected individuals each belonging to six different breeds: Erhualina, Meishan, Wuzhishan, Bamaxiang, Pietrain, and Yorkshire. Notably, the red box and background highlight the genomic region corresponding to a whole-gene duplication (DUP) at coordinates 8:41223207-41783659.


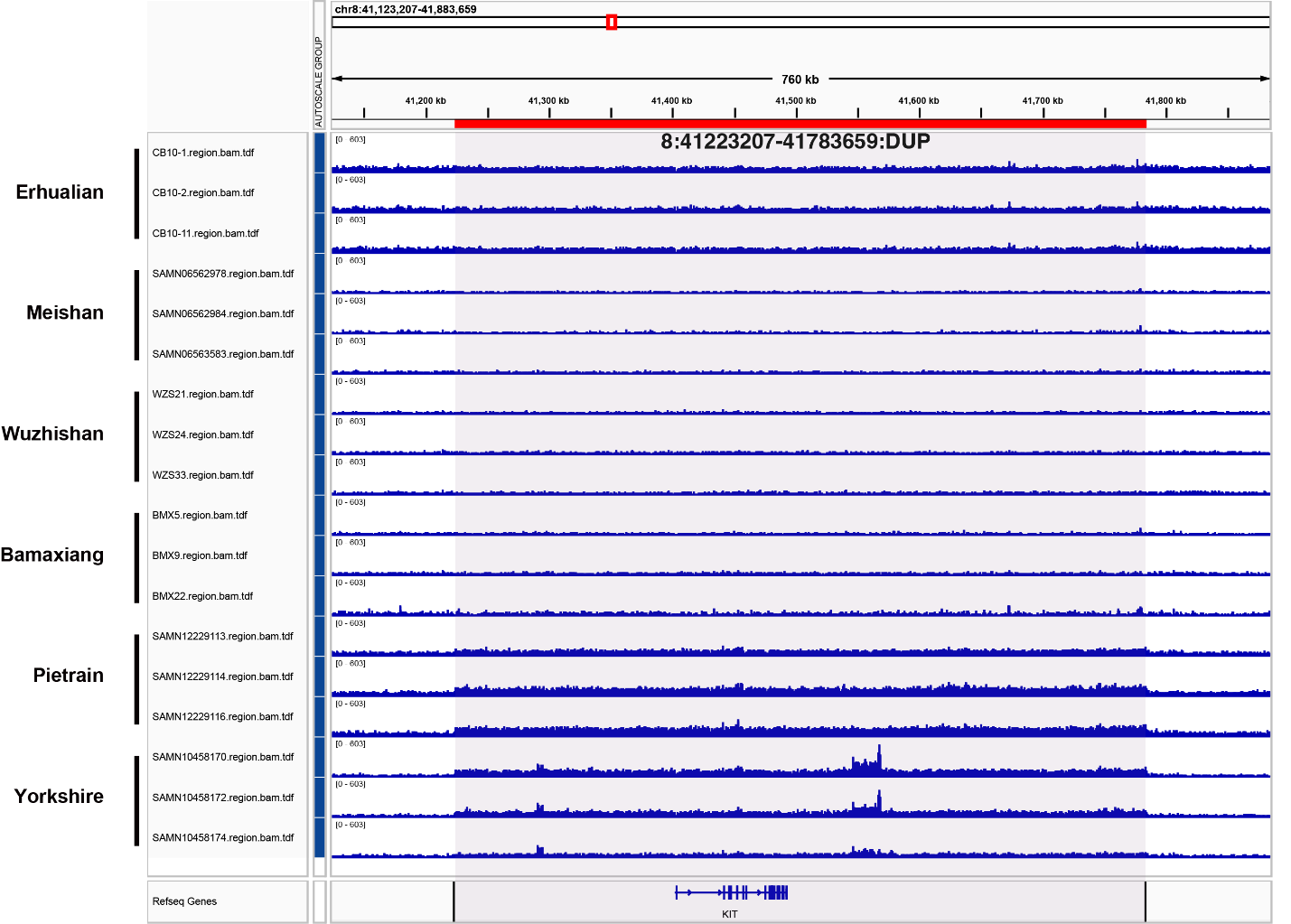


**Figure S5. Illustration of the genomic region for whole gene DUP of KIT.**

1. Abel HJ, Larson DE, Regier AA, Chiang C, Das I, Kanchi KL, Layer RM, Neale BM, Salerno WJ, Reeves C *et al*: **Mapping and characterization of structural variation in 17,795 human genomes**. *Nature* 2020, **583**(7814):83-89.

2. Collins RL, Brand H, Karczewski KJ, Zhao X, Alföldi J, Francioli LC, Khera AV, Lowther C, Gauthier LD, Wang H *et al*: **A structural variation reference for medical and population genetics**. *Nature* 2020, **581**(7809):444-451.

3. Chiang C, Scott AJ, Davis JR, Tsang EK, Li X, Kim Y, Hadzic T, Damani FN, Ganel L, Montgomery SB *et al*: **The impact of structural variation on human gene expression**. *Nat Genet* 2017, **49**(5):692-699.

4. Kojima S, Koyama S, Ka M, Saito Y, Parrish EH, Endo M, Takata S, Mizukoshi M, Hikino K, Takeda A *et al*: **Mobile element variation contributes to population-specific genome diversification, gene regulation and disease risk**. *Nature Genetics* 2023, **55**(6):939-951.

5. The PigGTEx Consortium: **The pig Genotype-Tissue Expression (PigGTEx): a comprehensive atlas of genetic regulatory effects across pig tissues**. *Nat Genet In Press, Preprint at bioRxiv* 2022:2022.2007.2015.406280.

6. Mills RE, Walter K, Stewart C, Handsaker RE, Chen K, Alkan C, Abyzov A, Yoon SC, Ye K, Cheetham RK *et al*: **Mapping copy number variation by population-scale genome sequencing**. *Nature* 2011, **470**(7332):59-65.

7. Bickhart DM, Liu GE: **The challenges and importance of structural variation detection in livestock**. *Front Genet* 2014, **5**:37.

8. Dai X, Bian P, Hu D, Luo F, Huang Y, Jiao S, Wang X, Gong M, Li R, Cai Y *et al*: **A Chinese indicine pangenome reveals a wealth of novel structural variants introgressed from other Bos species**. *Genome Res* 2023, **33**(8):1284-1298.

9. Jiang YF, Wang S, Wang CL, Xu RH, Wang WW, Jiang Y, Wang MS, Jiang L, Dai LH, Wang JR *et al*: **Pangenome obtained by long-read sequencing of 11 genomes reveal hidden functional structural variants in pigs**. *iScience* 2023, **26**(3):106119.

10. Passols M, Llobet-Cabau F, Sebastià C, Castelló A, Valdés-Hernández J, Criado-Mesas L, Sánchez A, Folch J: **Identification of genomic regions, genetic variants and gene networks regulating candidate genes for lipid metabolism in pig muscle**. *animal* 2023:101033.

11. Crespo-Piazuelo D, Criado-Mesas L, Revilla M, Castelló A, Noguera JL, Fernández AI, Ballester M, Folch JM: **Identification of strong candidate genes for backfat and intramuscular fatty acid composition in three crosses based on the Iberian pig**. *Scientific Reports* 2020, **10**(1):13962.

12. Nurk S, Koren S, Rhie A, Rautiainen M, Bzikadze AV, Mikheenko A, Vollger MR, Altemose N, Uralsky L, Gershman A *et al*: **The complete sequence of a human genome**. *Science* 2022, **376**(6588):44-53.

13. Liao WW, Asri M, Ebler J, Doerr D, Haukness M, Hickey G, Lu S, Lucas JK, Monlong J, Abel HJ *et al*: **A draft human pangenome reference**. *Nature* 2023, **617**(7960):312-324.

14. Gao Y, Yang X, Chen H, Tan X, Yang Z, Deng L, Wang B, Kong S, Li S, Cui Y *et al*: **A pangenome reference of 36 Chinese populations**. *Nature* 2023, **619**(7968):112-121.

15. Chen J, Wang Z, Tan K, Huang W, Shi J, Li T, Hu J, Wang K, Wang C, Xin B *et al*: **A complete telomere-to-telomere assembly of the maize genome**. *Nature Genetics* 2023, **55**(7):1221-1231.

16. Belser C, Baurens F-C, Noel B, Martin G, Cruaud C, Istace B, Yahiaoui N, Labadie K, Hřibová E, Doležel J *et al*: **Telomere-to-telomere gapless chromosomes of banana using nanopore sequencing**. *Communications Biology* 2021, **4**(1):1047.

17. Larson DE, Abel HJ, Chiang C, Badve A, Das I, Eldred JM, Layer RM, Hall IM: **svtools: population-scale analysis of structural variation**. *Bioinformatics* 2019, **35**(22):4782-4787.

18. Rubin CJ, Megens HJ, Martinez Barrio A, Maqbool K, Sayyab S, Schwochow D, Wang C, Carlborg O, Jern P, Jorgensen CB *et al*: **Strong signatures of selection in the domestic pig genome**. *Proc Natl Acad Sci U S A* 2012, **109**(48):19529-19536.

**Second round of review**

**Reviewer 1**

The response to my suggestions is excellent and the manuscript is much improved. I have no further concerns.
